# A systematic review of T cell epitopes defined from the proteome of human immunodeficiency virus

**DOI:** 10.1016/j.virusres.2025.199602

**Published:** 2025-06-23

**Authors:** Yan Ding, Ling Huang, Yandan Wu, Jialai Yan

**Affiliations:** a Department of Clinical Laboratory, the Second Hospital of Nanjing, Affiliated to Nanjing University of Chinese Medicine, Nanjing, Jiangsu, China 210003; b Department of Microbiology and Immunology, Medical School of Southeast University, Nanjing, Jiangsu, China 210009; c School of Medical Technology, Anhui Medical College, HeFei, Anhui, China 230601

**Keywords:** Human immunodeficiency virus, HLA restriction, T-cell epitope

## Abstract

**Background:**

Human immunodeficiency virus (HIV) persists as a formidable and far - reaching threat without a cure. T cells are crucial for antiviral immunity and pathology in HIV patients, with specific T cell epitopes potentially key to effective therapies and HIV cure methods.

**Methods:**

Identifying sufficient T-cell epitopes within the HIV proteome holds great significance. It can not only substantially accelerate the development of T-cell epitope-based vaccines but also enable a highly precise evaluation of the host's HIV-specific cellular immunity. This research provides an overview of functionally verified T-cell epitopes derived from HIV antigens, the human leukocyte antigen (HLA) alleles, as well as the screening and identification strategies.

**Results:**

Totally, 239 and 82 epitopes have been verified for CD8^+^ T-cell and CD4^+^ T-cell respectively by functional experiments. The majority are presented by various HLA supertypes, such as HLA-B35, B5301, A6802 or A0201, and DRB1 molecules. Furthermore, 74 % of the epitopes for CD8^+^T-cell belong to Gag, Pol, as well as Nef Protein while 68 % of the CD4^+^ T-cell epitopes originate from Gag protein. Antigenic peptides of HIV-1 subtypes A/B/C/D/CRF01_AE account for 11.43 %, 58.26 %, 21.69 %, 4.96 %, and 3.65 %, respectively.

**Conclusions:**

The 321 T-cell epitope repertoires of HIV encompass the HLA polymorphisms of the main populations and subtypes in a particular geographical area. These epitope catalogs provide strong support for researching therapeutic vaccines, specific T-cell detection, and the interaction mechanism between HIV and the immune system. However, the limitations of the identified T-cell epitope library, the polymorphism of HLA molecules, and the high mutation rate of HIV require more research to cover the entire HIV proteome and the comprehensive landscape of T-cell epitopes in global patients.

## Highlights

• Human immunodeficiency virus (HIV) remains a global health threat, and there is no available curative therapy currently.
• Host T cells have been proved to play a vital role in antiviral immune protection and pathology among HIV patients.
• The 321 T-cell epitope repertoires of HIV encompass the HLA polymorphisms of the main populations and subtypes in a particular geographical area, which seriously impedes the development of vaccines and specific T-cell detection systems.
• This manuscript offers a comprehensive review of T-cell epitopes covering the entire HIV proteome and global patients.
• This knowledge provides valuable insights for future studies on the development of T-cell epitope vaccines and the accurate assessment of host HIV-specific cellular immunity.

## 1 Introduction

AIDS (Acquired immunodeficiency syndrome) is resulting from infection with the human immunodeficiency virus (HIV) (Lee and Crotty, 2021). In accordance with the Joint United Nations Program on HIV/AIDS (UNAIDS) in August 2023, there are currently 39 million people with HIV/AIDS worldwide, with approximately 29.8 million individuals undergoing antiretroviral therapy by the end of 2022. Due to the large number of HIV variants and the existence of reservoirs, there is still no effective vaccine. The specific immune response against viral infections is mediated by both humoral and cellular immunity. While specific antibodies can neutralize extracellular free HIV virus, the main mechanism of immune response against intracellular HIV involves specific cellular immune suppression and elimination of infected cells (Kalams et al., 1999). Studies have proved the significant association of HIV-specific T cell responses with the decelerated progression of the disease in HIV-1 exposed seronegative individuals (Bernard et al., 1999; Kaul et al., 2000; Kaul et al., 2001),elite controllers (Pancré et al., 2007), and long-term non-progressors, specifically, CD8^+^ T cells, as key effector cells, are responsible for the elimination of cells infected by the virus and the production and release of cytokines. However, in the majority of individuals infected with HIV, chronic viremia develops gradually without treatment. The persistent stimulation of HIV-specific CD8^+^ T cells by continuously present HIV antigens causes the loss of their functional capabilities, resulting in exhaustion and further impairing the control of HIV infection (Fenwick et al., 2019). The major characteristics of CD8^+^ cell exhaustion include impaired proliferative capacity, diminished secretion of effector molecules(CD107, IL-2, and IFN-γ), upregulation of a series of co-inhibitory receptors (PD-1, TIM3, etc.), and the inability to form protective memory responses (Wherry, 2011; Hashimoto et al., 2018). Therefore, further in-depth research on HIV-specific T cells is not only a potential marker for monitoring disease progression and predicting antiviral treatment outcomes but also holds potential implications for novel HIV remission strategies. Identifying a large number of T cell epitopes from HIV antigens is essential for preventing and reversing the exhaustion of HIV-specific CD8^+^ T cells. It is of significance in enhancing their effector functions, developing therapeutic vaccines centered on HIV-specific T cells, and attaining a functional cure for AIDS.

In this review, epitopes for CD8^+^ and CD4^+^ T cells that define the function of the HIV proteome were systematically assembled. The data was sourced from English journal articles indexed in databases including PubMed, Scopus, Embase, SinoMed, and Google Scholar. The online search that was carried out most recently was on November 13, 2024. "T cell epitope" and "HIV or human immunodeficiency virus " were employed as search terms. 1638 studies were found from multiple databases and through manual exploration during the preliminary search. These articles were all imported into Endnote X9 software. Subsequently, 102 duplicate entries were eliminated. From 1990 to 2024, a total of 789 studies were collected. Subsequently, 695 articles were selected via the screening of abstracts and full-texts. The exclusion criteria are as follows: (1) lack of relevance to the screening or identification of T cell epitopes; (2) sole used for computational or structural bioinformatics analysis, without being used for cellular functional experiments, tetramer staining, binding assays or immunogenicity; (3) incomplete details about the sequences of epitopes; (4) presented by 4-digit class I allele(s). Finally, this review utilized and cited 296 articles.

## 2 Polymorphism of HLA alleles and association with HIV infection

HLA molecules consist of class I and class II molecules. Endogenous viral epitopes are presented to specific CD8^+^ T cells by Class I HLA molecules, whereas exogenous viral peptides are presented to specific CD4^+^ T cells by Class II HLA molecules (Elangovan et al., 2021). Naïve CD8^+^ T cells initiate a process of activation, proliferation, and then differentiate into cytotoxic T lymphocytes (CTLs). This transformation enables them to induce the lysis of virus-infected cells. Naïve CD4^+^ T cells undergo differentiation into Th1 or Th2 cells. The former facilitates the activation of virus-specific CD8^+^ T cells, whereas the latter promotes virus-specific B cells in antibody production (Nitschke et al., 2016). However, HLA molecules exhibit high levels of polymorphism among global populations, with different individuals carrying different HLA genotypes and presenting different antigenic peptide epitopes. This polymorphism determines the variation in immune responses to HIV among different individuals. According to the International ImMunoGeneTics information system/Human Leukocyte Antigen (IMGT/HLA) database (https://www.ebi.ac.uk/ipd/imgt/hla/), by November 2023, a total of 37,516 HLA class I and II gene loci alleles have been identified worldwide, including 8012 HLA-A, 9573 HLA-B, 7513 HLA-C, 4530 HLA-DRB, 2491 HLA-DQB1, and 2393 HLA-DPB1 alleles. Furthermore, the distribution of HLA alleles varies significantly among various racial populations and geographical regions. This diversity implies that different populations may exhibit distinct immune responses to specific pathogens (Elahi and Horton, 2012; Wang et al., 2016). Therefore, the diversity of HLA genotypes is an important aspect when studying the genetic factors influencing HIV/AIDS disease progression.

Researches have revealed associations between the polymorphisms of HLA and the onset of specific diseases (Boeijen et al., 2017; Littera et al., 2020). Despite the fact that the association between HLA alleles and HIV infection is not yet fully comprehended, past and ongoing research continues to elucidate this relationship. The HLA supertype A2 is widely recognized for its relationship with the prevention of HIV infection (Rallón et al., 2017; Li et al., 2017). The HLA-B locus exhibits the most diverse allelic genes, and HLA-B might offer greater protection against HIV- 1 compared to HLA-A (Rajapaksa et al., 2012). Specifically, B57, B27, and B7 are supertypes that are closely associated with the disease progression (Brener et al., 2015; Singh et al., 2008; Tang et al., 2010; Altfeld et al., 2003). Evidently, HIV-specific CD8^+^ cell response is primarily linked to HLA-B57 and is related to the slow progression of AIDS (Stephens, 2005; Kaslow et al., 1996; Dorak et al., 2003; Gao et al., 2001; Tang et al., 2002; Lobos et al., 2022). The HLA-B7 supertype is associated with high viral load and relatively poor CTL response in Caucasians and African Americans primarily infected with HIV-1 subtype B, leading to rapid disease progression. However, this association is not present in Africans infected with HIV-1 subtype C (Pereyra et al., 2010; Peterson et al., 2013). HLA-B27 has been found to be related to low viral load and delayed onset of AIDS in Caucasians infected with HIV-1 subtype B, but this association does not hold true for Africans (Gao et al., 2001; Trachtenberg et al., 2003; Flores-Villanueva et al., 2003). Despite numerous researches being conducted to explore the correlation between HIV infection and HLA allele genes, as described in the above review, contradictory results are often present, and these variations are partly caused by the HLA polymorphism of hosts from different races and regions (Matern et al., 2020; Zidi et al., 2016). Thus, further research in various regions is urgently required to reduce the result heterogeneity. Since the advancements in HLA typing techniques have strengthened the capacity to recognize the associations between HLA and the outcome of HIV disease, numerous new alleles are identified, such as HLA-B Bw4, B35, B39, B18, B08 and A29 (Kuse et al., 2021; Ramírez de Arellano et al., 2019; Zhang et al., 2018). Recently, HLA-C*03:02 and HLA-C*14 subtypes have the potential to impact viral control in cases of HIV-1 infection (Kyobe et al., 2021; Chikata et al., 2022). Reported alleles to include HLA-DQB1*03:02, HLA-DQB1*06:04 and HLA-DQB1*02:01 have been related with rapid disease progression and high risk of HIV infection, while HLA-DP1 *01:01, HLA-DP1*03:01, HLA-DRB1*01:01 and HLA-DRB1*01:02 are related with slower disease progression and a protective effect against HIV infection (Just et al., 1995; Just et al., 1996; Achord et al., 1997). Research on the correlation between HLA-C and HLA class II molecules is relatively limited. For future studies, it is necessary to include a more extensive range of HLA molecules so as to enhance the consistency and dependability of the results.

## 3 HIV protein

HIV is a type of retrovirus with two main subtypes, HIV-1 and HIV-2. HIV-1 can be distinguished into HIV-1 subtypes M (major), O (outlier), N (non-M, non-O), and P, with distinct global distribution patterns (Hemelaar et al., 2006; Osmanov et al., 2002). Its structure is approximately spherical, and the virus particle consists of an envelope, a capsid, and a core. HIV genome contains three structural genes, namely Gag, Pol, and Env; two regulatory genes, Tat and Rev; and four accessory genes, Nef, Vpr, Vif, and either Vpu (specific to HIV-1) or Vpx (specific to HIV-2). Proteins translated from the HIV genome play specific roles in the lifecycle of HIV. Gag gene, a highly conserved region of the HIV virus and with relatively low sequence variability among different HIV strains, has been demonstrated as an ideal target for candidate vaccines (Williamson and Rybicki, 2016). Pol gene, codes for the precursor enzymes of reverse transcriptase, integrase, and protease. Env gene, is the main target of the host humoral response, including neutralizing antibodies and Fc-dependent cell-mediated mechanisms (Wang et al., 2020). Tat protein is capable of activating T cells through mediating both classical and non-classical activation pathways within T cells (Sacks et al., 2018). Rev is expressed during the early stages of virus replication (Rausch and Le Grice, 2015). Nef exhibits high levels of expression during the course of infection. It manipulates the trafficking of multiple cell-surface proteins, aiming to disrupt the viral replication cycle (Buffalo et al., 2019). Vpr is believed to serve as a carrier for delivering antiviral molecules to viral particles and is also a potential therapeutic target for blocking HIV disease (Höhne et al., 2016). A deep understanding of the structure of HIV and the mechanism affecting immune cells is crucial for the advancement of both HIV vaccines and novel treatment modalities.

## 4 Strategies for screening T-cell epitopes

### 4.1 Selection of epitopes by bioinformatics

The development of bioinformatics has provided us with many potential applications for epitope prediction. Currently, over 30 different bioinformatics tools and databases are used for the prediction of epitopes (Sánchez-Burgos et al., 2021; Vita et al., 2025; Robinson et al., 2024). This computational prediction is rapid and convenient, requiring no expensive or highly complex instruments, and has been widely used for the identification of epitope in mycobacterium tuberculosis (Yun et al., 2024), vaccinia virus (Samantaray et al., 2025; Mamun et al., 2024), and cancer (Xu et al., 2024; T et al., 2024). Different methods or algorithms are employed by each bioinformatics tool to predict epitopes, leading to different levels of accuracy. The utilization of epitopes predicted by computer algorithms to bind to specific HLA alleles can reduce the number of subjects with diverse HLA subtypes. However, the theoretical inaccuracy of these predictions might cause some real-world epitopes to be overlooked. However, it has been reported that using multiple tools for epitope prediction can improve prediction accuracy (Bonsack et al., 2019). Furthermore, given the understanding of protective HLA allele genes in HIV controllers, bioinformatics methods can be used to select epitopes specific to protective HLA allele genes. In a recent investigation, researchers conducted an assessment of the NetMHCpan4.0 computational neural network's performance. They did this by attempting to re-identify 93 T-cell epitopes. Remarkably, it accurately predicted 88 out of the 93 experimentally mapped epitopes in 6 Ugandan individuals with HLA class I alleles who were infected with HIV-1 (Bugembe et al., 2020). Therefore, leveraging bioinformatics methods to narrow down the search space represents a highly rational and strategic approach. Due to the pre-designed program of computer, there may be some selection bias in predicting peptides. In order to reduce this bias, several methods will be selected to predict at the same time in general operation to select the best result.

There is another epitope prediction based on T cell receptor (TCR) recognition, which is receiving increasing attention due to its wide application in immunotherapy, especially in personalized cancer treatment (Garcia-Garijo et al., 2019; Gartner et al., 2021). Ogishi proposed a computational framework to simulate the thermodynamic interactions between pMHC complexes and public TCR clones to assess immunogenicity (Ogishi and Yotsuyanagi, 2019). Pham et al. used only CDR3β and peptide sequences to generate TCR-peptide predictions (Pham et al., 2023). Lin introduced a paired energy model RACER for high-throughput evaluation of TCR-peptide affinity at the immunoglobulin library level, accurately estimating the recognition rate of tumor-associated neoantigens and foreign peptides (Lin et al., 2021). Currently, structure-based methods present significant computational challenges. They attempt to predict epitopes recognized by T cell receptors rather than focus solely on the potential for binding to HLA molecules. Due to the continuous development of databases, machine learning algorithms and progress in the field is expected to be rapid.

### 4.2 Overlapping peptides

Overlapping peptides (OLPs) that span the entire proteome or specific target antigens (peptide scanning) are widely used in the screening of viral epitope peptides, especially CD4^+^ T-cell epitopes (Hoogeveen et al., 2022). While this method provides a comprehensive survey of T-cell epitopes within the target protein, it does not reveal the parameters that dictate epitope selection. Compared to computer-based prediction methods, peptide scanning is a costly and labor-intensive approach. Recently, researchers employed 17 - mer OLPs covering the Nef, Gag, and Pol regions of HIV - 1 subtype A/E consensus sequences to identify six T cell epitopes (Nguyen et al., 2022).

### 4.3 Validation of the immunogenicity of candidate epitope

Over the past two decades, significant progress has been made in the ways for validating the immunogenicity of candidate epitopes. At present, commonly used methods are as follows: cytotoxicity assays, intracellular cytokine staining (ICS), enzyme-linked immunospot assays (ELISpot)/fluorescence immunospot assays (FluorSpot), and polypeptide-MHC multimer staining. Each of these methods has its own unique set of advantages and disadvantages when considering factors such as practicality, cost, sensitivity, and functional evaluation.

Cytotoxicity assays directly or indirectly measure the release of lytic substances from targeT cells, such as chromium (McCoy et al., 1973) and lactate dehydrogenase (Decker and Lohmann-Matthes, 1988). For example, the chromium release assay has been used to detect virus-specific lysis of HIV-1-infected CD4^+^ T cells (Ranasinghe et al., 2016). There have also been reports using live cell imaging methods. For instance, quantitative live cell imaging of targeT cells expressing fluorescent dyes has demonstrated virus-specific lysis of SARS-CoV-2-infected cells by T cells (Wagner et al., 2022). Currently, T cell assays are based on cytokines produced by activated T cells after peptide stimulation such as ICS or ELISpot/FluoroSpot. In these assays, candidate peptide epitopes are used to stimulate patients’ PBMCs either *in viro* or *ex vivo*. ICS along with flow cytometry are utilized to ascertain whether CD4^+^ or CD8^+^ T cells are activated while ELISpot or FluoroSpot technologies are unable to make such a determination. ELISpot or FluoroSpot technologies are more sensitive than ICS, which detect single-activated cells within a population of 1 million PBMCs. Due to their accuracy, sensitivity, reproducibility, and robustness, these technologies have found extensive application and hold promising potential in the clinic (Portilho et al., 2022). Tetramer staining has long been regarded as the gold standard for highly sensitive and precise quantification of antigen-specific T cells and is therefore commonly used for epitope identification. Nevertheless, tetramers or multimers is costly, complex, and time-consuming. Novitsky et al. identified the highly immunogenic peptides derived from HIV-1 Gag, Tat, Rev, and Nef by tetramers and recommended incorporating these HIV-1 peptides as fundamental structural elements in the design of a multi-epitope HIV-1 vaccine (Novitsky et al., 2001).

Besides functional cell assays, peptide-HLA molecule binding assays are frequently utilized for epitope identification. Recently, Ferretti and others employed high-throughput whole-genome screening technology (T-Scan) to identify target cells presenting candidate epitopes. They found 29 epitopes for 6 of the most common HLA types in SARS-CoV-2 (Zhang et al., 2021). Chikata et al. employed immunocapture and liquid chromatography-mass spectrometry (LC-MS) to identify HIV-1 epitope peptides presented by HLA-C1202 (Chikata et al., 2019) and HLA-C*14 (Chikata et al., 2022) after pretreating the target protein, enabling large-scale characterization of the presented epitopes. Nevertheless, these alternatives are expensive and exhibit reduced detectability for peptides that are present at low frequencies. A variety of methods for identifying epitopes have been employed, each possessing distinct attributes and offering unique possibilities. Further technological developments will increasingly simplify high-throughput epitope characterization.

## 5 Defined T cell epitopes in HIV proteins over the last 33 years

Table 1, Table 2 respectively display the CD8^+^ and CD4^+^ cell epitopes and their HLA restrictions that have been defined from the HIV protein. Additionally, these epitopes’ references and the techniques or methods employed to confirm their peptide immunogenicity were all listed (Table S1/S2). Notably, CD8^+^ cell epitopes compiled in this review have a length of 8 to 15 aa (amino acids), while the CD4^+^ cell epitopes range from 10 to 20 aa. This is because these lengths correspond to the typical dimensions of the peptide-binding grooves of HLA molecules. In validation experiments, peptides that are excessively long or short often lead to false positives (Paul et al., 2020), so they have been excluded from this review.

**Table 1:** List of CD8^+^ T cell epitopes validated from HIV proteins.

Epitope	Protein	HXB2 start	HXB2 end	HLA	Epitope	Protein	HXB2 start	HXB2 end	HLA
GELDRWEKI	Gag	11	19	B*40:02, B*49:01	THLEGKIIL	Pol	781	789	B*15:10, B*15:17, B*39:01
KIRLRPGGK	Gag	18	26	A*03:01	HVASGYIEA	Pol	793	801	B*54:01
IRLRPGGKK	Gag	19	27	A*68:02, B*27:02, B*27:05	IEAEVIPAET	Pol	799	808	B*40:02, B*40:06
RLRPGGKKK	Gag	20	28	A*03:01	HTDNGSNF	Pol	829	836	C*05:01
RLRPGGKKKY	Gag	20	29	A*03:01, A*30:01, B*15:01, B*15:40, B*42:01	STTVKAACWW	Pol	838	847	B*57:01, B*58:01
RPGGKKHYM	Gag	22	30	B*07:02, B*42:01, B*42:02	IQQEFGIPY	Pol	850	858	B*15:01, B*15:03
RPGGKKKYML	Gag	22	31	B*07:02, B*42:01	VRDQAEHL	Pol	880	887	C*18:01
RPGGKKKYKL	Gag	22	31	B*51:01	KTAVQMAVF	Pol	888	896	B*15:24, B*35:02, B*35:03, B*53:01, B*57:01, B*57:03, B*58:01
GGKKKYKLK	Gag	24	32	B*08:01	FKRKGGIGGY	Pol	900	909	B*15:03, B*27:05
KYKLKHIVW/	Gag	28	36	A*24:02, A*68:02, A*23:01	KRKGGIGGY	Pol	901	909	B*15:03, B*27:02, B*27:05
QYKLKHIVW/					GERIVDII	Pol	912	919	B*40:02
KYRLKHIVW/					LQKQITKI	Pol	928	935	B*52:01
HYMLKHIVW					KIQNFRVYY	Pol	934	942	A*03:01, A*11:01, A*29:02, A*30:01, A*30:02, A*32:01, A*80:01
HLVWASREL^a^	Gag	33	41	C*06:02, C*08:04	YRDSRDPLW	Pol	942	950	B*38:01
LVWASRELERF^a^	Gag	34	44	B*57:03	VVPRRKAKII	Pol	974	983	B*08:01
WASRELERF^a^	Gag	36	44	B*35:01, B*42:01, B*35:03, B*53:01	VPRRKAKII	Pol	975	983	B*42:01
ELRSLYNTV^a^	Gag	74	82	B*08:01	RKAKIIRDY	Pol	978	986	B*15:01, B*15:03
RSLYNTVATLY^a^	Gag	76	86	A*02:01, A*30:02	RKAKIIRDYGK	Pol	978	988	B*08:01
SLYNTVATL^a^	Gag	77	85	A*02:01, A*02:02, A*02:05, A*02:14	KIIRDYGK	Pol	981	988	B*08:01
LYNTVATL^a^	Gag	78	85	C*14:03	IIKDYGKQM	Pol	982	990	B*42:01
LYNTVATLY^a^	Gag	78	86	A*29:02, B*44:03	RIRTWKSLVK	Vif	17	26	A*03:01
TLYCVHQK^a^	Gag	84	91	A*11:01	IRTWKSLVK	Vif	18	26	A*30:01
IEIKDTKEAL	Gag	92	101	B*35:02, B*35:03, B*40:01, B*42:01, B*53:01	IRTWKSLVKH	Vif	18	27	B*27:05
NSSKVSQNY	Gag	124	132	A*01:01, B*35:01	HMYISKKAK^a^	Vif	28	36	A*03:01, A*68:02, B*58:02
GQMVHQAI	Gag	140	147	B*13:02	ISKKAKGWF	Vif	31	39	B*35:01, B*35:02, B*35:03, B*35:08, B*53:01, B*57:01, B*58:01
HQAISPRTL	Gag	144	152	B*42:01	HPRVSSEVHI^a^	Vif	48	57	B*07:02, B*42:01
QAISPRTLNAW	Gag	145	155	A*25:01, B*35:01, B*35:08, B*58:01	WHLGHGVSI/ WHLGQGVSI	Vif	79	87	B*15:10, B*15:10, B*35:02, B*35:03, B*38:01, B*53:01
ISPRTLNAW/ LSPRTLNAW	Gag	147	155	B*35:02, B*35:03, B*53:01, B*57:01, B*57:02, B*57:03, B*58:01, C*06:02	LGHGVSIEW	Vif	81	89	B*57:03
SPRTLNAWV^a^	Gag	148	156	B*07:02 A*68:02 B*58:02	LADQLIHLHY	Vif	102	111	B*18:01
VKVIEEKAF	Gag	156	164	B*15:03	KTKPPLPSVKK	Vif	158	168	A*03:01
EEKAFSPEV	Gag	160	168	B*35:01, B*35:08, B*42:01, B*45:01	EAVRHFPRI	Vpr	29	37	A*68:02, B*35:01, B*35:02, B*35:03, B*35:08, B*51:01, B*53:01
KAFSPEVI	Gag	162	169	B*57:01, B*57:03	AVRHFPRIW	Vpr	30	38	B*57:01, B*58:01
KAFSPEVIPMF	Gag	162	172	B*48:01, B*57:01, B*57:02, B*57:03, B*58:01	VRHFPRIWL	Vpr	31	39	A*68:02, B*27:02, B*27:05, B*35:02, B*35:03, B*53:01
EVIPMFSAL	Gag	167	175	A*26:01, A*26:02, A*26:03	FPRIWLHGL	Vpr	34	42	B*07:02, B*15:10, B*42:01, B*81:01
VIPMFSAL^a^	Gag	168	175	B*15:16, C*01:02	ETYGDTWTGV	Vpr	48	57	A*68:02
SEGATPQDL	Gag	176	184	B*35:02, B*35:03, B*40:01, B*53:01	DTWAGVEAIIR	Vpr	52	62	A*68:01, A*68:02, B*35:02, B*35:03, B*53:01
TPQDLNTML/ TPQDLNMML^a^	Gag	180	188	B*07:02, B*35:02, B*35:03, B*39:10, B*42:01, B*42:02. B*53:01, B*67:01, B*81:01, C*08:02	AIIRILQQL^a^	Vpr	59	67	A*02:01
GHQAAMQML	Gag	193	201	B*15:10, B*35:02, B*35:03, B*42:01	CCFHCQVC	Tat	30	37	C*12:03
KETINEEAA^a^	Gag	202	210	B*35:02, B*35:03, B*40:02	FQTKGLGISY	Tat	38	47	B*15:01, B*15:03, B*15:40
ETINEEAAEW/ DTINEEAAEW^a^	Gag	203	212	A*25:01, A*68:01, B*35:01, B*35:08, B*53:01	ITKGLGISYGR	Tat	39	49	A*68:01, B*15:01, B*15:03, B*35:02, B*35:03, B*53:01
					EELLKTVRL^a^	Rev	10	18	B*44:02, B*44:03
AEWDRVHPV	Gag	210	218	B*35:02, B*35:03, B*40:02, B*53:01	KAVRLIKFLY/ QAVRIIKILY	Rev	14	23	B*15:03, B*35:01, B*35:02, B*35:03, B*35:08, B*53:01, B*57:01, B*58:01
HPVHAGPIA	Gag	216	224	A*68:02, B*07:02, B*35:01, B*35:02, B*35:03, B*39:10, B*53:01	ERILSTYLGR^a^	Rev	57	66	A*03:01, B*58:01
GQMREPRGSDI	Gag	226	236	B*15:40	RPAEPVPLQL	Rev	66	75	B*07:02, B*42:02
TSTLQEQIGW	Gag	240	249	A*68:02, B*57:01, B*57:03, B*58:01,	SAEPVPLQL	Rev	67	75	C*05:01
NPPIPVGDIY	Gag	253	262	B*35:01, B*35:05	YRLGVGALI	Vpu	5	13	C*18:01
PPIPVGDIY	Gag	254	262	B*35:01, B*35:02, B*53:01	EYRKILRQR	Vpu	29	37	B*35:02, B*35:03, B*53:01
EIYKRWII^a^	Gag	260	267	B*08:01, B*35:02, B*35:03, B*42:01, B*53:01	RVKEKYQHL	Env	2	10	B*08:01
RRWIQLGLQK/ KRWIILGLNK^a^	Gag	263	272	B*27:05	AENLWVTVY	Env	31	39	B*15:17, B*15:40, B*18:01, B*44:03
GLNKIVRMY^a^	Gag	269	277	B*15:01, B*35:02, B*35:03, B*53:01	AENLWVTVYY	Env	31	40	A*68:02
VRMYSPVSI	Gag	274	282	B*07:02, B*15:02, C*18:01	TVYYGVPVWK	Env	37	46	A*03:01, B*15:40, B*42:02
RMYSPTSI	Gag	275	282	B*52:01	VPVWKEATTT	Env	42	51	B*35:03, B*53:01, B*55:01
YSPVSILDI	Gag	277	285	C*01:02	VPVWKEATTTL	Env	42	52	B*35:01
FRDYVDRFF	Gag	293	301	A*01:01, B*42:02, C*18:01	KAYETEVHNVW	Env	59	69	B*58:01
FRDYVDRFYK	Gag	293	302	B*18:01	YETEVHNVW	Env	61	69	B*18:01
RDYVDRFFKTL/ RDYVDRFYKTL	Gag	294	304	A*24:02, A*68:02, B*44:02,	DPNPQEVVL	Env	78	86	B*15:02, B*35:01, B*35:02
YVDRFYKTL	Gag	296	304	A*02:07, C*03:04, A*01:01, A*26:01, B*07:02, B*15:03, B*15:10, C*03:03, C*03:04	MHEDIISLW^a^	Env	104	112	B*38:01
DRFYKTLRA	Gag	298	306	B*14:02, B*14:01	SFEPIPIHY	Env	209	217	A*29:02, B*58:01
AEQASQDVKNW	Gag	306	316	B*15:03, B*15:24, B*35:01, B*35:08, B*44:02, B*58:01	CAPAGFAIL	Env	218	226	C*01:02
AEQASQEVKNWM	Gag	306	317	B*35:01, B*35:08, B*58:01	RPNNNTRKSI	Env	298	307	B*07:02, B*42:01, B*42:02
QASQEVKNW/ QATQDVKNW/ QATQDVKNW	Gag	308	316	B*35:01, B*35:08, B*53:01, B*57:01, B*58:01	HIGPGRAFY	Env	310	318	A*30:02, B*35:02, B*35:03
					RGPGRAFVTI	Env	311	320	B*42:02
					EIIGDIRQAY	Env	321	330	A*25:01
VKNWMTETL	Gag	313	321	B*48:01	SFNCGGEFF	Env	375	383	B*15:16, C*04:01
DCKTILKAL	Gag	329	337	B*08:01, B*15:03, B*35:02, B*35:03, B*53:01	LPCRIKQII	Env	416	424	B*51:01
ACQGVGGPGHK	Gag	349	359	A*11:01, A*11:03, B*35:02, B*35:03, B*53:01	RIKQIINMW	Env	419	427	A*32:01
GPGHKARVL	Gag	355	363	B*07:02, B*42:01,	RAIEAQQHL/ RAIEAQQHM	Env	557	565	B*15:01, B*15:03, B*51:01, C*03:04, C*12:02
AEAMSQVTNS	Gag	364	373	B*40:02, B*42:01, B*45:01	QTRVLAIERYL	Env	577	587	B*58:01, B*58:02
APRKKGCWK	Gag	407	415	B*07:02	ERYLKDQQL	Env	584	592	B*08:01, B*14:01, B*14:02
TERQANFL	Gag	427	434	B*40:02	RYLKDQQLL	Env	585	593	A*23:01, A*24:02, C*07:02
RQANFLGKI	Gag	429	437	B*13:02, B*15:24, B*48:01	YLKDQQLL	Env	586	593	A*24:02, B*08:01
FLGKIWPSYK	Gag	433	442	A*02:01, A*02:05	TAVPWNASW	Env	606	614	B*35:01, B*35:08, B*58:01
KELYPLTSL/ KELYPLASL	Gag	481	489	B*35:02, B*35:03, B*40:01, B*53:01, B*40:02	IVNRNRQGY	Env	704	712	A*30:02
NSPTRREL	Pol	24	31	C*01:02	IVTRIVELL	Env	777	785	A*02:05, B*15:17
ITLWQRPLV	Pol	59	67	A*68:02, A*74:01, B*35:02, B*35:03, B*53:01	GRRGWEALKY	Env	786	795	B*15:40, B*27:05, B*35:02, B*35:03, B*53:01
DTVLEEWNL/DTVLEEMNL	Pol	86	94	A*68:02, B*35:02, B*35:03, B*53:01	RRGWEVLKY	Env	787	795	A*01:01
EEMNLPGRW	Pol	90	98	A*68:02, B*35:02, B*35:03, B*44:02, B*44:03	KYCWNLLQY	Env	794	802	A*30:02
RQYDQILIEI^a^	Pol	113	122	B*13:02	QELKNSAVSL	Env	805	814	B*08:01, B*35:02, B*35:03, B*40:01, B*53:01
GKKAIGTVL	Pol	124	132	B*15:03	SLLNATDIAV	Env	813	822	A*02:01
KAIGTVLV	Pol	126	133	B*35:01, B*35:08	LLNATDIAV	Env	814	822	A*02:01, A*02:05
LVGPTPVNI	Pol	132	140	A*02:01	IPRRIRQGL	Env	843	851	B*07:02, B*42:01
TPVNIIGRNML	Pol	136	146	B*81:01	RIRQGLERA	Env	846	854	A*02:05
FPISPIETV^a^	Pol	155	163	B*54:01	RQGLERALL	Env	848	856	B*81:01
IETVPVKL	Pol	160	167	B*15:02, B*35:02, B*35:03, B*40:01, B*53:01	WPTVRERM	Nef	13	20	B*08:01, B*15:16
GPKVKQWPL	Pol	173	181	B*08:01, B*42:02	RMRRAEPAA	Nef	19	27	B*15:01, B*58:01
ALVEICTEM	Pol	188	196	A*02:01	LEKHGAITS	Nef	37	45	B*40:01
TVLDVGDAY	Pol	262	270	B*15:02, B*35:01, B*35:02, B*35:03, B*53:01, B*57:01	FPVTPQVPL	Nef	68	76	A*68:02, B*07:02
SVPLDEGFRK	Pol	272	281	A*11:01	FPVTPQVPLR	Nef	68	77	A*68:02, B*07:02
VPLDEDFRKY	Pol	273	282	B*15:02, B*35:01, B*35:02, B*35:03, B*42:01, B*53:01	TPQVPLRPM/ RPQVPLRPM	Nef	71	79	A*68:02, B*07:02, B*07:02, B*35:01, B*35:02, B*35:03, B*42:01, B*42:02, B*53:01, B*67:01, B*81:01
YTAFTIPSI	Pol	282	290	A*02:01, A*02:05, B*35:02, B*35:03, B*53:01	RPQVPLRPMTY	Nef	71	81	B*35:01, B*67:01, C*07:02
TAFTIPSI	Pol	283	290	A*02:01, A*02:17, B*35:02, B*35:03, B*51:01, B*52:01, B*53:01	QVPLRPMTYK	Nef	73	82	A*02:01, A*03:01, A*11:01, B*35:02, B*35:03, B*53:01
IRYQYNVL	Pol	297	304	B*14:01	VPLRPMTY	Nef	74	81	B*35:01, B*35:02, B*35:03, B*53:01
IRYQYNVLP	Pol	297	305	B*73:01	PLRPMTYK	Nef	75	82	A*11:01, B*15:01
LPQGWKGSPA	Pol	304	313	B*54:01	LRPMTYKAA	Nef	76	84	B*27:03
SPAIFQSSM	Pol	311	319	A*68:02, B*07:02, B*15:17, B*35:01, B*42:01, B*42:02, B*81:01	RPMTYKAAL	Nef	77	85	B*07:02, B*67:01, B*42:01, B*42:02
SPAIFQSSMTK	Pol	311	321	A*11:01, A*68:01 B*07:02	KAAFDLSFF	Nef	82	90	B*57:01, B*57:02, B*57:03, B*58:01, C*12:02
AIFQSSMTK	Pol	313	321	A*03:01, A*11:01, A*68:01	KAAVDLSHFL^a^	Nef	82	91	C*08:04
KQNPDIVIY	Pol	328	336	A*30:02, B*15:01, B*15:03, B*35:02, B*35:03, B*53:01, C*12:02	GAFDLSFFL/ AAVDLSHFL/ AAFDLSFFL/ AALDLSHFL^a^	Nef	83	91	A*02:05, A*02:06, B*15:17, B*14:02, B*42:02, B*35:02, B*35:03, B*53:01, B*57:03, C*06:02
NPEIVIYQY/ HPDIVIYQY	Pol	330	338	B*35:01, B*35:02, B*35:03, B*35:08, B*42:01, B*53:01,	AVDLSHFLK^a^	Nef	84	92	A*03:01, A*11:01, B*15:03, B*15:17
EIVIYQYMD	Pol	332	340	B*18:01	FLKEKGGL	Nef	90	97	B*08:01, B*35:01, B*35:08
VIYQYMDDL	Pol	334	342	A*02:01, B*35:02, B*35:03, B*53:01	KEKGGLEGL	Nef	92	100	B*35:02, B*35:03, B*40:01, B*40:02, B*44:03, B*53:01
VIYQYMDDLYV	Pol	334	344	A*02:01	KEKGGLEGLIY	Nef	92	102	B*44:03
IEELRQHLL	Pol	357	365	B*40:01	RRQDILDLWI^a^	Nef	105	114	B*27:05
IVLPEKDSW	Pol	399	407	B*35:01, B*35:02, B*35:03, B*35:08, B*53:01, B*57:01, B*57:02, B*57:03, B*58:01	RRQDILDLWVY/KRQEILDLWVY	Nef	105	115	B*18:01, B*58:01, C*07:01, C*07:02, B*35:02, B*35:03
LVGKLNWASQIY	Pol	415	426	B*15:01, B*15:03	RQDILDLWI	Nef	106	114	B*13:02, B*15:24, B*13:02
KLNWASQIY	Pol	418	426	A*30:02, B*15:01, B*15:02, B*35:02, B*35:03, B*53:01	QEILDLWVY	Nef	107	115	B*18:01, B*44:02, B*44:03
QIYPGIKVR	Pol	424	432	A*03:01, A*74:01	HTQGYFPDW	Nef	116	124	B*15:03, B*57:01, B*57:03, B*58:01
YPGIKVRQL	Pol	426	434	B*42:01, B*42:02, B*58:01	TQGYFPDWQNY	Nef	117	127	B*15:01
IPLTEEAEL	Pol	448	456	B*07:02, B*15:01, B*35:01, B*51:01, B*53:01	GYFPDWQNY	Nef	119	127	B*58:01
ILKEPVHGV	Pol	464	472	A*02:01, A*02:02, A*02:05	YFPDWQNYT	Nef	120	128	B*35:01, B*37:01, B*57:01, B*58:01
ILKEPVHGVY	Pol	464	473	B*15:01, B*15:10, C*03:03, C*12:02	FPDWQNYTP	Nef	121	129	B*54:01
IYQEPFKNLK	Pol	496	505	A*11:01	NYTPGPGIRY	Nef	126	135	A*24:02
RMRGAHTNDV	Pol	511	520	A*30:02, B*35:02, B*35:03, B*53:01	YTPGPGIRY	Nef	127	135	B*35:02, B*35:03, B*53:01
IAMESIVIW	Pol	530	538	B*35:01, B*35:08, B*57:02, B*57:03, B*58:01	TPGPGVRYPL	Nef	128	137	B*07:02, B*35:01, B*35:02, B*35:03, B*42:01, B*42:02, B*53:01, B*81:01
PIQKETWETW	Pol	547	556	A*32:01, B*15:17, B*35:01, B*35:08	TRYPLTFGW	Nef	133	141	A*68:02, B*35:01, B*35:02, B*35:03, B*35:08, B*42:01, B*53:01
GAETFYVDGA	Pol	591	600	A*68:02	RYPLTFGW	Nef	134	141	A*23:01, A*24:02, B*35:02, B*35:03, B*53:01
ETFYVDGA	Pol	593	600	A*68:02	RYPLTFGWCY	Nef	134	143	A*24:02, C*07:02, A*23:01
ETFYVDGAANR	Pol	593	603	A*68:01, A*68:02, B*15:16	YPLTFGWCY/ YPLTFGWCF	Nef	135	143	B*07:02, B*18:01, B*35:01, B*35:02, B*35:03, B*35:08, B*53:01, B*58:01, B*67:01
ETKLGKAGY	Pol	604	612	A*26:01, B*15:01	PLTFGWCYKL	Nef	136	145	A*02:01, B*35:02, B*35:03, B*53:01, B*58:01
VTDSQYALGI	Pol	651	660	B*14:02, B*15:03, B*35:02, B*35:03, B*53:01	LTFGWCFKL	Nef	137	145	A*02:01, A*68:02, B*15:17, B*15:40
QIIEQLIKK	Pol	675	683	A*11:01	VLEWRFDSRL	Nef	180	189	A*02:01
LFLDGIDKA	Pol	715	723	B*15:01	WRFDSRLAF	Nef	183	191	B*15:03, B*27:05
LPPIVAKEI	Pol	743	751	B*07:05, B*42:01, B*51:01					

Note:

a stand for those peptides are located in surface exposed loops of the experimental structures or Alpha Fold models of virus proteome.

**Table 2:** List of CD4^+^ cell epitopes validated from HIV proteins.

Epitope	Protein	HXB2 start	HXB2 end	HLA	Epitope	Protein	HXB2 start	HXB2 end	HLA
SGGELDRWEKIRLRPGGK	Gag	9	26	DRB1*07:01, DRB1*11:01, DRB1*13:01, DRB1*13:02, DRB3*01:01	RFYKTLRAEQAS	Gag	299	310	DRB1*01:01, DRB1*04:01, DRB1*04:05, DRB1*07:01, DRB1*11:01, DRB1*15:01, DRB5*01:01
LRPGGKKKYKLKHIV	Gag	21	35	DRB1*13:02	RFYKTLRAEQASQEVK	Gag	299	314	DRB1*01:01
GKKKYKLKHIVWASREL	Gag	25	41	DRB1*01:01, DRB1*07:01, DRB1*08:01, DRB1*11:01, DRB1*13:02, DRB1*13:03, DRB3*01:01, DRB3*03:01, DRB4*01:01, DRB5*01:01	FYKTLRAEQASQ	Gag	300	311	DRB1*01:01, DRB1*04:01, DRB1*11:01, DRB5*01:01
KHIVWASRELERFAV	Gag	32	46	DRB1*03:01, DRB1*13:01, DRB1*13:02, DRB1*13:03, DRB3*01:01, DRB5*01:01	FYKTLRAEQASQE	Gag	300	312	DRB1*01:01, DRB1*04:01, DRB1*04:05, DRB1*11:01, DRB1*15:01, DRB5*01:01
ASRELERFAVNPGLL	Gag	37	51	DRB1*01:01, DRB1*04:01, DRB1*04:05, DRB1*07:01, DRB1*13:01, DRB1*13:02, DRB1*15:01, DRB4*01:01	YKTLRAEQASQEVKN	Gag	301	315	DRB1*01:01
LERFAVNPGLL	Gag	41	51	DRB1*13:02	RAEQASQEVKNWMTE	Gag	305	319	DRB1*01:01
LERFAVNPGLLETSE	Gag	41	55	DRB1*01:01	EVKNWMTETLLVQNA	Gag	312	326	DRB1*14:01
ERFAVNPGLLETSEGCR	Gag	42	58	DRB1*01:01, DRB1*04:05, DRB1*11:01, DRB1*13:02, DRB1*13:03, DRB1*15:01, DRB3*03:01	TNSATIMMQRGNFRNQRK	Gag	371	388	DRB1*15:02
TGSEELRSLYNTVATLY	Gag	70	86	DRB1*04:05, DRB1*07:01	CFNCGKEGHIAKNCRAPR	Gag	392	409	DRB1*09:01, DRB1*13:01
EELRSLYNTVATLYC^a^	Gag	73	87	DRB1*01:01	HIAKNCRAPRKKGCWK	Gag	400	415	DRB1*07:01, DRB1*13:01
EELRSLYNTVATLYCVH^a^	Gag	73	89	DRB1*01:01, DRB1*04:01, DRB1*04:05, DRB1*07:01, DRB1*11:01, DRB1*13:02, DRB1*15:01	RAPRKKGCWKCGKEGHQM	Gag	406	423	DRB1*09:01
SLYNTVATLYCVHQR^a^	Gag	77	91	DRB1*01:01	RQANFLGKIWPSHKGR	Gag	429	444	DRB1*01:01, DRB1*04:01, DRB1*04:05, DRB1*11:01, DRB1*13:01, DRB1*13:02, DRB1*15:01, DRB5*01:01
SLYNTVATLYCVHQRIEV	Gag	77	94	DRB1*01:01, DRB1*04:01, DRB1*04:05, DRB1*07:01, DRB1*13:02, DRB1*14:01, DRB5*01:01	GKIWPSHKGRPGNFLQSR	Gag	435	452	DRB1*07:01, DRB1*13:01, DRB1*15:01
PIVQNIQGQ	Gag	133	150	DRB1*01:01	FLQSRPEPTAPPEESFRF	Gag	448	465	DRB1*07:01
IVQNLQGQMVHQAISPR	Gag	134	150	DRB1*09:01	DKELYPLASLRSLFG	Gag	480	494	DRB1*01:01, DRB1*04:01, DRB1*04:05, DRB1*07:01, DRB1*11:01, DRB1*15:01, DRB5*01:01
PRTLNAWVKVVEEKAF	Gag	149	164	DRB1*13:01, DRB1*13:04	QRPLVTIKIGGQLKE	Pol	63	77	DRB1*01:01, DRB1*11:01, DRB1*15:01, DRB5*01:01
WVKVVEEKAFSPEVIPMF	Gag	155	172	DRB1*11:01	TPVNIIGRNLLTQIG	Pol	136	150	DRB1*01:01, DRB1*11:01, DRB1*13:02, DRB1*15:01
AFSPEVIPMFSALSEGA	Gag	163	179	DRB1*04:01, DRB1*07:01, DRB1*15:01, DRB4*01:01	VIWGKTPKFKLPIQKETW^a^	Pol	536	553	DRB1*13:01, DRB3*01:01
SPEVIPMFSALSE	Gag	165	177	DRB1*01:01, DRB1*03:01, DRB1*04:01, DRB1*04:05, DRB1*11:01, DRB1*15:01	GKIILVAVHVASGYI	Pol	785	799	DRB1*01:01, DRB1*04:01, DRB1*04:05, DRB1*07:01, DRB1*11:01, DRB1*13:02, DRB1*15:01, DRB5*01:01
ETINEEAAEWDRVHPVHA	Gag	203	220	DRB1*01:01	SLQYLALVALVAPKK	Vif	144	158	DRB1*01:01, DRB1*04:01, DRB1*04:05, DRB1*07:01, DRB1*11:01, DRB1*15:01, DRB5*01:01
AAEWDRLHPVHAGPIA	Gag	209	224	DRB1*07:01	EAIIRILQQLLFIHF	Vpr	58	72	DRB1*01:01, DRB1*04:05, DRB1*15:01
LHPVHAGPIAPGQMREPR	Gag	215	232	DRB1*11:01	QQLLFIHFRIGCRHSRIG^a^	Vpr	65	82	DRB1*01:01, DRB1*04:05, DRB1*07:01, DRB1*11:01, DRB1*13:02, DRB1*15:01, DRB5*01:01
GSDIAGTTSTLQEQI	Gag	233	247	DRB1*01:01	TKGLGISYGRKKRRQRRR	Tat	40	57	DRB1*13:02, DRB1*13:03, DRB3*01:01, DRB3*03:01
SDIAGTTSTLQEQIGWM	Gag	234	250	DRB1*04:04, DRB1*15:02	ELLKTVRLIKFLYQSNP	Rev	11	27	DRB1*01:01, DRB1*03:01, DRB1*04:01, DRB1*04:05, DRB1*07:01, DRB1*11:01, DRB1*13:02, DRB1*15:01
STLQEQIGWMTNNPP	Gag	241	255	DRB1*01:01	PSPEGTRQARRNRRRRW	Rev	29	45	DRB1*13:01, DRB3*01:01
STLQEQIGWMTNNPPIPV	Gag	241	258	DRB1*01:01, DRB1*13:03, DRB3*01:01, DRB3*03:01	TMLLGMLMICSAA	Env	19	31	DRB1*01:01, DRB1*04:05, DRB1*11:01
WMTNNPPIPVGEIYK	Gag	249	263	DRB1*01:01	AAEQLWTVYYGVPPVW	Env	30	45	DRB1*11:01, DRB1*15:01
WMTNNPPIPVGEIYKRWI	Gag	249	266	DRB3*01:01, DRB3*03:03	NVTENFNMWKNNMVEQMH	Env	88	105	DRB1*11:01, DRB1*15:01, DRB1*15:02
TNNPPIPBGEIYKRW	Gag	251	265	DRB1*13:01	NFNMWKNNMVEQM	Env	92	104	DRB1*15:02
NPPIPVGEIYKRWII	Gag	253	267	DRB1*01:01	DKMQKEYALLYKLDI	Env	167	181	DRB1*03:01, DRB1*12:01, DRB1*15:01, DRB4*01:01, DRB5*01:01
PVGEIYKRWIILGLN^a^	Gag	257	271	DRB1*01:01	DKKQKVHALFYKLDIV	Env	167	182	DRB1*12:02
IYKRWIILGLNKIVR^a^	Gag	261	275	DRB1*01:01	NTSYRLISCNTSVI	Env	188	201	DRB1*01:01, DRB1*04:01, DRB1*04:05, DRB1*07:01, DRB1*11:01, DRB1*13:02, DRB1*15:01, DRB5*01:01
WIILGLNKIVRM^a^	Gag	265	276	DRB1*01:01, DRB1*04:01, DRB1*04:05, DRB1*11:01, DRB1*13:02, DRB1*15:01, DRB5*01:01	KVSFEPIPIHYCAPAGFA	Env	207	224	DRB1*01:01, DRB1*11:01, DRB3*01:01
WIILGLNKIVRMYSP	Gag	265	279	DQB1*06:02, DQB1*06:04, DRB1*13:02, DRB1*15:01, DRB3*03:01, DRB5*01:01,	SELYLYKVVKIEPLGVAP	Env	481	498	DRB1*01:01, DRB1*04:01, DRB1*04:05, DRB1*07:01, DRB1*11:01, DRB1*13:02, DRB1*15:01, DRB5*01:01
VIPMFSAL	Gag	168	175	DQB1*06:01, DRB1*13:03, DRB1*15:02, DRB5*01:02	RDLLLIVTRIVELLGR	Env	772	787	DRB1*01:01, DRB1*07:01, DRB1*11:01
WIILGLNKIVRMYSPVSI	Gag	265	282	DRB1*01:01, DRB1*03:02, DRB1*04:01, DRB1*04:05, DRB1*07:01, DRB1*11:01, DRB1*13:01, DRB1*13:02, DRB1*15:01, DRB1*15:02, DRB5*01:01	RSVVGWPAVRERMRRA	Nef	8	23	DRB1*13:02
GLNKIVRMYSPTSIL	Gag	269	283	DRB1*01:01	YKAAVDLSHFLKEKGGL	Nef	81	97	DRB1*07:01, DRB1*08:04
GPKEPFRDYVDRFYKTLR	Gag	288	305	DRB1*13:01	LWVYHTQGYFPDWQNY	Nef	112	127	DRB1*07:01, DRB1*13:01, DRB1*14:01, DRB3*01:01, DRB4*01:01
DYVDRFYKTLRAE	Gag	295	307	DRB1*01:01	PEKEVLVWKFDSRLAFHH	Nef	176	193	DRB1*01:01, DRB1*04:01, DRB1*07:01, DRB1*11:01, DRB1*13:02, DRB1*15:01, DRB5*01:01
YVDRFYKTLRAEQASQEV	Gag	296	313	DRB1*01:01, DRB1*04:01, DRB1*04:05, DRB1*07:01, DRB1*08:01, DRB1*10:01, DRB1*11:01, DRB1*13:01	VLEWRFDSRLAFHHV	Nef	180	194	DRB1*01:01, DRB1*07:01, DRB1*11:01, DRB1*15:01, DRB5*01:01
VDRFYKTLRAEQASQ	Gag	297	311	DRB1*13:02, DRB1*13:03, DRB1*15:01, DRB1*15:02, DRB4*01:01, DRB5*01:01 DRB1*01:01	KFDSRLAFHHMARELH	Nef	184	199	DRB1*01:01, DRB1*03:02, DRB1*07:01, DRB1*11:01, DRB1*13:02, DRB1*13:03, DRB1*15:01, DRB3*03:01, DRB5*01:01
DRFYKTLRAEQASQ	Gag	298	311	DRB1*04:01					

Note:

a stand for those peptides are located in surface exposed loops of the experimental structures or Alpha Fold models of virus proteome.

**Fig. 1:**
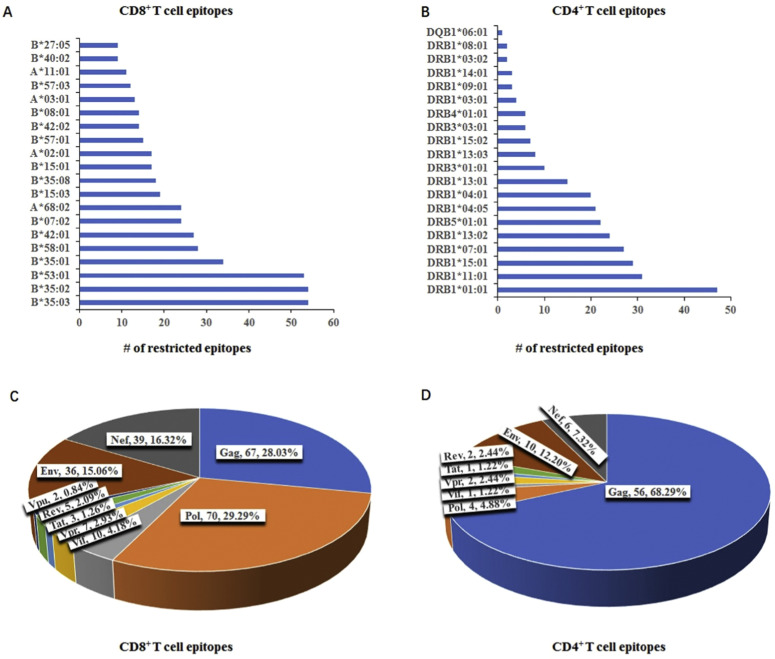
HLA restriction and protein distribution of validated CD8^+^ T cell epitopes and CD4^+^ T cell epitopes in HIV proteome. (A, B) displayed the number of CD8^+^ T cell epitopes and CD4^+^ T cell epitopes restricted by each HLA supertype, respectively. (C, D) showed the fraction of CD8^+^ T cell epitopes and CD4^+^ T cell epitopes in each HIV protein, respectively.

In brief, 270 studies documented the validated epitopes presented by class I molecules, while 27 studies reported those presented by class II molecules. In total, these studies encompassed 321 unique epitopes, comprising 239 and 82 T cell epitopes for CD8^+^ T cell and CD4+ T cell (Table 1, Table 2). Among them, the majority of epitopes for CD8+ T cell are presented by HLA-B35, B5301, B5801, or B4201 (Fig. 1A). These are prevalent alleles in Caucasian populations and are less common in Asia and Africa (Barker et al., 2023). The remaining epitopes are restricted primarily by 25 HLA-A, 50 HLA-B, and 14 HLA-C alleles. In terms of epitopes for CD4^+^T cell, the majority apply to 8 DRB1, DRB5*01:01, and DRB3*01:01 (Fig. 1B). The combined frequency of the HLA-A supertypes was highest in Europe at 49.34 %, followed by North America at 39.03 %, Asia at 28.81 %, and Africa at 26.91 % (Fig. 1A). In contrast, the HLA-B supertypes exhibited an aggregate frequency of 49.67 % in Europe, 43.51 % in North America, 40.56 % in Africa, and 28.84 % in Asia. The DRB1 supertypes shown in Fig. 1B exhibited minimal variation in their cumulative gene frequency across Europe (82.52 %), North America (69.11 %), Africa (63.49 %), and Asia (59.77 %). (http://www.allelefrequencies.net/).

Furthermore, 88.7 % of the epitopes for CD8+ T cell belong to the Pol, Gag, Nef, and Env protein (Fig. 1C), while 87.81 % of epitopes for CD4+ T cell are from the Gag, Nef and Env protein (Fig. 1D). The biased distribution of epitopes is partially due to the lengths of proteins (Env 857aa, Gag 503aa, Pol 1008aa, and so on) and the sequence conservation. Notably, our review only includes epitopes with the four digits of explicitly HLA type. HLA typing that is specific to the last four digits can further refine the polymorphic information of HLA molecules, allowing for a more accurate understanding of the binding affinity between an individual’s HLA molecules and antigen peptides. In contrast, HLA typing that only specifies the first two digits may cover multiple molecules with different binding properties.

To inform epitope prediction, we attempted to analyze sequence or structural characteristics of 321 viral peptides. The presence of glycine may indicate high structural flexibility in this region, which is conductive to recognition by the immune system. 33 of 239 CD8+ T-cell epitope peptides and 15 of 82 CD4+ T-cell epitope peptides contain one, two or three glycine residues. 37 of 321 located in surface exposed loops of the experimental structures or Alpha Fold models of virus proteome. During antigen presentation, different HLA molecules bind to different antigenic peptides. Most 9 peptides with high or moderate binding affinity show anchor residue at the p2 and COOH terminal or p9. High-affinity peptides restricted by HLA-A02 mostly with x-**L/V/M**-x-x-x-x-x-x-**L/I/V** motif, A2402 with x -**Y/F/M**-x-x-x-x-x-x-**F/L/I**, HLA-A1101 with x-**S/A/V**-x-x-x-x-x-x-**K/R**, and HLA-A3303 with x-**L/S/A**-x-x-x-x-x-x-**K/R** in our previous research (Ding et al., 2025). In this paper, the pattern motifs of the top three epitopes restricted by HLA-B3503 or HLA-B3502 mostly with x-**E/T/P**-x-x-x-x-x-x-**L/Y/I** motif and HLA-B5301 with x-**T/E/P**-x-x-x-x-x-x-**L/Y/W**. In addition, every epitope’s sites of interest were analyzed according reported, which included drug resistance, spatial structure and so on in Table S1, TableS2.

At present, according to the statistics from the HIV Molecular Immunology Database (https://www.HIV.lanl.gov/content/immunology/), 2048 different entries of CTL epitopes and 702 CD4^+^ epitopes are listed along with associated HLA information. The reported CD8+ and CD4+ T cell epitopes screened by the *in silico* prediction strategy are about 88.33 % and 88.32 % of epitopes, respectively. Of these only 139 HIV-1 epitope peptides presented by HLA-C alleles have been screened. Epitopes from the Rev, Vif, Vpr, Vpu, and Tat proteins are also very rare. Antigenic peptides of HIV-1 subtypes A/B/C/D/CRF01_AE account for 11.43 %, 58.26 %, 21.69 %, 4.96 %, and 3.65 %, respectively. Evidently, the 321 validated T cell epitopes of HIV are insufficient to fully encompass the primary populations and HIV subtypes in a designated geographic zone. More efforts are urgently needed to concentrate on regional differences, especially regarding the HIV subtypes and populations in Asia, in order to design therapeutic vaccines and the detection system of HIV-specific T-cells.

## 6 Prospect and conclusion

As interest in T cell-based HIV cure strategies resurges, careful consideration of HIV-specific epitopes is crucial. Our research gathers T cell epitopes defined by the HIV proteome and demonstrates their HLA restriction with four digits and methods for validating their immunogenicity. These epitope catalogs provide strong support for researching therapeutic vaccines, specific T-cell detection, and the interaction mechanism between HIV and the immune system. Nevertheless, 321 T cell epitopes that have been identified thus far do not completely encompass the predominant populations and HIV subtypes found in certain geographical areas. To address this, we propose a three-tiered strategy: (1) establish global collaborative networks to prioritize sampling in Asia, Africa, and South America, where HIV epidemics were characterized by unique clade distributions; (2) implement longitudinal cohort studies combining high-throughput epitope-HLA sequencing with clinical data on viral load and treatment response; (3) employ single-cell RNA sequencing to map rare epitope-specific T-cell clones and track their dynamic evolution during infection. These efforts will enable the design of pan-subtype vaccines and precision immune monitoring tools, ultimately advancing T cell-based HIV cure strategies.

## Consent for publication

Not applicable.

## Ethics approval and consent to participate

Not applicable.

## Funding

This work was supported in part by the Talent Support Program of the Second Hospital of Nanjing (RCZD24002); the Nanjing Medical Science and Technique Development Foundation (YKK24180); Laboratory Department, Key Medical Specialty of Nanjing City; Anhui Provincial Key Natural Science Research Project for Universities (KJ2020A0853); School Enterprise Cooperation grant of Quality Engineering Project of Department of Education of Anhui Province (2020sjjd067); and Human Anatomy Teaching Team grant of Anhui Quality Engineering Project of Department of Education of Anhui Province (2021jxtd141); The sponsors had no role in study design, data collection and analysis, preparation of the manuscript, or decision to submit the article for publication.

## CRediT authorship contribution statement

**Yan Ding:** Methodology, Investigation. **Ling Huang:** Investigation, Data curation. **Yandan Wu:** Writing – original draft, Methodology. **Jialai Yan:** Writing – review & editing, Funding acquisition.

## Declaration of competing interest

The authors declare that they have no known competing financial interests or personal relationships that could have appeared to influence the work reported in this paper.

## Data Availability

Data will be made available on request.

## Appendix Supplementary materials

Image, application 1
