# Supplementary material for: A systematic review of T cell epitopes defined from the proteome of human immunodeficiency virus
Source: Virus Res. 2025 Jun 23;358:199602. doi: 10.1016/j.virusres.2025.199602 (PMC12269980; doi:10.1016/j.virusres.2025.199602)
Supplement: Supplementary file 1 [file mmc1.docx]

A Systematic Review of T Cell Epitopes Defined from the Proteome of Human Immunodeficiency Virus

Yan Ding**^1^**, Ling Huang**^1^**, Yandan Wu**^2*^**, Jialai Yan**^3*^**

**Table S1. List of CD8^+^ T cell epitopes validated from HIV proteins.**

| **Epitope** | **Protein** | **HXB2 start** | **HXB2 end** | **Sites of interest** | **HLA** | **Reference** |
| --- | --- | --- | --- | --- | --- | --- |
| GELDRWEKI^1, 2^ | Gag/p17 | 11 | 19 | binds plasma membrane  E12K drug resistance associated | B*40:02, B*49:01 | (1, 2) |
| KIRLRPGGK^1, 2, 3, 4^ | Gag/p17 | 18 | 26 | binds plasma membrane | A*03:01 | \| (3-6) \| \| --- \| |
| IRLRPGGKK^1, 3, 4, 5^ | Gag/p17 | 19 | 27 | binds plasma membrane | A*68:02, B*27:02, B*27:05 | (2, 7, 8) |
| RLRPGGKKK^1, 2, 3, 4^ | Gag/p17 | 20 | 28 | binds plasma membrane | A*03:01 | (3-6) |
| RLRPGGKKKY^1, 3, 4, 5^ | Gag/p17 | 20 | 29 | binds plasma membrane | A*03:01, A*30:01, B*15:01, B*15:40, B*42:01 | (9-13) |
| RPGGKKHYM^1, 4, 5^ | Gag/p17 | 22 | 30 | binds plasma membrane | B*07:02, B*42:01, B*42:02 | (14, 15) |
| RPGGKKKYML^1, 3^ | Gag/p17 | 22 | 31 | binds plasma membrane | B*07:02, B*42:01 | (16) |
| RPGGKKKYKL^1, 3^ | Gag/p17 | 22 | 31 | binds plasma membrane | B*51:01 | (17) |
| GGKKKYKLK^1, 3^ | Gag/p17 | 24 | 32 | binds plasma membrane | B*08:01 | \| (12, 18) \| \| --- \| |
| KYKLKHIVW/  QYKLKHIVW/  KYRLKHIVW/  HYMLKHIVW^1, 3, 4^ | Gag/p17 | 28 | 36 | binds plasma membrane  V35I drug resistance associated | A*24:02, A*68:02, A*23:01 | \| (9, 12, 19-23) \| \| --- \| |
| HLVWASREL^1, 2, 3^ | Gag/p17 | 33 | 41 | V35I drug resistance associated  E40K drug resistance associated | C*06:02, C*08:04 | \| (24, 25) \| \| --- \| |
| LVWASRELERF^1, 3, 4, 5^ | Gag/p17 | 34 | 44 | V35I drug resistance associated  E40K drug resistance associated | B*57:03 | (26) |
| WASRELERF^1, 3, 5^ | Gag/p17 | 36 | 44 | E40K drug resistance associated | B*35:01, B*42:01, B*35:03, B*53:01 | \| (12, 27-29) \| \| --- \| |
| ELRSLYNTV^1^ | Gag/p17 | 74 | 82 | L75R drug resistance associated | B*08:01 | (2) |
| RSLYNTVATLY^1, 2, 3, 4^ | Gag/p17 | 76 | 86 | / | A*02:01, A*30:02 | \| (1, 10, 19, 20, 30-33) \| \| --- \| |
| SLYNTVATL^1, 2, 3, 4, 5^ | Gag/p17 | 77 | 85 | / | A*02:01, A*02:02, A*02:05, A*02:14 | \| (6, 34-46) \| \| --- \| |
| LYNTVATL^5^ | Gag/p17 | 78 | 85 | / | C*14:03 | (47) |
| LYNTVATLY^1, 2, 3, 4^ | Gag/p17 | 78 | 86 | / | A*29:02, B*44:03 | \| (19, 25, 27, 48) \| \| --- \| |
| TLYCVHQK^1, 3^ | Gag/p17 | 84 | 91 | / | A*11:01 | \| (3, 27) \| \| --- \| |
| IEIKDTKEAL^1, 3^ | Gag/p17 | 92 | 101 | / | B*35:02, B*35:03, B*40:01, B*42:01, B*53:01 | \| (2, 12, 27, 49) \| \| --- \| |
| NSSKVSQNY^1, 3, 4, 5^ | Gag/p24 | 124 | 132 | / | A*01:01, B*35:01 | \| (27, 28, 50) \| \| --- \| |
| GQMVHQAI^1^ | Gag/p24 | 140 | 147 | / | B*13:02 | (51) |
| HQAISPRTL^1, 3^ | Gag/p24 | 144 | 152 | / | B*42:01 | (12) |
| QAISPRTLNAW^1, 3^ | Gag/p24 | 145 | 155 | / | A*25:01, B*35:01, B*35:08, B*58:01 | \| (2, 12, 52-54) \| \| --- \| |
| ISPRTLNAW/ LSPRTLNAW^1, 2, 3, 4, 5^ | Gag/p24 | 147 | 155 | / | B*35:02, B*35:03, B*53:01, B*57:01, B*57:02, B*57:03, B*58:01, C*06:02 | \| (19, 26, 43, 55-63) \| \| --- \| |
| SPRTLNAWV^1, 2, 3, 4, 5^ | Gag/p24 | 148 | 156 | / | B*07:02, A*68:02, B*58:02 | \| (12, 14, 50, 64-66) \| \| --- \| |
| VKVIEEKAF^1, 2, 3, 4, 5^ | Gag/p24 | 156 | 164 | / | B*15:03 | \| (10, 12, 15, 19, 20, 48, 67-70) \| \| --- \| |
| EEKAFSPEV^1, 3^ | Gag/p24 | 160 | 168 | / | B*35:01, B*35:08, B*42:01, B*45:01 | \| (12, 20, 70) \| \| --- \| |
| KAFSPEVI^1, 3^ | Gag/p24 | 162 | 169 | / | B*57:01, B*57:03 | (71) |
| KAFSPEVIPMF^1, 2, 3, 4, 5^ | Gag/p24 | 162 | 172 | / | B*48:01, B*57:01, B*57:02, B*57:03, B*58:01 | \| (1, 2, 10, 18, 19, 26, 39, 43, 67, 71-88) \| \| --- \| |
| EVIPMFSAL^1, 2, 3, 5^ | Gag/p24 | 167 | 175 | / | A*26:01, A*26:02, A*26:03 | (10, 19, 22, 76, 89-92) |
| VIPMFSAL^1, 3, 5^ | Gag/p24 | 168 | 175 | / | B*15:16, C*01:02 | \| (12, 52-54, 93-95) \| \| --- \| |
| SEGATPQDL^1, 3^ | Gag/p24 | 176 | 184 | / | B*35:02, B*35:03, B*40:01, B*53:01 | (12, 27) |
| TPQDLNTML/ TPQDLNMML^1, 2, 3, 4, 5^ | Gag/p24 | 180 | 188 | / | B*07:02, B*35:02, B*35:03, B*39:10, B*42:01, B*42:02. B*53:01, B*67:01, B*81:01, C*08:02 | (1, 9, 10, 12, 14, 16, 21, 50, 96, 97) |
| GHQAAMQML^1, 3^ | Gag/p24 | 193 | 201 | Q199H drug resistance associated | B*15:10, B*35:02, B*35:03, B*42:01 | (10, 12, 69) |
| KETINEEAA^1, 2, 3^ | Gag/p24 | 202 | 210 | / | B*35:02, B*35:03, B*40:02 | (1, 12) |
| ETINEEAAEW/  DTINEEAAEW^1, 3, 4^ | Gag/p24 | 203 | 212 | / | A*25:01, A*68:01, B*35:01, B*35:08, B*53:01 | (1, 2, 52-54, 98) |
| AEWDRVHPV^1, 2, 3^ | Gag/p24 | 210 | 218 | / | B*35:02, B*35:03, B*40:02, B*53:01 | (1, 12, 24, 99) |
| HPVHAGPIA^1, 2, 3, 4, 5^ | Gag/p24 | 216 | 224 | H219Q drug resistance associated  Pro217-Pro225 is Cyclophilin A binding site | A*68:02, B*07:02, B*35:01, B*35:02, B*35:03, B*39:10, B*53:01 | (10, 12, 14, 27, 28) |
| GQMREPRGSDI^1, 3^ | Gag/p24 | 226 | 236 | / | B*15:40 | (12) |
| TSTLQEQIGW^1, 2, 3, 4, 5^ | Gag/p24 | 240 | 249 | / | A*68:02, B*57:01, B*57:03, B*58:01 | (2, 10, 12, 13, 18-20, 22, 26, 39, 57-60, 62, 67, 71, 72, 77, 79, 81-86, 100-112) |
| NPPIPVGDIY^1, 2, 3, 4, 5^ | Gag/p24 | 253 | 262 | / | B*35:01, B*35:05 | (113, 114) |
| PPIPVGDIY^1, 2, 3, 5^ | Gag/p24 | 254 | 262 | / | B*35:01, B*35:02, B*53:01 | (1, 2, 27, 28, 81, 115) |
| EIYKRWII^1, 2, 3, 4, 5^ | Gag/p24 | 260 | 267 | / | B*08:01, B*35:02, B*35:03, B*42:01, B*53:01 | (2, 6, 10, 12, 20, 39, 48, 61, 106, 116-118) |
| RRWIQLGLQK/ KRWIILGLNK^1, 2, 3, 4, 5^ | Gag/p24 | 263 | 272 | / | B*27:05 | \| (8, 18, 39, 48, 58, 67, 79, 84, 94, 104, 115, 119-130) \| \| --- \| |
| GLNKIVRMY^1, 2, 3, 4, 5^ | Gag/p24 | 269 | 277 | / | B*15:01, B*35:02, B*35:03, B*53:01 | (6, 12, 64, 109, 123, 131-134) |
| VRMYSPVSI^1, 2, 3, 4, 5^ | Gag/p24 | 274 | 282 | / | B*07:02, B*15:02, C*18:01 | (10, 12, 50, 96) |
| RMYSPTSI^1, 2, 3, 4, 5^ | Gag/p24 | 275 | 282 | / | B*52:01 | (99, 109, 135-140) |
| YSPVSILDI^1, 2, 3, 4, 5^ | Gag/p24 | 277 | 285 | / | C*01:02 | (2, 99, 109, 138, 141, 142) |
| FRDYVDRFF^1, 2, 3, 5^ | Gag/p24 | 293 | 301 | I285-L304 major homology region | A*01:01, B*42:02, C*18:01 | (10, 12, 17, 20, 96) |
| FRDYVDRFYK^1^ | Gag/p24 | 293 | 302 | I285-L304 major homology region | B*18:01 | (52-54) |
| RDYVDRFFKTL/ RDYVDRFYKTL^1, 2, 3, 4, 5^ | Gag/p24 | 294 | 304 | I285-L304 major homology region | A*24:02, A*68:02, B*44:02 | (12, 58, 103) |
| YVDRFYKTL^1, 2, 3, 4, 5^ | Gag/p24 | 296 | 304 | I285-L304 major homology region | A*02:07, C*03:04, A*01:01, A*26:01, B*07:02, B*15:03, B*15:10, C*03:03, C*03:04 | (6, 10, 19-23, 25, 30, 43, 50, 64) |
| DRFYKTLRA^1, 2, 3, 5^ | Gag/p24 | 298 | 306 | I285-L304 major homology region | B*14:02, B*14:01 | (10, 25, 64, 81, 103, 143) |
| AEQASQDVKNW^1, 2, 3, 5^ | Gag/p24 | 306 | 316 | / | B*15:03, B*15:24, B*35:01, B*35:08, B*44:02, B*58:01 | (2, 3, 12, 81, 118) |
| AEQASQEVKNWM^1, 3^ | Gag/p24 | 306 | 317 | / | B*35:01, B*35:08, B*58:01 | (12) |
| QASQEVKNW/  QATQDVKNW/  QATQDVKNW^1, 2, 3, 4, 5^ | Gag/p24 | 308 | 316 | / | B*35:01, B*35:08, B*53:01, B*57:01, B*58:01 | (1, 2, 10, 12, 18, 20, 26, 63, 79, 81-83, 85, 102, 105, 106, 144-147) |
| VKNWMTETL^3^ | Gag/p24 | 313 | 321 | / | B*48:01 | (148) |
| DCKTILKAL^3^ | Gag/p24 | 329 | 337 | / | B*08:01, B*15:03, B*35:02, B*35:03, B*53:01 | (12) |
| ACQGVGGPGHK^1, 2, 3, 4, 5^ | Gag/p24 | 349 | 359 | / | A*11:01, A*11:03, B*35:02, B*35:03, B*53:01 | (3, 12, 27, 109, 131, 132) |
| GPGHKARVL^1, 2, 3, 4, 5^ | Gag/p24 | 355 | 363 | / | B*07:02, B*42:01, | (2, 10, 14, 20, 50, 66, 81, 101, 109, 149, 150) |
| AEAMSQVTNS^1, 3^ | Gag/p2 | 364 | 373 | / | B*40:02, B*42:01, B*45:01 | (12, 22, 101) |
| APRKKGCWK^1^ | Gag/p7 | 407 | 415 | R409K drug resistance associated  G412D drug resistance associated | B*07:02 | (50) |
| TERQANFL^1, 2, 3^ | Gag/p7  /p1 | 427 | 434 | A431V drug resistance associated | B*40:02 | (1, 2, 109, 151) |
| RQANFLGKI^1, 3^ | Gag/p7  /p1 | 429 | 437 | N432 Gag-Pol-1 ribosomal slip site | B*13:02, B*15:24, B*48:01 | (6, 10, 20, 118, 148, 151-154) |
| FLGKIWPSYK^1, 4, 5^ | Gag/p1 | 433 | 442 | alternate ribosomal slip site produces transframe FLREDLAF protease inhibitor | A*02:01, A*02:05 | (1, 2, 12, 109, 151) |
| KELYPLTSL/ KELYPLASL^1, 3^ | Gag/p6 | 481 | 489 | L489 Vpr binding region | B*35:02, B*35:03, B*40:01, B*53:01, B*40:02 | (12) |
| NSPTRREL^1, 2, 5^ | Pol  /Gag_Pol | 24 | 31 | / | C*01:02 | (52-54, 93, 155) |
| ITLWQRPLV^1, 2, 5^ | Pol  /protease | 59 | 67 | / | A*68:02, A*74:01, B*35:02, B*35:03, B*53:01 | (10, 12, 156-161) |
| DTVLEEWNL/DTVLEEMNL^1, 3^ | Pol  /protease | 86 | 94 | V88I /L89F/I /E90Q /E91G/D/ M92V/I/L drug resistance associated | A*68:02, B*35:02, B*35:03, B*53:01 | (2, 3, 12, 60, 81, 101, 157, 162) |
| EEMNLPGRW^1, 3^ | Pol  /protease | 90 | 98 | E90Q /E91G/D/ M92V/I/L drug resistance associated | A*68:02, B*35:02, B*35:03, B*44:02, B*44:03 | (2, 3, 12, 60, 81, 101) |
| RQYDQILIEI^3^ | Pol  /protease | 113 | 122 | L119A/C/Q/S/P/T/ I120V drug resistance associated | B*13:02 | (51, 161) |
| GKKAIGTVL^1, 3^ | Pol  /protease | 124 | 132 | K126E/A127V/I/L/T /T130S/L132V drug resistance associated | B*15:03 | (3, 10) |
| KAIGTVLV^1, 3^ | Pol  /protease | 126 | 133 | K126E/A127V/I/L/T /T130S/L132V drug resistance associated | B*35:01, B*35:08 | (12) |
| LVGPTPVNI^1, 2, 3, 5^ | Pol  /protease | 132 | 140 | L132V /V138L/M/A/F/I/S/T /I140V/A drug resistance associated | A*02:01 | (2, 34, 64, 123, 161, 163, 164) |
| TPVNIIGRNML^1^ | Pol  /protease | 136 | 146 | V138L/M/A/F/I/S/T /I140V/A /I141V /N144G/D/S drug resistance associated | B*81:01 | (10) |
| FPISPIETV^2, 3^ | Pol/RT | 155 | 163 | / | B*54:01 | (165, 166) |
| IETVPVKL^1, 2, 3, 4, 5^ | Pol/RT | 160 | 167 | / | B*15:02, B*35:02, B*35:03, B*40:01, B*53:01 | (12, 49) |
| GPKVKQWPL^1, 2, 3, 5^ | Pol/RT | 173 | 181 | / | B*08:01, B*42:02 | (2, 12, 64, 106, 149, 167-169) |
| ALVEICTEM^1, 2, 3^ | Pol/RT | 188 | 196 | M196L drug resistance | A*02:01 | (170-172) |
| TVLDVGDAY^1, 2, 3, 4, 5^ | Pol/RT | 262 | 270 | V263I drug resistance  D265 catalytic site | B*15:02, B*35:01, B*35:02, B*35:03, B*53:01, B*57:01 | (2, 12, 36, 64, 114, 173) |
| SVPLDEGFRK^3^ | Pol/RT | 272 | 281 | V273I/P274S drug resistance | A*11:01 | (174) |
| VPLDEDFRKY^1, 2, 3, 4, 5^ | Pol/RT | 273 | 282 | V273I/P274S drug resistance | B*15:02, B*35:01, B*35:02, B*35:03, B*42:01, B*53:01 | (12, 36, 40, 81, 114, 173-175) |
| YTAFTIPSI^1, 2, 3^ | Pol/RT | 282 | 290 | I290L/M/T drug resistance | A*02:01, A*02:05, B*35:02, B*35:03, B*53:01 | (10, 12, 99, 173) |
| TAFTIPSI^1, 2, 3, 4, 5, 6^ | Pol/RT | 283 | 290 | I290L/M/T drug resistance | A*02:01, A*02:17, B*35:02, B*35:03, B*51:01, B*52:01, B*53:01 | (1, 52-54, 176-178) |
| IRYQYNVL^1^ | Pol/RT | 297 | 304 | / | B*14:01 | (10) |
| IRYQYNVLP^1, 3^ | Pol/RT | 297 | 305 | / | B*73:01 | (179) |
| LPQGWKGSPA^2, 3^ | Pol/RT | 304 | 313 | Gln highly conserved  Q306M drug resistance  S311A drug resistance  P312S drug resistance | B*54:01 | (165) |
| SPAIFQSSM^1, 2, 3, 4, 5^ | Pol/RT | 311 | 319 | Q316L drug resistance | A*68:02, B*07:02, B*15:17, B*35:01, B*42:01, B*42:02, B*81:01 | (3, 12, 66, 173) |
| SPAIFQSSMTK^3^ | Pol/RT | 311 | 321 | T320A drug resistance | A*11:01, A*68:01 B*07:02 | (174) |
| AIFQSSMTK^1, 3^ | Pol/RT | 313 | 321 | T320A drug resistance | A*03:01, A*11:01, A*68:01 | (3, 5, 173, 174, 180, 181) |
| KQNPDIVIY^1, 2, 3^ | Pol | 328 | 336 | V334D/E/F drug resistance  Y336C/I/V drug resistance | A*30:02, B*15:01, B*15:03, B*35:02, B*35:03, B*53:01, C*12:02 | (12, 20, 68, 135, 182, 183) |
| NPEIVIYQY/ HPDIVIYQY^1, 2, 3, 4, 5^ | Pol/RT | 330 | 338 | V334D/E/F drug resistance  Y336C/I/V drug resistance | B*35:01, B*35:02, B*35:03, B*35:08, B*42:01, B*53:01 | (12, 36, 40, 114, 173) |
| EIVIYQYMD ^3^ | Pol/RT | 332 | 340 | M339 I/V/T drug resistance | B*18:01 | (174) |
| VIYQYMDDL^1, 2, 3, 5^ | Pol/RT | 334 | 342 | D340/341 catalytic site | A*02:01, B*35:02, B*35:03, B*53:01 | (5, 12, 34, 64, 170, 173, 184, 185) |
| VIYQYMDDLYV^1, 3, 4^ | Pol/RT | 334 | 344 | Y343C/H/L drug resistance | A*02:01 | (186, 187) |
| IEELRQHLL^1, 3^ | Pol/RT | 357 | 365 | H363Y drug resistance  L365W drug resistance | B*40:01 | (49) |
| IVLPEKDSW^1, 3, 4^ | Pol/RT | 399 | 407 | / | B*35:01, B*35:02, B*35:03, B*35:08, B*53:01, B*57:01, B*57:02, B*57:03, B*58:01 | (2, 12, 58, 72) |
| LVGKLNWASQIY^1, 3^ | Pol/RT | 415 | 426 | / | B*15:01, B*15:03 | (12) |
| KLNWASQIY^1, 3^ | Pol/RT | 418 | 426 | / | A*30:02, B*15:01, B*15:02, B*35:02, B*35:03, B*53:01 | (10, 12) |
| QIYPGIKVR^1, 3, 5^ | Pol/RT | 424 | 432 | / | A*03:01, A*74:01 | (2, 3, 158, 188) |
| YPGIKVRQL^1, 2, 4, 5^ | Pol/RT | 426 | 434 | / | B*42:01, B*42:02, B*58:01 | (10, 12, 20, 21, 66, 81, 116, 149) |
| IPLTEEAEL^1, 2, 3, 6^ | Pol/RT | 448 | 456 | / | B*07:02, B*15:01, B*35:01, B*51:01, B*53:01 | (189-191) |
| ILKEPVHGV^1, 2, 3, 4, 5^ | Pol/RT | 464 | 472 | / | A*02:01, A*02:02, A*02:05 | (39, 44, 46, 67, 88, 171, 172, 192-197) |
| ILKEPVHGVYY^1, 2, 3^ | Pol/RT | 464 | 473 | Y473F drug resistance | B*15:01, B*15:10, C*03:03, C*12:02 | (3, 12, 47, 95, 183, 198) |
| IYQEPFKNLK^1, 2, 3^ | Pol/RT | 496 | 505 | / | A*11:01 | (10, 12, 173) |
| RMRGAHTNDV^1, 2, 3^ | Pol/RT | 511 | 520 | / | A*30:02, B*35:02, B*35:03, B*53:01 | (1) |
| IAMESIVIW^1, 2, 3, 4, 5, 6^ | Pol/RT | 530 | 538 | / | B*35:01, B*35:08, B*57:02, B*57:03, B*58:01 | (2, 3, 12, 20, 26, 86, 102, 106, 184) |
| PIQKETWETW^1, 2, 3^ | Pol/RT | 547 | 556 | / | A*32:01, B*15:17, B*35:01, B*35:08 | (1, 12, 199, 200) |
| GAETFYVDGA^1, 2, 3^ | Pol/RT  /RNase | 591 | 600 | / | A*68:02 | (12, 21, 184) |
| ETFYVDGA^1, 2, 3, 4, 5^ | Pol  /RNase | 593 | 600 | / | A*68:02 | (201) |
| ETFYVDGAANR^1, 3^ | Pol  /RNase | 593 | 603 | / | A*68:01, A*68:02, B*15:16 | (12) |
| ETKLGKAGY^1, 2, 5^ | Pol  /RNase | 604 | 612 | / | A*26:01, B*15:01 | (1, 12, 90) |
| VTDSQYALGI^1, 2, 3^ | Pol  /RNase | 651 | 660 | / | B*14:02, B*15:03, B*35:02, B*35:03, B*53:01 | (1, 12) |
| QIIEQLIKK^1, 2, 3, 4, 5^ | Pol  /RNase | 675 | 683 | / | A*11:01 | \| (2, 202) \| \| --- \| |
| LFLDGIDKA^1, 3^ | Pol  /Integrase | 715 | 723 | / | B*15:01 | (12) |
| LPPIVAKEI^1, 2, 3, 4, 5^ | Pol  /Integrase | 743 | 751 | / | B*07:05, B*42:01, B*51:01 | (10, 12, 20, 52-54, 81, 149, 178, 184) |
| THLEGKIIL^1, 3^ | Pol  /Integrase | 781 | 789 | / | B*15:10, B*15:17, B*39:01 | \| (10, 12, 81) \| \| --- \| |
| HVASGYIEA^1, 3^ | Pol  /Integrase | 793 | 801 | / | B*54:01 | (165) |
| IEAEVIPAET^1, 3^ | Pol  /Integrase | 799 | 808 | / | B*40:02, B*40:06 | \| (12, 137, 203) \| \| --- \| |
| HTDNGSNF^3^ | Pol  /Integrase | 829 | 836 | D831 catalytic | C*05:01 | (118) |
| STTVKAACWW^1, 3^ | Pol  /Integrase | 838 | 847 | / | B*57:01, B*58:01 | (12, 72, 81, 106, 110, 150) |
| IQQEFGIPY^1, 3, 4, 5^ | Pol  /Integrase | 850 | 858 | G855S drug resistance | B*15:01, B*15:03 | \| (10, 12, 13, 15, 67, 68) \| \| --- \| |
| VRDQAEHL^1, 3, 5^ | Pol  /Integrase | 880 | 887 | V880I drug resistance | C*18:01 | \| (10, 96) \| \| --- \| |
| KTAVQMAVF^1, 2, 3, 4, 5^ | Pol  /Integrase | 888 | 896 | / | B*15:24, B*35:02, B*35:03, B*53:01, B*57:01, B*57:03, B*58:01 | \| (12, 26, 59, 72) \| \| --- \| |
| FKRKGGIGGY^1, 2, 3, 4, 5^ | Pol  /Integrase | 900 | 909 | FKR Central polypurine tract  F900K drug resistance | B*15:03, B*27:05 | \| (10, 12, 15, 20, 67, 68, 81, 124) \| \| --- \| |
| KRKGGIGGY^1, 2, 3, 4, 5^ | Pol  /Integrase | 901 | 909 | / | B*15:03, B*27:02, B*27:05 | \| (2, 12, 67, 78, 122) \| \| --- \| |
| GERIVDII^1, 3^ | Pol  /Integrase | 912 | 919 | / | B*40:02 | (203, 204) |
| LQKQITKI^1, 3^ | Pol  /Integrase | 928 | 935 | / | B*52:01 | (198) |
| KIQNFRVYY^1, 2, 3, 4, 5^ | Pol  /Integrase | 934 | 942 | / | A*03:01, A*11:01, A*29:02, A*30:01, A*30:02, A*32:01, A*80:01 | \| (1, 20, 64, 116, 117) \| \| --- \| |
| YRDSRDPLW^1^ | Pol  /Integrase | 942 | 950 | / | B*38:01 | (2) |
| VVPRRKAKII^3^ | Pol  /Integrase | 974 | 983 | / | B*08:01 | (174) |
| VPRRKAKII^1^ | Pol  /Integrase | 975 | 983 | / | B*42:01 | (72) |
| RKAKIIRDY^1, 2, 3, 5^ | Pol  /Integrase | 978 | 986 | / | B*15:01, B*15:03 | \| (2, 3, 12, 20, 40, 68, 184, 205) \| \| --- \| |
| RKAKIIRDYGK^3^ | Pol  /Integrase | 978 | 988 | / | B*08:01 | (174) |
| KIIRDYGK^3^ | Pol  /Integrase | 981 | 988 | / | B*08:01 | (174) |
| IIKDYGKQM^1, 5^ | Pol  /Integrase | 982 | 990 | / | B*42:01 | (149) |
| RIRTWKSLVK^1, 3^ | Vif | 17 | 26 | / | A*03:01 | (174, 206) |
| IRTWKSLVK^3^ | Vif | 18 | 26 | / | A*30:01 | (174) |
| IRTWKSLVKH^3^ | Vif | 18 | 27 | / | B*27:05 | (174) |
| HMYISKKAK^1, 3^ | Vif | 28 | 36 | / | A*03:01, A*68:02, B*58:02 | (12, 206, 207) |
| ISKKAKGWF^1, 2, 3^ | Vif | 31 | 39 | / | B*35:01, B*35:02, B*35:03, B*35:08, B*53:01, B*57:01, B*58:01 | (12, 59, 206) |
| HPRVSSEVHI^1, 2, 3, 4, 5^ | Vif | 48 | 57 | / | B*07:02, B*42:01 | (3, 10, 12, 14, 15, 20, 21, 66, 206) |
| WHLGHGVSI/ WHLGQGVSI^1, 2, 3, 4, 5^ | Vif | 79 | 87 | / | B*15:10, B*15:10, B*35:02, B*35:03, B*38:01, B*53:01 | \| (3, 10, 12, 15, 20, 21, 66, 206) \| \| --- \| |
| LGHGVSIEW^1, 3, 4,^ | Vif | 81 | 89 | / | B*57:03 | (26, 206) |
| LADQLIHLHY^1^ | Vif | 102 | 111 | / | B*18:01 | (2, 55, 206) |
| KTKPPLPSVKK^1^ | Vif | 158 | 168 | / | A*03:01 | (206) |
| EAVRHFPRI^1, 3^ | Vpr | 29 | 37 | oligomerization region  vpr 17-33 alpha helix | A*68:02, B*35:01, B*35:02, B*35:03, B*35:08, B*51:01, B*53:01 | (12, 52) |
| AVRHFPRIW^1, 3^ | Vpr | 30 | 38 | oligomerization region  vpr 38-50 alpha helix | B*57:01, B*58:01 | (12, 52, 54) |
| VRHFPRIWL^1, 3, 4^ | Vpr | 31 | 39 | oligomerization region | A*68:02, B*27:02, B*27:05, B*35:02, B*35:03, B*53:01 | \| (8, 12, 67, 78, 122) \| \| --- \| |
| FPRIWLHGL^1, 2, 3, 5^ | Vpr | 34 | 42 | oligomerization region | B*07:02, B*15:10, B*42:01, B*81:01 | \| (10, 12, 20, 21, 72, 149) \| \| --- \| |
| ETYGDTWTGV^1, 2, 3, 4, 5^ | Vpr | 48 | 57 | vpr 55-77 alpha helix | A*68:02 | \| (10, 12, 21, 201) \| \| --- \| |
| DTWAGVEAIIR^1, 3^ | Vpr | 52 | 62 | vpr 55-77 alpha helix | A*68:01, A*68:02, B*35:02, B*35:03, B*53:01 | \| (12, 208) \| \| --- \| |
| AIIRILQQL^1^ | Vpr | 59 | 67 | vpr 55-77 alpha helix | A*02:01 | \| (34, 40, 150, 185) \| \| --- \| |
| CCFHCQVC^1^ | Tat | 30 | 37 |  | C*12:03 | \| (15, 30, 40, 52-54, 67, 209, 210) \| \| --- \| |
| FQTKGLGISY^1, 2, 3, 4, 5^ | Tat | 38 | 47 | Lys-41-Ala mutant deficient in transcription but functional in suppressing silencer-RNA | B*15:01, B*15:03, B*15:40 | \| (10, 12, 68) \| \| --- \| |
| ITKGLGISYGR^1, 2, 3^ | Tat | 39 | 49 | G48-N61 basic region, nuclear localization | A*68:01, B*15:01, B*15:03, B*35:02, B*35:03, B*53:01 | \| (12, 211-213) \| \| --- \| |
| EELLKTVRL^1, 2^ | Rev | 10 | 18 | / | B*44:02, B*44:03 | (214) |
| KAVRLIKFLY/ QAVRIIKILY^1, 2, 3, 4, 5^ | Rev | 14 | 23 | / | B*15:03, B*35:01, B*35:02, B*35:03, B*35:08, B*53:01, B*57:01, B*58:01 | \| (12, 20, 26, 59, 62, 114) \| \| --- \| |
| ERILSTYLGR^1, 3^ | Rev/exon 2 | 57 | 66 | / | A*03:01, B*58:01 | (12) |
| RPAEPVPLQL^1, 3, 5^ | Rev/exon 2 | 66 | 75 | / | B*07:02, B*42:02 | \| (3, 14, 118, 149) \| \| --- \| |
| SAEPVPLQL^3^ | Rev/exon 2 | 67 | 75 | / | C*05:01 | (118) |
| YRLGVGALI^1, 5^ | Vpu | 5 | 13 | Vpu transmembrane domain | C*18:01 | (10, 96) |
| EYRKILRQR^1, 3^ | Vpu | 29 | 37 | Vpu 29-82 cytoplasmic domain | B*35:02, B*35:03, B*53:01 | (12) |
| RVKEKYQHL^3^ | Env/gp120 | 2 | 10 | Env signal peptide | B*08:01 | (118) |
| AENLWVTVY^1, 2, 3^ | Env/gp120 | 31 | 39 | V36 gp120 interface contact with gp41 | B*15:17, B*15:40, B*18:01, B*44:03 | \| (12, 52-54, 131, 209) \| \| --- \| |
| AENLWVTVYY^1, 3^ | Env/gp120 | 31 | 40 | V36 gp120 interface contact with gp41 | A*68:02 | (209) |
| TVYYGVPVWK^1, 2, 3^ | Env/gp120 | 37 | 46 | Y40 gp120 interface contact with gp41 | A*03:01, B*15:40, B*42:02 | \| (3, 12, 75, 215, 216) \| \| --- \| |
| VPVWKEATTT^1, 3, 4^ | Env/gp120 | 42 | 51 | / | B*35:03, B*53:01, B*55:01 | (12) |
| VPVWKEATTTL^1, 3, 4,^ | Env/gp120 | 42 | 52 | / | B*35:01 | \| (10, 114) \| \| --- \| |
| KAYETEVHNVW^1, 3^ | Env/gp120 | 59 | 69 | V65 gp120 interface contact with gp41 | B*58:01 | \| (117, 217) \| \| --- \| |
| YETEVHNVW^1^ | Env/gp120 | 61 | 69 | V65 gp120 interface contact with gp41 | B*18:01 | \| (52-54) \| \| --- \| |
| DPNPQEVVL^1, 2, 3, 4, 5^ | Env/gp120 | 78 | 86 | / | B*15:02, B*35:01, B*35:02 | \| (2, 12, 114, 175, 218) \| \| --- \| |
| MHEDIISLW^1^ | Env/gp120 | 104 | 112 | / | B*38:01 | \| (2, 40) \| \| --- \| |
| SFEPIPIHY^1, 2^ | Env/gp120 | 209 | 217 | / | A*29:02, B*58:01 | \| (2, 12, 131, 132) \| \| --- \| |
| CAPAGFAIL^1, 3, 4^ | Env/gp120 | 218 | 226 | C218: linked to C 247 | C*01:02 | (219) |
| RPNNNTRKSI^1, 4, 5^ | Env/gp120 | 298 | 307 | R298 Coreceptor binding site inside V3  NNT301-303 glycosylation site | B*07:02, B*42:01, B*42:02 | \| (2, 14, 20, 65, 66) \| \| --- \| |
| HIGPGRAFY^1, 3^ | Env/gp120 | 310 | 318 | P313/R315/A316/F317 Coreceptor binding site | A*30:02, B*35:02, B*35:03 | (12) |
| RGPGRAFVTI^1, 3^ | Env/gp120 | 311 | 320 | P313/R315/A316/F317 Coreceptor binding site | B*42:02 | (12) |
| EIIGDIRQAY^1^ | Env/gp120 | 321 | 330 | / | A*25:01 | \| (52-54) \| \| --- \| |
| SFNCGGEFF^1, 2, 4, 5^ | Env/gp120 | 375 | 383 | N377 /E381 Coreceptor binding site  F382 gp120 interface contact with gp41 | B*15:16, C*04:01 | \| (123, 220, 221) \| \| --- \| |
| LPCRIKQII^1, 3^ | Env/gp120 | 416 | 424 | R419-424 Coreceptor binding site | B*51:01 | (12) |
| RIKQIINMW^1, 2^ | Env/gp120 | 419 | 427 | R419-424 Coreceptor binding site | A*32:01 | \| (21, 199) \| \| --- \| |
| RAIEAQQHL/ RAIEAQQHM^1, 2, 3, 4, 5^ | Env/gp41 | 557 | 565 | V496-613 Rev Responsive Element region  Q562 gp41 interface contact with gp120 | B*15:01, B*15:03, B*51:01, C*03:04, C*12:02 | \| (6, 12, 20, 52, 53, 64, 222) \| \| --- \| |
| QTRVLAIERYL^1, 5^ | Env/gp41 | 577 | 587 | LQA576-578 RRE stem | B*58:01, B*58:02 | \| (10, 223) \| \| --- \| |
| ERYLKDQQL^1, 2, 3, 4^ | Env/gp41 | 584 | 592 | Rev Responsive Element region | B*08:01, B*14:01, B*14:02 | \| (6, 10, 60, 62, 185) \| \| --- \| |
| RYLKDQQLL^1, 2, 3, 4^ | Env/gp41 | 585 | 593 | L593 gp41 interface contact with gp120 | A*23:01, A*24:02, C*07:02 | \| (20, 21, 40, 88, 205) \| \| --- \| |
| YLKDQQLL^1, 2, 4, 5^ | Env/gp41 | 586 | 593 | L593 gp41 interface contact with gp120 | A*24:02, B*08:01 | \| (4, 6, 224) \| \| --- \| |
| TAVPWNASW^1, 2, 3, 4^ | Env/gp41 | 606 | 614 | I595-617 fusion domain | B*35:01, B*35:08, B*58:01 | \| (12, 62, 113, 114, 225) \| \| --- \| |
| IVNRNRQGY^1^ | Env/gp41 | 704 | 712 | V705-856 gp41 cytoplasmic tail | A*30:02 | (2) |
| IVTRIVELL^1, 3^ | Env/gp41 | 777 | 785 | Y768-787 LLP-2 lentiviral lytic peptide alpha helix | A*02:05, B*15:17 | \| (2, 12) \| \| --- \| |
| GRRGWEALKY^1, 3, 4, 5^ | Env/gp41 | 786 | 795 | R787 subtype B and D 21bp deletion | B*15:40, B*27:05, B*35:02, B*35:03, B*53:01 | \| (12, 123) \| \| --- \| |
| RRGWEVLKY^1, 2, 4^ | Env/gp41 | 787 | 795 | G789-815 LLP-2 lentiviral lytic peptide alpha helix | A*01:01 | \| (2, 6) \| \| --- \| |
| KYCWNLLQY^1, 4^ | Env/gp41 | 794 | 802 | G789-815 LLP-2 lentiviral lytic peptide alpha helix | A*30:02 | \| (33, 226) \| \| --- \| |
| QELKNSAVSL^1, 3^ | Env/gp41 | 805 | 814 | G789-815 LLP-2 lentiviral lytic peptide alpha helix | B*08:01, B*35:02, B*35:03, B*40:01, B*53:01 | \| (2, 12, 49) \| \| --- \| |
| SLLNATDIAV^2^ | Env/gp41 | 813 | 822 | NAT816-818 Glycosite site | A*02:01 | (227) |
| LLNATDIAV^1, 2, 4^ | Env/gp41 | 814 | 822 | NAT816-818 Glycosite site | A*02:01, A*02:05 | \| (2, 6, 10, 20, 72, 185, 227) \| \| --- \| |
| IPRRIRQGL^1, 2, 3, 4, 5^ | Env/gp41 | 843 | 851 | R828-855 LLP-1 lentiviral lytic peptide alpha helix | B*07:02, B*42:01 | \| (2, 12, 14, 39, 66, 72, 79, 149) \| \| --- \| |
| RIRQGLERA^1, 2^ | Env/gp41 | 846 | 854 | R828-855 LLP-1 lentiviral lytic peptide alpha helix | A*02:05 | (1) |
| RQGLERALL^1^ | Env/gp41 | 848 | 856 | R828-855 LLP-1 lentiviral lytic peptide alpha helix | B*81:01 | (2) |
| WPTVRERM^1, 2, 4, 5^ | Nef | 13 | 20 | / | B*08:01, B*15:16 | \| (4, 6, 10, 12, 20, 72, 228, 229) \| \| --- \| |
| RMRRAEPAA^1, 3^ | Nef | 19 | 27 | / | B*15:01, B*58:01 | (12) |
| LEKHGAITS^1, 3^ | Nef | 37 | 45 | / | B*40:01 | (49) |
| FPVTPQVPL^1, 2, 3, 5^ | Nef | 68 | 76 |  | A*68:02, B*07:02 | \| (3, 12, 14, 65, 156, 188) \| \| --- \| |
| FPVTPQVPLR^1, 3^ | Nef | 68 | 77 | Nef 77-82 phosphorylation site | A*68:02, B*07:02 | \| (3, 12) \| \| --- \| |
| TPQVPLRPM/ RPQVPLRPM^1, 2, 3, 4, 5^ | Nef | 71 | 79 | Nef 77-82 phosphorylation site | A*68:02, B*07:02, B*07:02, B*35:01, B*35:02, B*35:03, B*42:01, B*42:02, B*53:01, B*67:01, B*81:01 | \| (10, 12, 14, 20-22, 66, 72, 81, 116, 117, 149, 159, 184, 230-232) \| \| --- \| |
| RPQVPLRPMTY^1, 2, 3, 4, 5, 6^ | Nef | 71 | 81 | Nef 77-82 phosphorylation site  R77A mutation impairs downregulation of class I MHC  P78 needed for downregulation of class I MHC | B*35:01, B*67:01, C*07:02 | \| (9, 113, 159, 231, 233) \| \| --- \| |
| QVPLRPMTYK^1, 2, 3, 4, 5^ | Nef | 73 | 82 | Nef 77-82 phosphorylation site | A*02:01, A*03:01, A*11:01, B*35:02, B*35:03, B*53:01 | \| (2, 4, 6, 9, 12, 18, 22, 60, 79, 103, 202, 234) \| \| --- \| |
| VPLRPMTY^1, 2, 3, 4, 5^ | Nef | 74 | 81 | Nef 77-82 phosphorylation site | B*35:01, B*35:02, B*35:03, B*53:01 | \| (1, 2, 12, 36, 113, 114, 187, 231, 233) \| \| --- \| |
| PLRPMTYK^1, 3^ | Nef | 75 | 82 | Nef 77-82 phosphorylation site | A*11:01, B*15:01 | (12) |
| LRPMTYKAA^1, 3^ | Nef | 76 | 84 | Nef 77-82 phosphorylation site | B*27:03 | (17) |
| RPMTYKAAL^1, 2, 3, 4, 5^ | Nef | 77 | 85 | Nef 77-82 phosphorylation site | B*07:02, B*67:01, B*42:01, B*42:02 | \| (3, 12, 14, 149, 159, 188) \| \| --- \| |
| KAAFDLSFF^1, 2, 3, 4, 5^ | Nef | 82 | 90 | Nef 77-82 phosphorylation site | B*57:01, B*57:02, B*57:03, B*58:01, C*12:02 | (21, 22, 26, 62, 72, 81, 101, 110, 222, 235-237) |
| KAAVDLSHFL^1^ | Nef | 82 | 91 | Nef 77-82 phosphorylation site  F90-91 3’polypurine tract  F90A mutation causes modest defect in viral growth rate | C*08:04 | (22) |
| GAFDLSFFL/ AAVDLSHFL/ AAFDLSFFL/ AALDLSHFL^1, 2, 3, 4, 5^ | Nef | 83 | 91 | F90-91 3’polypurine tract  F90A mutation causes modest defect in viral growth rate | A*02:05, A*02:06, B*15:17, B*14:02, B*42:02, B*35:02, B*35:03, B*53:01, B*57:03, C*06:02 | \| (9, 10, 12, 21, 22, 99, 116, 117, 179, 226) \| \| --- \| |
| AVDLSHFLK^1, 2, 3, 4, 5^ | Nef | 84 | 92 | F90-91 3’polypurine tract  F90A mutation causes modest defect in viral growth rate | A*03:01, A*11:01, B*15:03, B*15:17 | \| (2, 3, 6, 12, 79, 202, 234) \| \| --- \| |
| FLKEKGGL^1, 2, 3, 4, 5^ | Nef | 90 | 97 | F90-91 3’polypurine tract  F90A mutation causes modest defect in viral growth rate | B*08:01, B*35:01, B*35:08 | \| (2, 6, 9, 10, 20, 79, 106, 117, 150, 238-241) \| \| --- \| |
| KEKGGLEGL^1, 2, 3^ | Nef | 92 | 100 | / | B*35:02, B*35:03, B*40:01, B*40:02, B*44:03, B*53:01 | \| (1, 2, 9, 12, 49, 60, 81) \| \| --- \| |
| KEKGGLEGLIY^1, 3, 4^ | Nef | 92 | 102 | / | B*44:03 | (242) |
| RRQDILDLWI^1^ | Nef | 105 | 114 | R105 essential for dimerization | B*27:05 | (119) |
| RRQDILDLWVY/KRQEILDLWVY^1, 2, 3^ | Nef | 105 | 115 | W113A mutation causes modest defect in viral growth rate | B*18:01, B*58:01, C*07:01, C*07:02, B*35:02, B*35:03 | \| (3, 10, 12, 20-22, 43, 62, 81, 150, 243) \| \| --- \| |
| RQDILDLWI^1, 2, 3^ | Nef | 106 | 114 | I114A mutation causes modest defect in viral growth rate | B*13:02, B*15:24, B*13:02 | \| (12, 21, 51) \| \| --- \| |
| QEILDLWVY^1, 3, 4, 5^ | Nef | 107 | 115 | I114A mutation causes modest defect in viral growth rate | B*18:01, B*44:02, B*44:03 | \| (242, 244) \| \| --- \| |
| HTQGYFPDW^1, 2, 3, 4, 5^ | Nef | 116 | 124 | D123 essential for dimerization | B*15:03, B*57:01, B*57:03, B*58:01 | (2, 12, 21, 26, 59, 60, 62, 79, 102, 103, 235, 245) |
| TQGYFPDWQNY^1, 2, 3, 4^ | Nef | 117 | 127 | D123 essential for dimerization | B*15:01 | \| (6, 12, 137) \| \| --- \| |
| GYFPDWQNY^1, 3^ | Nef | 119 | 127 | D123 essential for dimerization | B*58:01 | (179) |
| YFPDWQNYT^1, 2, 3, 5^ | Nef | 120 | 128 | D123 essential for dimerization | B*35:01, B*37:01, B*57:01, B*58:01 | \| (64, 235) \| \| --- \| |
| FPDWQNYTP^2, 3^ | Nef | 121 | 129 | D123 essential for dimerization | B*54:01 | (165) |
| NYTPGPGIRY^1, 5^ | Nef | 126 | 135 | GPG130-132 Bata turn: G-P-G | A*24:02 | (246) |
| YTPGPGIRY^1, 3^ | Nef | 127 | 135 | GPG130-132 Bata turn: G-P-G | B*35:02, B*35:03, B*53:01 | \| (12, 72) \| \| --- \| |
| TPGPGVRYPL^1, 2, 3, 4, 5^ | Nef | 128 | 137 | GPG130-132 Bata turn: G-P-G | B*07:02, B*35:01, B*35:02, B*35:03, B*42:01, B*42:02, B*53:01, B*81:01 | \| (3, 9, 10, 12, 14, 15, 21, 22, 36, 66, 67, 116, 149, 150, 247) \| \| --- \| |
| TRYPLTFGW^1, 3, 4^ | Nef | 133 | 141 | / | A*68:02, B*35:01, B*35:02, B*35:03, B*35:08, B*42:01, B*53:01 | \| (9, 12) \| \| --- \| |
| RYPLTFGW^1, 2, 3, 4^ | Nef | 134 | 141 | / | A*23:01, A*24:02, B*35:02, B*35:03, B*53:01 | \| (2, 3, 10, 12, 21, 61, 187, 248-251) \| \| --- \| |
| RYPLTFGWCY^1, 2, 3, 4, 5^ | Nef | 134 | 143 | / | A*24:02, C*07:02, A*23:01 | \| (2, 22, 79, 92, 93, 99, 175, 246, 249, 250, 252, 253) \| \| --- \| |
| YPLTFGWCY/ YPLTFGWCF^1, 2, 3, 4, 5^ | Nef | 135 | 143 | / | B*07:02, B*18:01, B*35:01, B*35:02, B*35:03, B*35:08, B*53:01, B*58:01, B*67:01 | \| (1, 2, 9, 10, 20, 22, 43, 52-54, 81, 93, 113, 114, 159, 205, 239, 254, 255) \| \| --- \| |
| PLTFGWCYKL^1, 3^ | Nef | 136 | 145 | / | A*02:01, B*35:02, B*35:03, B*53:01, B*58:01 | \| (5, 12) \| \| --- \| |
| LTFGWCFKL^1, 2, 3^ | Nef | 137 | 145 | / | A*02:01, A*68:02, B*15:17, B*15:40 | \| (6, 12, 21, 156, 256-261) \| \| --- \| |
| VLEWRFDSRL^1, 2, 3, 5^ | Nef | 180 | 189 | / | A*02:01 | \| (5, 12, 260, 262-265) \| \| --- \| |
| WRFDSRLAF^1, 3^ | Nef | 183 | 191 | / | B*15:03, B*27:05 | \| (1, 2, 12, 40, 68, 72, 150, 228, 260) \| \| --- \| |

**Table S2. List of CD4^+^ T cell epitopes validated from HIV proteins.**

| **Epitope** | **Protein** | **HXB2 start** | **HXB2 end** | **Sites of interest** | **HLA** | **Reference** |
| --- | --- | --- | --- | --- | --- | --- |
| SGGELDRWEKIRLRPGGK^1, 3^ | Gag/p17 | 9 | 26 | E12K drug resistance | DRB1*07:01, DRB1*11:01, DRB1*13:01, DRB1*13:02, DRB3*01:01 | (266-270) |
| LRPGGKKKYKLKHIV^1, 5^ | Gag/p17 | 21 | 35 | E17-L31 binds plasma membrane | DRB1*13:02 | (266, 269-274) |
| GKKKYKLKHIVWASREL^1, 3, 5^ | Gag/p17 | 25 | 41 | V35I/E40K drug resistance | DRB1*01:01, DRB1*07:01, DRB1*08:01, DRB1*11:01, DRB1*13:02, DRB1*13:03, DRB3*01:01, DRB3*03:01, DRB4*01:01, DRB5*01:01 | (266) |
| KHIVWASRELERFAV^1, 3, 5^ | Gag/p17 | 32 | 46 | V35I/E40K drug resistance | DRB1*03:01, DRB1*13:01, DRB1*13:02, DRB1*13:03, DRB3*01:01, DRB5*01:01 | (266-269, 274) |
| ASRELERFAVNPGLL^1, 3, 5^ | Gag/p17 | 37 | 51 | E40K drug resistance | DRB1*01:01, DRB1*04:01, DRB1*04:05, DRB1*07:01, DRB1*13:01, DRB1*13:02, DRB1*15:01, DRB4*01:01 | (266, 267, 270, 272, 275, 276) |
| LERFAVNPGLL^1, 3, 5^ | Gag/p17 | 41 | 51 | / | DRB1*13:02 | (267) |
| LERFAVNPGLLETSE^1^ | Gag/p17 | 41 | 55 | / | DRB1*01:01 | (277) |
| ERFAVNPGLLETSEGCR^1, 5^ | Gag/p17 | 42 | 58 | C57 essential for particle formation | DRB1*01:01, DRB1*04:05, DRB1*11:01, DRB1*13:02, DRB1*13:03, DRB1*15:01, DRB3*03:01 | (266, 267) |
| TGSEELRSLYNTVATLY^1, 3, 5^ | Gag/p17 | 70 | 86 | L75R drug resistance | DRB1*04:05, DRB1*07:01 | (266-269) |
| EELRSLYNTVATLYC^1, 3, 5^ | Gag/p17 | 73 | 87 | L75R drug resistance | DRB1*01:01 | (278) |
| EELRSLYNTVATLYCVH^1, 5^ | Gag/p17 | 73 | 89 | L75R drug resistance | DRB1*01:01, DRB1*04:01, DRB1*04:05, DRB1*07:01, DRB1*11:01, DRB1*13:02, DRB1*15:01 | (279) |
| SLYNTVATLYCVHQR^1^ | Gag/p17 | 77 | 91 | / | DRB1*01:01 | (277) |
| SLYNTVATLYCVHQRIEV^1, 3, 5^ | Gag/p17 | 77 | 94 | / | DRB1*01:01, DRB1*04:01, DRB1*04:05, DRB1*07:01, DRB1*13:02, DRB1*14:01, DRB5*01:01 | (266, 267, 269, 280) |
| PIVQNIQGQ^1, 3^ | Gag/p24 | 133 | 150 | / | DRB1*01:01 | (281) |
| IVQNLQGQMVHQAISPR^1, 3^ | Gag/p24 | 134 | 150 | / | DRB1*09:01 | (266) |
| PRTLNAWVKVVEEKAF^1, 3, 5^ | Gag/p24 | 149 | 164 | / | DRB1*13:01, DRB1*13:04 | (266, 269) |
| WVKVVEEKAFSPEVIPMF^1, 3^ | Gag/p24 | 155 | 172 | / | DRB1*11:01 | (266, 267, 276, 280) |
| AFSPEVIPMFSALSEGA^1, 3, 5^ | Gag/p24 | 163 | 179 | / | DRB1*04:01, DRB1*07:01, DRB1*15:01, DRB4*01:01 | (266, 267, 269, 270, 282) |
| SPEVIPMFSALSE^1, 5^ | Gag/p24 | 165 | 177 | / | DRB1*01:01, DRB1*03:01, DRB1*04:01, DRB1*04:05, DRB1*11:01, DRB1*15:01 | (279) |
| ETINEEAAEWDRVHPVHA^1, 3^ | Gag/p24 | 203 | 220 | Pro217-225 Cyclophilin A binding site  H219Q drug resistance | DRB1*01:01 | (281) |
| AAEWDRLHPVHAGPIA^1, 3, 5^ | Gag/p24 | 209 | 224 | H219Q drug resistance | DRB1*07:01 | (266, 269) |
| LHPVHAGPIAPGQMREPR^1, 5^ | Gag/p24 | 215 | 232 | H219Q drug resistance | DRB1*11:01 | (266, 282) |
| GSDIAGTTSTLQEQI^1^ | Gag/p24 | 233 | 247 | / | DRB1*01:01 | (277) |
| SDIAGTTSTLQEQIGWM^1, 5^ | Gag/p24 | 234 | 250 | / | DRB1*04:04, DRB1*15:02 | (266) |
| STLQEQIGWMTNNPP^3^ | Gag/p24 | 241 | 255 | / | DRB1*01:01 | (283) |
| STLQEQIGWMTNNPPIPV^1, 3, 5^ | Gag/p24 | 241 | 258 | / | DRB1*01:01, DRB1*13:03, DRB3*01:01, DRB3*03:01 | (266, 269) |
| WMTNNPPIPVGEIYK^3^ | Gag/p24 | 249 | 263 | / | DRB1*01:01 | (284) |
| WMTNNPPIPVGEIYKRWI^1, 5^ | Gag/p24 | 249 | 266 | / | DRB3*01:01, DRB3*03:03 | (266) |
| TNNPPIPBGEIYKRW^5^ | Gag/p24 | 251 | 265 | / | DRB1*13:01 | (285) |
| NPPIPVGEIYKRWII^1, 5^ | Gag/p24 | 253 | 267 | / | DRB1*01:01 | (271) |
| PVGEIYKRWIILGLN^1^ | Gag/p24 | 257 | 271 | / | DRB1*01:01 | (275) |
| IYKRWIILGLNKIVR^1^ | Gag/p24 | 261 | 275 | / | DRB1*01:01 | (277) |
| WIILGLNKIVRM^1, 3^ | Gag/p24 | 265 | 276 | / | DRB1*01:01, DRB1*04:01, DRB1*04:05, DRB1*11:01, DRB1*13:02, DRB1*15:01, DRB5*01:01 | (84, 275, 277, 278, 283, 286) |
| WIILGLNKIVRMYSP^1^ | Gag/p24 | 265 | 279 | / | [DQB1*06:02, DQB1*06:04, DRB1*13:02, DRB1*15:01, DRB3*03:01, DRB5*01:01](https://www.hiv.lanl.gov/mojo/immunology/patient/details.html?id=3751),  [DQB1*06:01, DRB1*13:03, DRB1*15:02, DRB5*01:02](https://www.hiv.lanl.gov/mojo/immunology/patient/details.html?id=3752) | (277) |
| WIILGLNKIVRMYSPVSI^1, 3^ | Gag/p24 | 265 | 282 | / | DRB1*01:01, DRB1*03:02, DRB1*04:01, DRB1*04:05, DRB1*07:01, DRB1*11:01, DRB1*13:01, DRB1*13:02, DRB1*15:01, DRB1*15:02, DRB5*01:01 | (266-268, 274) |
| GLNKIVRMYSPTSIL^1^ | Gag/p24 | 269 | 283 | / | DRB1*01:01 | (275) |
| GPKEPFRDYVDRFYKTLR^1, 5^ | Gag/p24 | 288 | 305 | I285-L304 major Homology region | DRB1*13:01 | (266) |
| DYVDRFYKTLRAE^1, 3, 4^ | Gag/p24 | 295 | 307 | / | DRB1*0101 | (268) |
| YVDRFYKTLRAEQASQEV^1, 3, 5^ | Gag/p24 | 296 | 313 | / | DRB1*01:01, DRB1*04:01, DRB1*04:05, DRB1*07:01, DRB1*08:01, DRB1*10:01, DRB1*11:01, DRB1*13:01, DRB1*13:02, DRB1*13:03, DRB1*15:01, DRB1*15:02, DRB4*01:01, DRB5*01:01 | (266-268) |
| VDRFYKTLRAEQASQ^1^ | Gag/p24 | 297 | 311 | / | DRB1*01:01 | (275) |
| DRFYKTLRAEQASQ^4^ | Gag/p24 | 298 | 311 | / | DRB1*04:01 | (287) |
| RFYKTLRAEQAS^1, 3, 5^ | Gag/p24 | 299 | 310 | / | DRB1*01:01, DRB1*04:01, DRB1*04:05, DRB1*07:01, DRB1*11:01, DRB1*15:01, DRB5*01:01 | (267) |
| RFYKTLRAEQASQEVK^3^ | Gag/p24 | 299 | 314 | / | DRB1*01:01 | (288) |
| FYKTLRAEQASQ^1, 3, 5^ | Gag/p24 | 300 | 311 | / | DRB1*01:01, DRB1*04:01, DRB1*11:01, DRB5*01:01 | (267) |
| FYKTLRAEQASQE^1, 3, 5^ | Gag/p24 | 300 | 312 | / | DRB1*01:01, DRB1*04:01, DRB1*04:05, DRB1*11:01, DRB1*15:01, DRB5*01:01 | (267) |
| YKTLRAEQASQEVKN^1^ | Gag/p24 | 301 | 315 | / | DRB1*01:01 | (275) |
| RAEQASQEVKNWMTE^1^ | Gag/p24 | 305 | 319 | / | DRB1*01:01 | (277) |
| EVKNWMTETLLVQNA^1, 5^ | Gag/p24 | 312 | 326 | / | DRB1*14:01 | (266) |
| TNSATIMMQRGNFRNQRK^1, 5^ | Gag/p2/p7 | 371 | 388 | G381S drug resistance | DRB1*15:02 | (266) |
| CFNCGKEGHIAKNCRAPR^1, 5^ | Gag/p7 | 392 | 409 | C392-C405 Zinc knuckle1 | DRB1*09:01, DRB1*13:01 | (266) |
| HIAKNCRAPRKKGCWK^1, 5^ | Gag/p7 | 400 | 415 | R409K drug resistance  G412D drug resistance | DRB1*07:01, DRB1*13:01 | (266) |
| RAPRKKGCWKCGKEGHQM^1, 5^ | Gag/p7 | 406 | 423 | C413-C426 Zinc knuckle2 | DRB1*09:01 | (266) |
| RQANFLGKIWPSHKGR^1, 3, 5^ | Gag/p7/p1 | 429 | 444 | A431V drug resistance  N432 Gag-pol-1ribosomal slip site | DRB1*01:01, DRB1*04:01, DRB1*04:05, DRB1*11:01, DRB1*13:01, DRB1*13:02, DRB1*15:01, DRB5*01:01 | (267) |
| GKIWPSHKGRPGNFLQSR^1, 5^ | Gag/p1 | 435 | 452 | / | DRB1*07:01, DRB1*13:01, DRB1*15:01 | (266) |
| FLQSRPEPTAPPEESFRF^1, 5^ | Gag/p6 | 448 | 465 | L449F drug resistance  P455-E460 Vpr binding region | DRB1*07:01 | (266) |
| DKELYPLASLRSLFG^1, 5^ | Gag/p6 | 480 | 494 | / | DRB1*01:01, DRB1*04:01, DRB1*04:05, DRB1*07:01, DRB1*11:01, DRB1*15:01, DRB5*01:01 | (279) |
| QRPLVTIKIGGQLKE^1, 5^ | Pol/protease | 63 | 77 | G72/A/E  /K76T/I/M/R drug resistance | DRB1*01:01, DRB1*11:01, DRB1*15:01, DRB5*01:01 | (279) |
| TPVNIIGRNLLTQIG^1, 5^ | Pol/protease | 136 | 150 | V138L/M/A/F/I/S/T  /I140V/A /I141V /N144G/D/S /Q148K  drug resistance | DRB1*01:01, DRB1*11:01, DRB1*13:02, DRB1*15:01 | (279) |
| VIWGKTPKFKLPIQKETW^1, 5^ | Pol/RT | 536 | 553 | T541I drug resistance | DRB1*13:01, DRB3*01:01 | (266) |
| GKIILVAVHVASGYI^1, 5^ | Pol/Integrase | 785 | 799 | / | DRB1*01:01, DRB1*04:01, DRB1*04:05, DRB1*07:01, DRB1*11:01, DRB1*13:02, DRB1*15:01, DRB5*01:01 | (279) |
| SLQYLALVALVAPKK^1, 5^ | Vif | 144 | 158 | S144 phosphorylated | DRB1*01:01, DRB1*04:01, DRB1*04:05, DRB1*07:01, DRB1*11:01, DRB1*15:01, DRB5*01:01 | (279) |
| EAIIRILQQLLFIHF^1, 5^ | Vpr | 58 | 72 | / | DRB1*01:01, DRB1*04:05, DRB1*15:01 | (279) |
| QQLLFIHFRIGCRHSRIG^1, 5^ | Vpr | 65 | 82 | / | DRB1*01:01, DRB1*04:05, DRB1*07:01, DRB1*11:01, DRB1*13:02, DRB1*15:01, DRB5*01:01 | (279) |
| TKGLGISYGRKKRRQRRR^1, 5^ | Tat | 40 | 57 | G48-N61 nuclear localization | DRB1*13:02, DRB1*13:03, DRB3*01:01, DRB3*03:01 | (266) |
| ELLKTVRLIKFLYQSNP^1, 5^ | Rev | 11 | 27 | N26-Q101 Rev exon 2 | DRB1*01:01, DRB1*03:01, DRB1*04:01, DRB1*04:05, DRB1*07:01, DRB1*11:01, DRB1*13:02, DRB1*15:01 | (279) |
| PSPEGTRQARRNRRRRW^1, 5^ | Rev | 29 | 45 | N26-Q101 Rev exon 2 | DRB1*13:01, DRB3*01:01 | (266) |
| TMLLGMLMICSAA^1, 5^ | Env | 19 | 31 | Env signal peptide | DRB1*01:01, DRB1*04:05, DRB1*11:01 | (279) |
| AAEQLWTVYYGVPPVW^1, 5^ | Env/gp120 | 30 | 45 | A31 signal peptide cleavage site  V36 /Y40 gp120 interface contact with gp41 | DRB1*11:01, DRB1*15:01 | (266) |
| NVTENFNMWKNNMVEQMH^1, 5^ | Env/gp120 | 88 | 105 | NVT88-90 glycosite | DRB1*11:01, DRB1*15:01, DRB1*15:02 | (266) |
| NFNMWKNNMVEQM^1, 3, 5^ | Env/gp120 | 92 | 104 | N92 gp120 interface contact with gp41 | DRB1*15:02 | (289) |
| DKMQKEYALLYKLDI^1, 5^ | Env/gp120 | 167 | 181 | L179-I181 tripeptide binds integrin | DRB1*03:01, DRB1*12:01, DRB1*15:01, DRB4*01:01, DRB5*01:01 | (290) |
| DKKQKVHALFYKLDIV^1, 3, 5^ | Env/gp120 | 167 | 182 | L179-I181 tripeptide binds integrin | DRB1*12:02 | (289) |
| NTSYRLISCNTSVI^1, 5^ | Env/gp120 | 188 | 201 | D185-S190 V2 hypervariable region  S195 Coreceptor-specific (R5/X4) site  C196 CD4 contact residue, side-chain-only contact | DRB1*01:01, DRB1*04:01, DRB1*04:05, DRB1*07:01, DRB1*11:01, DRB1*13:02, DRB1*15:01, DRB5*01:01 | (279) |
| KVSFEPIPIHYCAPAGFA^1, 5^ | Env/gp120 | 207 | 224 | K207 Coreceptor binding site  C218 linked to C247 | DRB1*01:01, DRB1*11:01, DRB3*01:01 | (266) |
| SELYLYKVVKIEPLGVAP^1, 5^ | Env/gp120 | 481 | 498 | G495 gp120 interface contact with gp41  V496-S613 RRE region | DRB1*01:01, DRB1*04:01, DRB1*04:05, DRB1*07:01, DRB1*11:01, DRB1*13:02, DRB1*15:01, DRB5*01:01 | (279) |
| RDLLLIVTRIVELLGR^1, 5^ | Env/gp41 | 772 | 787 | V705-L856 gp41 cytoplasmic tail  Y768-R787 LLP-2 lentiviral lytic peptide alpha helix | DRB1*01:01, DRB1*07:01, DRB1*11:01 | (279) |
| RSVVGWPAVRERMRRA^1, 5^ | Nef | 8 | 23 | / | DRB1*13:02 | (266, 291) |
| YKAAVDLSHFLKEKGGL^1, 5^ | Nef | 81 | 97 | F90-L91 3’polypurine tract  F90A mutation causes modest defect in viral growth rate | DRB1*07:01, DRB1*08:04 | (266) |
| LWVYHTQGYFPDWQNY^1, 5^ | Nef | 112 | 127 | W113A/I114Amutation causes modest defect in viral growth rate | DRB1*07:01, DRB1*13:01, DRB1*14:01, DRB3*01:01, DRB4*01:01 | (266) |
| PEKEVLVWKFDSRLAFHH^1, 3, 5^ | Nef | 176 | 193 | mutation causes modest defect in viral growth rate | DRB1*01:01, DRB1*04:01, DRB1*07:01, DRB1*11:01, DRB1*13:02, DRB1*15:01, DRB5*01:01 | (267) |
| VLEWRFDSRLAFHHV^1, 5^ | Nef | 180 | 194 | / | DRB1*01:01, DRB1*07:01, DRB1*11:01, DRB1*15:01, DRB5*01:01 | (279) |
| KFDSRLAFHHMARELH^1^ | Nef | 184 | 199 | / | DRB1*01:01, DRB1*03:02, DRB1*07:01, DRB1*11:01, DRB1*13:02, DRB1*13:03, DRB1*15:01, DRB3*03:01, DRB5*01:01 | (267) |

**Method to Validate the Candidate Epitopes:** 1, 2, 3, 4, 5 and 6 stand for Elispot, Chromium-release assay, ICS, Tetramer binding, HLA binding and TCR binding, respectively.

**Reference**

1. Sabbaj S, Bansal A, Ritter GD, Perkins C, Edwards BH, Gough E, et al. Cross-reactive CD8+ T cell epitopes identified in US adolescent minorities. Journal of acquired immune deficiency syndromes (1999). 2003;33(4):426-38.

2. Kunwar P, Hawkins N, Dinges WL, Liu Y, Gabriel EE, Swan DA, et al. Superior control of HIV-1 replication by CD8+ T cells targeting conserved epitopes: implications for HIV vaccine design. PloS one. 2013;8(5):e64405.

3. Du VY, Bansal A, Carlson J, Salazar-Gonzalez JF, Salazar MG, Ladell K, et al. HIV-1-Specific CD8 T Cells Exhibit Limited Cross-Reactivity during Acute Infection. J Immunol. 2016;196(8):3276-86.

4. Milicic A, Edwards CT, Hué S, Fox J, Brown H, Pillay T, et al. Sexual transmission of single human immunodeficiency virus type 1 virions encoding highly polymorphic multisite cytotoxic T-lymphocyte escape variants. Journal of virology. 2005;79(22):13953-62.

5. Kleen TO, Asaad R, Landry SJ, Boehm BO, Tary-Lehmann M. Tc1 effector diversity shows dissociated expression of granzyme B and interferon-gamma in HIV infection. AIDS (London, England). 2004;18(3):383-92.

6. Colleton BA, Huang XL, Melhem NM, Fan Z, Borowski L, Rappocciolo G, et al. Primary human immunodeficiency virus type 1-specific CD8+ T-cell responses induced by myeloid dendritic cells. Journal of virology. 2009;83(12):6288-99.

7. Ammaranond P, van Bockel DJ, Petoumenos K, McMurchie M, Finlayson R, Middleton MG, et al. HIV immune escape at an immunodominant epitope in HLA-B*27-positive individuals predicts viral load outcome. Journal of immunology (Baltimore, Md : 1950). 2011;186(1):479-88.

8. Adland E, Hill M, Lavandier N, Csala A, Edwards A, Chen F, et al. Differential Immunodominance Hierarchy of CD8(+) T-Cell Responses in HLA-B*27:05- and -B*27:02-Mediated Control of HIV-1 Infection. Journal of virology. 2018;92(4).

9. Almeida CA, Bronke C, Roberts SG, McKinnon E, Keane NM, Chopra A, et al. Translation of HLA-HIV associations to the cellular level: HIV adapts to inflate CD8 T cell responses against Nef and HLA-adapted variant epitopes. Journal of immunology (Baltimore, Md : 1950). 2011;187(5):2502-13.

10. Kiepiela P, Ngumbela K, Thobakgale C, Ramduth D, Honeyborne I, Moodley E, et al. CD8+ T-cell responses to different HIV proteins have discordant associations with viral load. Nature medicine. 2007;13(1):46-53.

11. Lissina A, Fastenackels S, Inglesias MC, Ladell K, McLaren JE, Briceño O, et al. The link between CD8⁺ T-cell antigen-sensitivity and HIV-suppressive capacity depends on HLA restriction, target epitope and viral isolate. Aids. 2014;28(4):477-86.

12. Pereyra F, Heckerman D, Carlson JM, Kadie C, Soghoian DZ, Karel D, et al. HIV control is mediated in part by CD8+ T-cell targeting of specific epitopes. Journal of virology. 2014;88(22):12937-48.

13. Kinloch NN, Lee GQ, Carlson JM, Jin SW, Brumme CJ, Byakwaga H, et al. Genotypic and Mechanistic Characterization of Subtype-Specific HIV Adaptation to Host Cellular Immunity. Journal of virology. 2019;93(1).

14. Kløverpris HN, Adland E, Koyanagi M, Stryhn A, Harndahl M, Matthews PC, et al. HIV subtype influences HLA-B*07:02-associated HIV disease outcome. AIDS research and human retroviruses. 2014;30(5):468-75.

15. Kløverpris HN, McGregor R, McLaren JE, Ladell K, Stryhn A, Koofhethile C, et al. Programmed death-1 expression on HIV-1-specific CD8+ T cells is shaped by epitope specificity, T-cell receptor clonotype usage and antigen load. Aids. 2014;28(14):2007-21.

16. Geldmacher C, Currier JR, Herrmann E, Haule A, Kuta E, McCutchan F, et al. CD8 T-cell recognition of multiple epitopes within specific Gag regions is associated with maintenance of a low steady-state viremia in human immunodeficiency virus type 1-seropositive patients. Journal of virology. 2007;81(5):2440-8.

17. Huang XL, Fan Z, Borowski L, Mailliard RB, Rolland M, Mullins JI, et al. Dendritic cells reveal a broad range of MHC class I epitopes for HIV-1 in persons with suppressed viral load on antiretroviral therapy. PloS one. 2010;5(9):e12936.

18. Turnbull EL, Lopes AR, Jones NA, Cornforth D, Newton P, Aldam D, et al. HIV-1 epitope-specific CD8+ T cell responses strongly associated with delayed disease progression cross-recognize epitope variants efficiently. Journal of immunology (Baltimore, Md : 1950). 2006;176(10):6130-46.

19. Chopera DR, Woodman Z, Mlisana K, Mlotshwa M, Martin DP, Seoighe C, et al. Transmission of HIV-1 CTL escape variants provides HLA-mismatched recipients with a survival advantage. PLoS pathogens. 2008;4(3):e1000033.

20. Kiepiela P, Leslie AJ, Honeyborne I, Ramduth D, Thobakgale C, Chetty S, et al. Dominant influence of HLA-B in mediating the potential co-evolution of HIV and HLA. Nature. 2004;432(7018):769-75.

21. Gray CM, Mlotshwa M, Riou C, Mathebula T, de Assis Rosa D, Mashishi T, et al. Human immunodeficiency virus-specific gamma interferon enzyme-linked immunospot assay responses targeting specific regions of the proteome during primary subtype C infection are poor predictors of the course of viremia and set point. Journal of virology. 2009;83(1):470-8.

22. Mlotshwa M, Riou C, Chopera D, de Assis Rosa D, Ntale R, Treunicht F, et al. Fluidity of HIV-1-specific T-cell responses during acute and early subtype C HIV-1 infection and associations with early disease progression. Journal of virology. 2010;84(22):12018-29.

23. Geldmacher C, Currier JR, Gerhardt M, Haule A, Maboko L, Birx D, et al. In a mixed subtype epidemic, the HIV-1 Gag-specific T-cell response is biased towards the infecting subtype. AIDS (London, England). 2007;21(2):135-43.

24. Thakar MR, Bhonge LS, Lakhashe SK, Shankarkumar U, Sane SS, Kulkarni SS, et al. Cytolytic T lymphocytes (CTLs) from HIV-1 subtype C-infected Indian patients recognize CTL epitopes from a conserved immunodominant region of HIV-1 Gag and Nef. The Journal of infectious diseases. 2005;192(5):749-59.

25. Masemola AM, Mashishi TN, Khoury G, Bredell H, Paximadis M, Mathebula T, et al. Novel and promiscuous CTL epitopes in conserved regions of Gag targeted by individuals with early subtype C HIV type 1 infection from southern Africa. Journal of immunology (Baltimore, Md : 1950). 2004;173(7):4607-17.

26. Kloverpris HN, Stryhn A, Harndahl M, van der Stok M, Payne RP, Matthews PC, et al. HLA-B*57 Micropolymorphism shapes HLA allele-specific epitope immunogenicity, selection pressure, and HIV immune control. Journal of virology. 2012;86(2):919-29.

27. Smith KN, Mailliard RB, Piazza PA, Fischer W, Korber BT, Fecek RJ, et al. Effective Cytotoxic T Lymphocyte Targeting of Persistent HIV-1 during Antiretroviral Therapy Requires Priming of Naive CD8+ T Cells. mBio. 2016;7(3).

28. Westrop SJ, Grageda N, Imami N. Novel approach to recognition of predicted HIV-1 Gag B3501-restricted CD8 T-cell epitopes by HLA-B3501(+) patients: confirmation by quantitative ELISpot analyses and characterisation using multimers. Journal of immunological methods. 2009;341(1-2):76-85.

29. Goulder PJ, Edwards A, Phillips RE, McMichael AJ. Identification of a novel HLA-B*3501-restricted cytotoxic T lymphocyte epitope using overlapping peptides. AIDS (London, England). 1997;11(7):930-2.

30. Novitsky V, Rybak N, McLane MF, Gilbert P, Chigwedere P, Klein I, et al. Identification of human immunodeficiency virus type 1 subtype C Gag-, Tat-, Rev-, and Nef-specific elispot-based cytotoxic T-lymphocyte responses for AIDS vaccine design. Journal of virology. 2001;75(19):9210-28.

31. Goulder PJ, Tang Y, Brander C, Betts MR, Altfeld M, Annamalai K, et al. Functionally inert HIV-specific cytotoxic T lymphocytes do not play a major role in chronically infected adults and children. The Journal of experimental medicine. 2000;192(12):1819-32.

32. Tenzer S, Wee E, Burgevin A, Stewart-Jones G, Friis L, Lamberth K, et al. Antigen processing influences HIV-specific cytotoxic T lymphocyte immunodominance. Nature immunology. 2009;10(6):636-46.

33. Mahlokozera T, Kang HH, Goonetilleke N, Stacey AR, Lovingood RV, Denny TN, et al. The magnitude and kinetics of the mucosal HIV-specific CD8+ T lymphocyte response and virus RNA load in breast milk. PloS one. 2011;6(8):e23735.

34. Altfeld MA, Livingston B, Reshamwala N, Nguyen PT, Addo MM, Shea A, et al. Identification of novel HLA-A2-restricted human immunodeficiency virus type 1-specific cytotoxic T-lymphocyte epitopes predicted by the HLA-A2 supertype peptide-binding motif. Journal of virology. 2001;75(3):1301-11.

35. Asquith B, Edwards CT, Lipsitch M, McLean AR. Inefficient cytotoxic T lymphocyte-mediated killing of HIV-1-infected cells in vivo. PLoS Biol. 2006;4(4):e90.

36. Bansal A, Gough E, Sabbaj S, Ritter D, Yusim K, Sfakianos G, et al. CD8 T-cell responses in early HIV-1 infection are skewed towards high entropy peptides. Aids. 2005;19(3):241-50.

37. Bennett MS, Joseph A, Ng HL, Goldstein H, Yang OO. Fine-tuning of T-cell receptor avidity to increase HIV epitope variant recognition by cytotoxic T lymphocytes. AIDS (London, England). 2010;24(17):2619-28.

38. Boggiano C, Moya R, Pinilla C, Bihl F, Brander C, Sidney J, et al. Discovery and characterization of highly immunogenic and broadly recognized mimics of the HIV-1 CTL epitope Gag77-85. Eur J Immunol. 2005;35(5):1428-37.

39. Brockman MA, Kwon DS, Tighe DP, Pavlik DF, Rosato PC, Sela J, et al. IL-10 is up-regulated in multiple cell types during viremic HIV infection and reversibly inhibits virus-specific T cells. Blood. 2009;114(2):346-56.

40. Cao J, McNevin J, Holte S, Fink L, Corey L, McElrath MJ. Comprehensive analysis of human immunodeficiency virus type 1 (HIV-1)-specific gamma interferon-secreting CD8+ T cells in primary HIV-1 infection. Journal of virology. 2003;77(12):6867-78.

41. Cella M, Presti R, Vermi W, Lavender K, Turnbull E, Ochsenbauer-Jambor C, et al. Loss of DNAM-1 contributes to CD8+ T-cell exhaustion in chronic HIV-1 infection. Eur J Immunol. 2010;40(4):949-54.

42. Cole DK, Fuller A, Dolton G, Zervoudi E, Legut M, Miles K, et al. Dual Molecular Mechanisms Govern Escape at Immunodominant HLA A2-Restricted HIV Epitope. Frontiers in immunology. 2017;8:1503.

43. Currier JR, Visawapoka U, Tovanabutra S, Mason CJ, Birx DL, McCutchan FE, et al. CTL epitope distribution patterns in the Gag and Nef proteins of HIV-1 from subtype A infected subjects in Kenya: use of multiple peptide sets increases the detectable breadth of the CTL response. BMC Immunol. 2006;7:8.

44. Dagarag M, Ng H, Lubong R, Effros RB, Yang OO. Differential impairment of lytic and cytokine functions in senescent human immunodeficiency virus type 1-specific cytotoxic T lymphocytes. Journal of virology. 2003;77(5):3077-83.

45. Ding Y, Liu J, Lu S, Igweze J, Xu W, Kuang D, et al. Self-assembling peptide for co-delivery of HIV-1 CD8+ T cells epitope and Toll-like receptor 7/8 agonists R848 to induce maturation of monocyte derived dendritic cell and augment polyfunctional cytotoxic T lymphocyte (CTL) response. J Control Release. 2016;236:22-30.

46. Dupuis M, Peshwa MV, Benike C, Kundu SK, Engleman EG, Van Schooten WC, et al. Allogeneic dendritic cell induction of HIV-specific cytotoxic T lymphocyte responses from T cells of HIV type 1-infected and uninfected individuals. AIDS research and human retroviruses. 1997;13(1):33-9.

47. Lin Z, Kuroki K, Kuse N, Sun X, Akahoshi T, Qi Y, et al. HIV-1 Control by NK Cells via Reduced Interaction between KIR2DL2 and HLA-C(∗)12:02/C(∗)14:03. Cell Rep. 2016;17(9):2210-20.

48. Ndhlovu ZM, Piechocka-Trocha A, Vine S, McMullen A, Koofhethile KC, Goulder PJ, et al. Mosaic HIV-1 Gag antigens can be processed and presented to human HIV-specific CD8+ T cells. J Immunol. 2011;186(12):6914-24.

49. Draenert R, Allen TM, Liu Y, Wrin T, Chappey C, Verrill CL, et al. Constraints on HIV-1 evolution and immunodominance revealed in monozygotic adult twins infected with the same virus. J Exp Med. 2006;203(3):529-39.

50. Luo M, Daniuk CA, Diallo TO, Capina RE, Kimani J, Wachihi C, et al. For protection from HIV-1 infection, more might not be better: a systematic analysis of HIV Gag epitopes of two alleles associated with different outcomes of HIV-1 infection. Journal of virology. 2012;86(2):1166-80.

51. Shahid A, Olvera A, Anmole G, Kuang XT, Cotton LA, Plana M, et al. Consequences of HLA-B*13-Associated Escape Mutations on HIV-1 Replication and Nef Function. Journal of virology. 2015;89(22):11557-71.

52. Liu Y, McNevin J, Cao J, Zhao H, Genowati I, Wong K, et al. Selection on the human immunodeficiency virus type 1 proteome following primary infection. Journal of virology. 2006;80(19):9519-29.

53. Liu Y, McNevin JP, Holte S, McElrath MJ, Mullins JI. Dynamics of viral evolution and CTL responses in HIV-1 infection. PloS one. 2011;6(1):e15639.

54. Liu Y, McNevin J, Zhao H, Tebit DM, Troyer RM, McSweyn M, et al. Evolution of human immunodeficiency virus type 1 cytotoxic T-lymphocyte epitopes: fitness-balanced escape. Journal of virology. 2007;81(22):12179-88.

55. Allen TM, Altfeld M, Geer SC, Kalife ET, Moore C, O'Sullivan K M, et al. Selective escape from CD8+ T-cell responses represents a major driving force of human immunodeficiency virus type 1 (HIV-1) sequence diversity and reveals constraints on HIV-1 evolution. Journal of virology. 2005;79(21):13239-49.

56. Boutwell CL, Rowley CF, Essex M. Reduced viral replication capacity of human immunodeficiency virus type 1 subtype C caused by cytotoxic-T-lymphocyte escape mutations in HLA-B57 epitopes of capsid protein. J Virol. 2009;83(6):2460-8.

57. Brackenridge S, Evans EJ, Toebes M, Goonetilleke N, Liu MK, di Gleria K, et al. An early HIV mutation within an HLA-B*57-restricted T cell epitope abrogates binding to the killer inhibitory receptor 3DL1. J Virol. 2011;85(11):5415-22.

58. Fadda L, O'Connor GM, Kumar S, Piechocka-Trocha A, Gardiner CM, Carrington M, et al. Common HIV-1 peptide variants mediate differential binding of KIR3DL1 to HLA-Bw4 molecules. Journal of virology. 2011;85(12):5970-4.

59. Feeney ME, Tang Y, Pfafferott K, Roosevelt KA, Draenert R, Trocha A, et al. HIV-1 viral escape in infancy followed by emergence of a variant-specific CTL response. Journal of immunology (Baltimore, Md : 1950). 2005;174(12):7524-30.

60. Ferrari G, Korber B, Goonetilleke N, Liu MK, Turnbull EL, Salazar-Gonzalez JF, et al. Relationship between functional profile of HIV-1 specific CD8 T cells and epitope variability with the selection of escape mutants in acute HIV-1 infection. PLoS pathogens. 2011;7(2):e1001273.

61. Gillespie GM, Bashirova A, Dong T, McVicar DW, Rowland-Jones SL, Carrington M. Lack of KIR3DS1 binding to MHC class I Bw4 tetramers in complex with CD8+ T cell epitopes. AIDS research and human retroviruses. 2007;23(3):451-5.

62. Goonetilleke N, Liu MK, Salazar-Gonzalez JF, Ferrari G, Giorgi E, Ganusov VV, et al. The first T cell response to transmitted/founder virus contributes to the control of acute viremia in HIV-1 infection. The Journal of experimental medicine. 2009;206(6):1253-72.

63. Goulder PJ, Bunce M, Krausa P, McIntyre K, Crowley S, Morgan B, et al. Novel, cross-restricted, conserved, and immunodominant cytotoxic T lymphocyte epitopes in slow progressors in HIV type 1 infection. AIDS Res Hum Retroviruses. 1996;12(18):1691-8.

64. Reche PA, Keskin DB, Hussey RE, Ancuta P, Gabuzda D, Reinherz EL. Elicitation from virus-naive individuals of cytotoxic T lymphocytes directed against conserved HIV-1 epitopes. Med Immunol. 2006;5:1.

65. Melhem NM, Smith KN, Huang XL, Colleton BA, Jiang W, Mailliard RB, et al. The impact of viral evolution and frequency of variant epitopes on primary and memory human immunodeficiency virus type 1-specific CD8⁺ T cell responses. Virology. 2014;450-451:34-48.

66. Kløverpris HN, McGregor R, McLaren JE, Ladell K, Harndahl M, Stryhn A, et al. CD8+ TCR Bias and Immunodominance in HIV-1 Infection. J Immunol. 2015;194(11):5329-45.

67. Erup Larsen M, Kloverpris H, Stryhn A, Koofhethile CK, Sims S, Ndung'u T, et al. HLArestrictor--a tool for patient-specific predictions of HLA restriction elements and optimal epitopes within peptides. Immunogenetics. 2011;63(1):43-55.

68. Frahm N, Kiepiela P, Adams S, Linde CH, Hewitt HS, Sango K, et al. Control of human immunodeficiency virus replication by cytotoxic T lymphocytes targeting subdominant epitopes. Nature immunology. 2006;7(2):173-8.

69. Radebe M, Nair K, Chonco F, Bishop K, Wright JK, van der Stok M, et al. Limited immunogenicity of HIV CD8+ T-cell epitopes in acute Clade C virus infection. J Infect Dis. 2011;204(5):768-76.

70. Gudmundsdotter L, Bernasconi D, Hejdeman B, Sandstrom E, Alaeus A, Lidman K, et al. Cross-clade immune responses to Gag p24 in patients infected with different HIV-1 subtypes and correlation with HLA class I and II alleles. Vaccine. 2008;26(40):5182-7.

71. Stewart-Jones GB, Gillespie G, Overton IM, Kaul R, Roche P, McMichael AJ, et al. Structures of three HIV-1 HLA-B*5703-peptide complexes and identification of related HLAs potentially associated with long-term nonprogression. J Immunol. 2005;175(4):2459-68.

72. Chopera DR, Mlotshwa M, Woodman Z, Mlisana K, de Assis Rosa D, Martin DP, et al. Virological and immunological factors associated with HIV-1 differential disease progression in HLA-B 58:01-positive individuals. Journal of virology. 2011;85(14):7070-80.

73. Currier JR, Harris ME, Cox JH, McCutchan FE, Birx DL, Maayan S, et al. Immunodominance and cross-reactivity of B5703-restricted CD8 T lymphocytes from HIV type 1 subtype C-infected Ethiopians. AIDS Res Hum Retroviruses. 2005;21(3):239-45.

74. Dinges WL, Richardt J, Friedrich D, Jalbert E, Liu Y, Stevens CE, et al. Virus-specific CD8+ T-cell responses better define HIV disease progression than HLA genotype. J Virol. 2010;84(9):4461-8.

75. Goonetilleke N, Moore S, Dally L, Winstone N, Cebere I, Mahmoud A, et al. Induction of multifunctional human immunodeficiency virus type 1 (HIV-1)-specific T cells capable of proliferation in healthy subjects by using a prime-boost regimen of DNA- and modified vaccinia virus Ankara-vectored vaccines expressing HIV-1 Gag coupled to CD8+ T-cell epitopes. J Virol. 2006;80(10):4717-28.

76. Goulder P, Conlon C, McLntyre K, McMichael A. Identification of a novel human leukocyte antigen A26-restricted epitope in a conserved region of Gag. Aids. 1996;10(12):1441-3.

77. Kawashima Y, Pfafferott K, Frater J, Matthews P, Payne R, Addo M, et al. Adaptation of HIV-1 to human leukocyte antigen class I. Nature. 2009;458(7238):641-5.

78. Kløverpris HN, Payne RP, Sacha JB, Rasaiyaah JT, Chen F, Takiguchi M, et al. Early antigen presentation of protective HIV-1 KF11Gag and KK10Gag epitopes from incoming viral particles facilitates rapid recognition of infected cells by specific CD8+ T cells. Journal of virology. 2013;87(5):2628-38.

79. Lécuroux C, Sáez-Cirión A, Girault I, Versmisse P, Boufassa F, Avettand-Fenoël V, et al. Both HLA-B*57 and plasma HIV RNA levels contribute to the HIV-specific CD8+ T cell response in HIV controllers. Journal of virology. 2014;88(1):176-87.

80. Hulsmans M, Sager HB, Roh JD, Valero-Muñoz M, Houstis NE, Iwamoto Y, et al. Cardiac macrophages promote diastolic dysfunction. The Journal of experimental medicine. 2018;215(2):423-40.

81. Matthews PC, Prendergast A, Leslie A, Crawford H, Payne R, Rousseau C, et al. Central role of reverting mutations in HLA associations with human immunodeficiency virus set point. Journal of virology. 2008;82(17):8548-59.

82. Migueles SA, Laborico AC, Imamichi H, Shupert WL, Royce C, McLaughlin M, et al. The differential ability of HLA B*5701+ long-term nonprogressors and progressors to restrict human immunodeficiency virus replication is not caused by loss of recognition of autologous viral gag sequences. J Virol. 2003;77(12):6889-98.

83. Mkhwanazi N, Thobakgale CF, van der Stok M, Reddy S, Mncube Z, Chonco F, et al. Immunodominant HIV-1-specific HLA-B- and HLA-C-restricted CD8+ T cells do not differ in polyfunctionality. Virology. 2010;405(2):483-91.

84. Buggert M, Norström MM, Czarnecki C, Tupin E, Luo M, Gyllensten K, et al. Characterization of HIV-specific CD4+ T cell responses against peptides selected with broad population and pathogen coverage. PloS one. 2012;7(7):e39874.

85. O'Connell KA, Brennan TP, Bailey JR, Ray SC, Siliciano RF, Blankson JN. Control of HIV-1 in elite suppressors despite ongoing replication and evolution in plasma virus. J Virol. 2010;84(14):7018-28.

86. Payne RP, Branch S, Kløverpris H, Matthews PC, Koofhethile CK, Strong T, et al. Differential escape patterns within the dominant HLA-B*57:03-restricted HIV Gag epitope reflect distinct clade-specific functional constraints. Journal of virology. 2014;88(9):4668-78.

87. Simons BC, Vancompernolle SE, Smith RM, Wei J, Barnett L, Lorey SL, et al. Despite biased TRBV gene usage against a dominant HLA B57-restricted epitope, TCR diversity can provide recognition of circulating epitope variants. J Immunol. 2008;181(7):5137-46.

88. Zhang Z, Zhao QX, Fu JL, Yao JX, He Y, Jin L, et al. Characteristics of HIV-1-specific CD8 T-cell responses and their role in loss of viremia in children chronically infected with HIV-1 undergoing highly active antiretroviral therapy. Chin Med J (Engl). 2006;119(23):1949-57.

89. Kawashima Y, Satoh M, Oka S, Takiguchi M. Identification and characterization of HIV-1 epitopes presented by HLA-A*2603: comparison between HIV-1 epitopes presented by A*2601 and A*2603. Hum Immunol. 2005;66(11):1155-66.

90. Satoh M, Takamiya Y, Oka S, Tokunaga K, Takiguchi M. Identification and characterization of HIV-1-specific CD8+ T cell epitopes presented by HLA-A*2601. Vaccine. 2005;23(29):3783-90.

91. Yamada N, Ishikawa Y, Dumrese T, Tokunaga K, Juji T, Nagatani T, et al. Role of anchor residues in peptide binding to three HLA-A26 molecules. Tissue Antigens. 1999;54(4):325-32.

92. Fujiwara M, Tanuma J, Koizumi H, Kawashima Y, Honda K, Mastuoka-Aizawa S, et al. Different abilities of escape mutant-specific cytotoxic T cells to suppress replication of escape mutant and wild-type human immunodeficiency virus type 1 in new hosts. Journal of virology. 2008;82(1):138-47.

93. Stoll A, Bergmann S, Mummert C, Mueller-Schmucker SM, Spriewald BM, Harrer EG, et al. Identification of HLA-C restricted, HIV-1-specific CTL epitopes by peptide induced upregulation of HLA-C expression. Journal of immunological methods. 2015;418:9-18.

94. Goulder P, Price D, Nowak M, Rowland-Jones S, Phillips R, McMichael A. Co-evolution of human immunodeficiency virus and cytotoxic T-lymphocyte responses. Immunol Rev. 1997;159:17-29.

95. Blais ME, Zhang Y, Rostron T, Griffin H, Taylor S, Xu K, et al. High frequency of HIV mutations associated with HLA-C suggests enhanced HLA-C-restricted CTL selective pressure associated with an AIDS-protective polymorphism. Journal of immunology (Baltimore, Md : 1950). 2012;188(9):4663-70.

96. Honeyborne I, Rathod A, Buchli R, Ramduth D, Moodley E, Rathnavalu P, et al. Motif inference reveals optimal CTL epitopes presented by HLA class I alleles highly prevalent in southern Africa. Journal of immunology (Baltimore, Md : 1950). 2006;176(8):4699-705.

97. Ntale RS, Chopera DR, Ngandu NK, Assis de Rosa D, Zembe L, Gamieldien H, et al. Temporal association of HLA-B*81:01- and HLA-B*39:10-mediated HIV-1 p24 sequence evolution with disease progression. J Virol. 2012;86(22):12013-24.

98. van Baalen CA, Klein MR, Huisman RC, Dings ME, Kerkhof Garde SR, Geretti AM, et al. Fine-specificity of cytotoxic T lymphocytes which recognize conserved epitopes of the Gag protein of human immunodeficiency virus type 1. J Gen Virol. 1996;77 ( Pt 8):1659-65.

99. Watanabe K, Murakoshi H, Tamura Y, Koyanagi M, Chikata T, Gatanaga H, et al. Identification of cross-clade CTL epitopes in HIV-1 clade A/E-infected individuals by using the clade B overlapping peptides. Microbes and infection. 2013;15(13):874-86.

100. Barton JP, Goonetilleke N, Butler TC, Walker BD, McMichael AJ, Chakraborty AK. Relative rate and location of intra-host HIV evolution to evade cellular immunity are predictable. Nat Commun. 2016;7:11660.

101. Bhattacharya T, Daniels M, Heckerman D, Foley B, Frahm N, Kadie C, et al. Founder effects in the assessment of HIV polymorphisms and HLA allele associations. Science (New York, NY). 2007;315(5818):1583-6.

102. Carlson JM, Listgarten J, Pfeifer N, Tan V, Kadie C, Walker BD, et al. Widespread impact of HLA restriction on immune control and escape pathways of HIV-1. Journal of virology. 2012;86(9):5230-43.

103. Cellerai C, Harari A, Stauss H, Yerly S, Geretti AM, Carroll A, et al. Early and prolonged antiretroviral therapy is associated with an HIV-1-specific T-cell profile comparable to that of long-term non-progressors. PloS one. 2011;6(4):e18164.

104. Larocca TJ, Jeong D, Kohlbrenner E, Lee A, Chen J, Hajjar RJ, et al. CXCR4 gene transfer prevents pressure overload induced heart failure. Journal of molecular and cellular cardiology. 2012;53(2):223-32.

105. Gillespie GM, Pinheiro S, Sayeid-Al-Jamee M, Alabi A, Kaye S, Sabally S, et al. CD8+ T cell responses to human immunodeficiency viruses type 2 (HIV-2) and type 1 (HIV-1) gag proteins are distinguishable by magnitude and breadth but not cellular phenotype. Eur J Immunol. 2005;35(5):1445-53.

106. Koofhethile CK, Ndhlovu ZM, Thobakgale-Tshabalala C, Prado JG, Ismail N, Mncube Z, et al. CD8+ T Cell Breadth and Ex Vivo Virus Inhibition Capacity Distinguish between Viremic Controllers with and without Protective HLA Class I Alleles. Journal of virology. 2016;90(15):6818-31.

107. Wang M, Pan W, Xu Y, Zhang J, Wan J, Jiang H. Microglia-Mediated Neuroinflammation: A Potential Target for the Treatment of Cardiovascular Diseases. Journal of inflammation research. 2022;15:3083-94.

108. Miura T, Brockman MA, Schneidewind A, Lobritz M, Pereyra F, Rathod A, et al. HLA-B57/B*5801 human immunodeficiency virus type 1 elite controllers select for rare gag variants associated with reduced viral replication capacity and strong cytotoxic T-lymphocyte [corrected] recognition. J Virol. 2009;83(6):2743-55.

109. Murakoshi H, Zou C, Kuse N, Akahoshi T, Chikata T, Gatanaga H, et al. CD8(+) T cells specific for conserved, cross-reactive Gag epitopes with strong ability to suppress HIV-1 replication. Retrovirology. 2018;15(1):46.

110. O'Connell KA, Xu J, Durbin AP, Apuzzo LG, Imteyaz H, Williams TM, et al. HIV-1 evolution following transmission to an HLA-B*5801-positive patient. The Journal of infectious diseases. 2009;200(12):1820-4.

111. Song H, Pavlicek JW, Cai F, Bhattacharya T, Li H, Iyer SS, et al. Impact of immune escape mutations on HIV-1 fitness in the context of the cognate transmitted/founder genome. Retrovirology. 2012;9:89.

112. Tenzer S, Crawford H, Pymm P, Gifford R, Sreenu VB, Weimershaus M, et al. HIV-1 adaptation to antigen processing results in population-level immune evasion and affects subtype diversification. Cell Rep. 2014;7(2):448-63.

113. Murakoshi H, Koyanagi M, Akahoshi T, Chikata T, Kuse N, Gatanaga H, et al. Impact of a single HLA-A*24:02-associated escape mutation on the detrimental effect of HLA-B*35:01 in HIV-1 control. EBioMedicine. 2018;36:103-12.

114. Matthews PC, Koyanagi M, Kløverpris HN, Harndahl M, Stryhn A, Akahoshi T, et al. Differential clade-specific HLA-B*3501 association with HIV-1 disease outcome is linked to immunogenicity of a single Gag epitope. Journal of virology. 2012;86(23):12643-54.

115. Streeck H, Lichterfeld M, Alter G, Meier A, Teigen N, Yassine-Diab B, et al. Recognition of a defined region within p24 gag by CD8+ T cells during primary human immunodeficiency virus type 1 infection in individuals expressing protective HLA class I alleles. J Virol. 2007;81(14):7725-31.

116. Day CL, Kaufmann DE, Kiepiela P, Brown JA, Moodley ES, Reddy S, et al. PD-1 expression on HIV-specific T cells is associated with T-cell exhaustion and disease progression. Nature. 2006;443(7109):350-4.

117. Day CL, Kiepiela P, Leslie AJ, van der Stok M, Nair K, Ismail N, et al. Proliferative capacity of epitope-specific CD8 T-cell responses is inversely related to viral load in chronic human immunodeficiency virus type 1 infection. Journal of virology. 2007;81(1):434-8.

118. Partridge T, Nicastri A, Kliszczak AE, Yindom LM, Kessler BM, Ternette N, et al. Discrimination Between Human Leukocyte Antigen Class I-Bound and Co-Purified HIV-Derived Peptides in Immunopeptidomics Workflows. Frontiers in immunology. 2018;9:912.

119. Loffredo JT, Sidney J, Bean AT, Beal DR, Bardet W, Wahl A, et al. Two MHC class I molecules associated with elite control of immunodeficiency virus replication, Mamu-B*08 and HLA-B*2705, bind peptides with sequence similarity. Journal of immunology (Baltimore, Md : 1950). 2009;182(12):7763-75.

120. Betts MR, Exley B, Price DA, Bansal A, Camacho ZT, Teaberry V, et al. Characterization of functional and phenotypic changes in anti-Gag vaccine-induced T cell responses and their role in protection after HIV-1 infection. Proc Natl Acad Sci U S A. 2005;102(12):4512-7.

121. Almeida JR, Sauce D, Price DA, Papagno L, Shin SY, Moris A, et al. Antigen sensitivity is a major determinant of CD8+ T-cell polyfunctionality and HIV-suppressive activity. Blood. 2009;113(25):6351-60.

122. Payne RP, Kløverpris H, Sacha JB, Brumme Z, Brumme C, Buus S, et al. Efficacious early antiviral activity of HIV Gag- and Pol-specific HLA-B 2705-restricted CD8+ T cells. Journal of virology. 2010;84(20):10543-57.

123. Bihl F, Frahm N, Di Giammarino L, Sidney J, John M, Yusim K, et al. Impact of HLA-B alleles, epitope binding affinity, functional avidity, and viral coinfection on the immunodominance of virus-specific CTL responses. Journal of immunology (Baltimore, Md : 1950). 2006;176(7):4094-101.

124. Friedrich D, Jalbert E, Dinges WL, Sidney J, Sette A, Huang Y, et al. Vaccine-induced HIV-specific CD8+ T cells utilize preferential HLA alleles and target-specific regions of HIV-1. Journal of acquired immune deficiency syndromes (1999). 2011;58(3):248-52.

125. Iglesias MC, Almeida JR, Fastenackels S, van Bockel DJ, Hashimoto M, Venturi V, et al. Escape from highly effective public CD8+ T-cell clonotypes by HIV. Blood. 2011;118(8):2138-49.

126. Ladell K, Hashimoto M, Iglesias MC, Wilmann PG, McLaren JE, Gras S, et al. A molecular basis for the control of preimmune escape variants by HIV-specific CD8+ T cells. Immunity. 2013;38(3):425-36.

127. Xia Z, Chen H, Kang SG, Huynh T, Fang JW, Lamothe PA, et al. The complex and specific pMHC interactions with diverse HIV-1 TCR clonotypes reveal a structural basis for alterations in CTL function. Sci Rep. 2014;4:4087.

128. Flerin NC, Chen H, Glover TD, Lamothe PA, Zheng JH, Fang JW, et al. T-Cell Receptor (TCR) Clonotype-Specific Differences in Inhibitory Activity of HIV-1 Cytotoxic T-Cell Clones Is Not Mediated by TCR Alone. J Virol. 2017;91(6).

129. Gorin AM, Du Y, Liu FY, Zhang TH, Ng HL, Hofmann C, et al. HIV-1 epitopes presented by MHC class I types associated with superior immune containment of viremia have highly constrained fitness landscapes. PLoS Pathog. 2017;13(8):e1006541.

130. Wilson JD, Ogg GS, Allen RL, Davis C, Shaunak S, Downie J, et al. Direct visualization of HIV-1-specific cytotoxic T lymphocytes during primary infection. Aids. 2000;14(3):225-33.

131. Yang Y, Ganusov VV. Defining Kinetic Properties of HIV-Specific CD8⁺ T-Cell Responses in Acute Infection. Microorganisms. 2019;7(3).

132. Jones NA, Wei X, Flower DR, Wong M, Michor F, Saag MS, et al. Determinants of human immunodeficiency virus type 1 escape from the primary CD8+ cytotoxic T lymphocyte response. The Journal of experimental medicine. 2004;200(10):1243-56.

133. Goulder PJ, Brander C, Annamalai K, Mngqundaniso N, Govender U, Tang Y, et al. Differential narrow focusing of immunodominant human immunodeficiency virus gag-specific cytotoxic T-lymphocyte responses in infected African and caucasoid adults and children. J Virol. 2000;74(12):5679-90.

134. Johnson RP, Trocha A, Yang L, Mazzara GP, Panicali DL, Buchanan TM, et al. HIV-1 gag-specific cytotoxic T lymphocytes recognize multiple highly conserved epitopes. Fine specificity of the gag-specific response defined by using unstimulated peripheral blood mononuclear cells and cloned effector cells. J Immunol. 1991;147(5):1512-21.

135. Chikata T, Murakoshi H, Koyanagi M, Honda K, Gatanaga H, Oka S, et al. Control of HIV-1 by an HLA-B*52:01-C*12:02 Protective Haplotype. The Journal of infectious diseases. 2017;216(11):1415-24.

136. Zhang Y, Kuse N, Akahoshi T, Chikata T, Gatanaga H, Oka S, et al. Role of Escape Mutant-Specific T Cells in Suppression of HIV-1 Replication and Coevolution with HIV-1. J Virol. 2020;94(19).

137. Murakoshi H, Akahoshi T, Koyanagi M, Chikata T, Naruto T, Maruyama R, et al. Clinical Control of HIV-1 by Cytotoxic T Cells Specific for Multiple Conserved Epitopes. Journal of virology. 2015;89(10):5330-9.

138. Zhang Y, Murakoshi H, Chikata T, Akahoshi T, Tran GV, Nguyen TV, et al. Effect of Difference in Consensus Sequence between HIV-1 Subtype A/E and Subtype B Viruses on Elicitation of Gag-Specific CD8(+) T Cells and Accumulation of HLA-Associated Escape Mutations. J Virol. 2021;95(6).

139. Ondondo B, Murakoshi H, Clutton G, Abdul-Jawad S, Wee EG, Gatanaga H, et al. Novel Conserved-region T-cell Mosaic Vaccine With High Global HIV-1 Coverage Is Recognized by Protective Responses in Untreated Infection. Molecular therapy : the journal of the American Society of Gene Therapy. 2016;24(4):832-42.

140. Tsai MC, Singh S, Adland E, Goulder P. Impact of HLA-B*52:01-Driven Escape Mutations on Viral Replicative Capacity. J Virol. 2020;94(13).

141. Buranapraditkun S, Hempel U, Pitakpolrat P, Allgaier RL, Thantivorasit P, Lorenzen SI, et al. A novel immunodominant CD8+ T cell response restricted by a common HLA-C allele targets a conserved region of Gag HIV-1 clade CRF01_AE infected Thais. PLoS One. 2011;6(8):e23603.

142. Van Tran G, Chikata T, Carlson JM, Murakoshi H, Nguyen DH, Tamura Y, et al. A strong association of human leukocyte antigen-associated Pol and Gag mutations with clinical parameters in HIV-1 subtype A/E infection. Aids. 2016;30(5):681-9.

143. Lohman-Payne B, Slyker JA, Richardson BA, Farquhar C, Majiwa M, Maleche-Obimbo E, et al. Infants with late breast milk acquisition of HIV-1 generate interferon-gamma responses more rapidly than infants with early peripartum acquisition. Clin Exp Immunol. 2009;156(3):511-7.

144. Bansal A, Yue L, Conway J, Yusim K, Tang J, Kappes J, et al. Immunological control of chronic HIV-1 infection: HLA-mediated immune function and viral evolution in adolescents. Aids. 2007;21(18):2387-97.

145. Prado JG, Prendergast A, Thobakgale C, Molina C, Tudor-Williams G, Ndung'u T, et al. Replicative capacity of human immunodeficiency virus type 1 transmitted from mother to child is associated with pediatric disease progression rate. J Virol. 2010;84(1):492-502.

146. Norström MM, Buggert M, Tauriainen J, Hartogensis W, Prosperi MC, Wallet MA, et al. Combination of immune and viral factors distinguishes low-risk versus high-risk HIV-1 disease progression in HLA-B*5701 subjects. J Virol. 2012;86(18):9802-16.

147. Martin MP, Naranbhai V, Shea PR, Qi Y, Ramsuran V, Vince N, et al. Killer cell immunoglobulin-like receptor 3DL1 variation modifies HLA-B*57 protection against HIV-1. J Clin Invest. 2018;128(5):1903-12.

148. Murakoshi H, Kitano M, Akahoshi T, Kawashima Y, Dohki S, Oka S, et al. Identification and characterization of 2 HIV-1 Gag immunodominant epitopes restricted by Asian HLA allele HLA-B*4801. Hum Immunol. 2009;70(3):170-4.

149. Kløverpris HN, Harndahl M, Leslie AJ, Carlson JM, Ismail N, van der Stok M, et al. HIV control through a single nucleotide on the HLA-B locus. Journal of virology. 2012;86(21):11493-500.

150. Keane NM, Roberts SG, Almeida CA, Krishnan T, Chopra A, Demaine E, et al. High-avidity, high-IFNγ-producing CD8 T-cell responses following immune selection during HIV-1 infection. Immunol Cell Biol. 2012;90(2):224-34.

151. Ahmed T, Borthwick NJ, Gilmour J, Hayes P, Dorrell L, Hanke T. Control of HIV-1 replication in vitro by vaccine-induced human CD8(+) T cells through conserved subdominant Pol epitopes. Vaccine. 2016;34(9):1215-24.

152. Yang H, Llano A, Cedeño S, von Delft A, Corcuera A, Gillespie GM, et al. Incoming HIV virion-derived Gag Spacer Peptide 2 (p1) is a target of effective CD8(+) T cell antiviral responses. Cell reports. 2021;35(6):109103.

153. Mwimanzi F, Toyoda M, Mahiti M, Mann JK, Martin JN, Bangsberg D, et al. Resistance of Major Histocompatibility Complex Class B (MHC-B) to Nef-Mediated Downregulation Relative to that of MHC-A Is Conserved among Primate Lentiviruses and Influences Antiviral T Cell Responses in HIV-1-Infected Individuals. J Virol. 2018;92(1).

154. Trautmann L, Mbitikon-Kobo FM, Goulet JP, Peretz Y, Shi Y, Van Grevenynghe J, et al. Profound metabolic, functional, and cytolytic differences characterize HIV-specific CD8 T cells in primary and chronic HIV infection. Blood. 2012;120(17):3466-77.

155. Cao J, McNevin J, McSweyn M, Liu Y, Mullins JI, McElrath MJ. Novel cytotoxic T-lymphocyte escape mutation by a three-amino-acid insertion in the human immunodeficiency virus type 1 p6Pol and p6Gag late domain associated with drug resistance. J Virol. 2008;82(1):495-502.

156. Gorse GJ, Baden LR, Wecker M, Newman MJ, Ferrari G, Weinhold KJ, et al. Safety and immunogenicity of cytotoxic T-lymphocyte poly-epitope, DNA plasmid (EP HIV-1090) vaccine in healthy, human immunodeficiency virus type 1 (HIV-1)-uninfected adults. Vaccine. 2008;26(2):215-23.

157. Kaul R, Rowland-Jones SL, Kimani J, Dong T, Yang HB, Kiama P, et al. Late seroconversion in HIV-resistant Nairobi prostitutes despite pre-existing HIV-specific CD8+ responses. The Journal of clinical investigation. 2001;107(3):341-9.

158. Matthews PC, Adland E, Listgarten J, Leslie A, Mkhwanazi N, Carlson JM, et al. HLA-A*7401-mediated control of HIV viremia is independent of its linkage disequilibrium with HLA-B*5703. Journal of immunology (Baltimore, Md : 1950). 2011;186(10):5675-86.

159. Zhang Y, Chikata T, Kuse N, Murakoshi H, Gatanaga H, Oka S, et al. Immunological Control of HIV-1 Disease Progression by Rare Protective HLA Allele. Journal of virology. 2022;96(22):e0124822.

160. McKinney DM, Skvoretz R, Livingston BD, Wilson CC, Anders M, Chesnut RW, et al. Recognition of variant HIV-1 epitopes from diverse viral subtypes by vaccine-induced CTL. Journal of immunology (Baltimore, Md : 1950). 2004;173(3):1941-50.

161. Stratov I, Dale CJ, Chea S, McCluskey J, Kent SJ. Induction of T-cell immunity to antiretroviral drug-resistant human immunodeficiency virus type 1. J Virol. 2005;79(12):7728-37.

162. Kaul R, Dong T, Plummer FA, Kimani J, Rostron T, Kiama P, et al. CD8(+) lymphocytes respond to different HIV epitopes in seronegative and infected subjects. The Journal of clinical investigation. 2001;107(10):1303-10.

163. Abdul-Jawad S, Ondondo B, van Hateren A, Gardner A, Elliott T, Korber B, et al. Increased Valency of Conserved-mosaic Vaccines Enhances the Breadth and Depth of Epitope Recognition. Mol Ther. 2016;24(2):375-84.

164. Kónya J, Stuber G, Björndal A, Fenyö EM, Dillner J. Primary induction of human cytotoxic lymphocytes against a synthetic peptide of the human immunodeficiency virus type 1 protease. The Journal of general virology. 1997;78 ( Pt 9):2217-24.

165. Kitano M, Kobayashi N, Kawashima Y, Akahoshi T, Nokihara K, Oka S, et al. Identification and characterization of HLA-B*5401-restricted HIV-1-Nef and Pol-specific CTL epitopes. Microbes and infection. 2008;10(7):764-72.

166. Hashimoto M, Kitano M, Honda K, Koizumi H, Dohki S, Oka S, et al. Selection of escape mutation by Pol154-162-specific cytotoxic T cells among chronically HIV-1-infected HLA-B*5401-positive individuals. Human immunology. 2010;71(2):123-7.

167. Klenerman P, Meier UC, Phillips RE, McMichael AJ. The effects of natural altered peptide ligands on the whole blood cytotoxic T lymphocyte response to human immunodeficiency virus. Eur J Immunol. 1995;25(7):1927-31.

168. Meier UC, Klenerman P, Griffin P, James W, Köppe B, Larder B, et al. Cytotoxic T lymphocyte lysis inhibited by viable HIV mutants. Science (New York, NY). 1995;270(5240):1360-2.

169. Walker BD, Flexner C, Birch-Limberger K, Fisher L, Paradis TJ, Aldovini A, et al. Long-term culture and fine specificity of human cytotoxic T-lymphocyte clones reactive with human immunodeficiency virus type 1. Proceedings of the National Academy of Sciences of the United States of America. 1989;86(23):9514-8.

170. Isaguliants MG, Zuber B, Boberg A, Sjöstrand D, Belikov SV, Rollman E, et al. Reverse transcriptase-based DNA vaccines against drug-resistant HIV-1 tested in a mouse model. Vaccine. 2004;22(13-14):1810-9.

171. Lorin C, Delebecque F, Labrousse V, Da Silva L, Lemonnier F, Brahic M, et al. A recombinant live attenuated measles vaccine vector primes effective HLA-A0201-restricted cytotoxic T lymphocytes and broadly neutralizing antibodies against HIV-1 conserved epitopes. Vaccine. 2005;23(36):4463-72.

172. Singh RA, Barry MA. Repertoire and immunofocusing of CD8 T cell responses generated by HIV-1 gag-pol and expression library immunization vaccines. Journal of immunology (Baltimore, Md : 1950). 2004;173(7):4387-93.

173. Zou C, Murakoshi H, Kuse N, Akahoshi T, Chikata T, Gatanaga H, et al. Effective Suppression of HIV-1 Replication by Cytotoxic T Lymphocytes Specific for Pol Epitopes in Conserved Mosaic Vaccine Immunogens. J Virol. 2019;93(7).

174. Borthwick N, Lin Z, Akahoshi T, Llano A, Silva-Arrieta S, Ahmed T, et al. Novel, in-natural-infection subdominant HIV-1 CD8+ T-cell epitopes revealed in human recipients of conserved-region T-cell vaccines. PloS one. 2017;12(4):e0176418.

175. Ueno T, Motozono C, Dohki S, Mwimanzi P, Rauch S, Fackler OT, et al. CTL-mediated selective pressure influences dynamic evolution and pathogenic functions of HIV-1 Nef. Journal of immunology (Baltimore, Md : 1950). 2008;180(2):1107-16.

176. Motozono C, Kuse N, Sun X, Rizkallah PJ, Fuller A, Oka S, et al. Molecular basis of a dominant T cell response to an HIV reverse transcriptase 8-mer epitope presented by the protective allele HLA-B*51:01. Journal of immunology (Baltimore, Md : 1950). 2014;192(7):3428-34.

177. Tomiyama H, Fujiwara M, Oka S, Takiguchi M. Cutting Edge: Epitope-dependent effect of Nef-mediated HLA class I down-regulation on ability of HIV-1-specific CTLs to suppress HIV-1 replication. Journal of immunology (Baltimore, Md : 1950). 2005;174(1):36-40.

178. Kawashima Y, Kuse N, Gatanaga H, Naruto T, Fujiwara M, Dohki S, et al. Long-term control of HIV-1 in hemophiliacs carrying slow-progressing allele HLA-B*5101. Journal of virology. 2010;84(14):7151-60.

179. Migueles SA, Mendoza D, Zimmerman MG, Martins KM, Toulmin SA, Kelly EP, et al. CD8(+) T-cell Cytotoxic Capacity Associated with Human Immunodeficiency Virus-1 Control Can Be Mediated through Various Epitopes and Human Leukocyte Antigen Types. EBioMedicine. 2015;2(1):46-58.

180. Kemal KS, Beattie T, Dong T, Weiser B, Kaul R, Kuiken C, et al. Transition from long-term nonprogression to HIV-1 disease associated with escape from cellular immune control. Journal of acquired immune deficiency syndromes (1999). 2008;48(2):119-26.

181. De Groot AS, Jesdale B, Martin W, Saint Aubin C, Sbai H, Bosma A, et al. Mapping cross-clade HIV-1 vaccine epitopes using a bioinformatics approach. Vaccine. 2003;21(27-30):4486-504.

182. Kuse N, Rahman MA, Murakoshi H, Tran GV, Chikata T, Koyanagi M, et al. Different Effects of Nonnucleoside Reverse Transcriptase Inhibitor Resistance Mutations on Cytotoxic T Lymphocyte Recognition between HIV-1 Subtype B and Subtype A/E Infections. Journal of virology. 2015;89(14):7363-72.

183. Honda K, Zheng N, Murakoshi H, Hashimoto M, Sakai K, Borghan MA, et al. Selection of escape mutant by HLA-C-restricted HIV-1 Pol-specific cytotoxic T lymphocytes carrying strong ability to suppress HIV-1 replication. Eur J Immunol. 2011;41(1):97-106.

184. Frater AJ, Brown H, Oxenius A, Günthard HF, Hirschel B, Robinson N, et al. Effective T-cell responses select human immunodeficiency virus mutants and slow disease progression. J Virol. 2007;81(12):6742-51.

185. Reinis M, Weiser B, Kuiken C, Dong T, Lang D, Nachman S, et al. Genomic analysis of HIV type 1 strains derived from a mother and child pair of long-term nonprogressors. AIDS research and human retroviruses. 2007;23(2):309-15.

186. Vollbrecht T, Eberle J, Roider J, Bühler S, Stirner R, Henrich N, et al. Control of M184V HIV-1 mutants by CD8 T-cell responses. Med Microbiol Immunol. 2012;201(2):201-11.

187. Roider J, Meissner T, Kraut F, Vollbrecht T, Stirner R, Bogner JR, et al. Comparison of experimental fine-mapping to in silico prediction results of HIV-1 epitopes reveals ongoing need for mapping experiments. Immunology. 2014;143(2):193-201.

188. Carlson JM, Du VY, Pfeifer N, Bansal A, Tan VY, Power K, et al. Impact of pre-adapted HIV transmission. Nature medicine. 2016;22(6):606-13.

189. Ueno T, Tomiyama H, Fujiwara M, Oka S, Takiguchi M. HLA class I-restricted recognition of an HIV-derived epitope peptide by a human T cell receptor alpha chain having a Vdelta1 variable segment. Eur J Immunol. 2003;33(10):2910-6.

190. Ueno T, Fujiwara M, Tomiyama H, Onodera M, Takiguchi M. Reconstitution of anti-HIV effector functions of primary human CD8 T lymphocytes by transfer of HIV-specific alphabeta TCR genes. Eur J Immunol. 2004;34(12):3379-88.

191. Ueno T, Tomiyama H, Fujiwara M, Oka S, Takiguchi M. Functionally impaired HIV-specific CD8 T cells show high affinity TCR-ligand interactions. Journal of immunology (Baltimore, Md : 1950). 2004;173(9):5451-7.

192. Shacklett BL, Cox CA, Sandberg JK, Stollman NH, Jacobson MA, Nixon DF. Trafficking of human immunodeficiency virus type 1-specific CD8+ T cells to gut-associated lymphoid tissue during chronic infection. Journal of virology. 2003;77(10):5621-31.

193. Brander C, Goulder PJ, Luzuriaga K, Yang OO, Hartman KE, Jones NG, et al. Persistent HIV-1-specific CTL clonal expansion despite high viral burden post in utero HIV-1 infection. Journal of immunology (Baltimore, Md : 1950). 1999;162(8):4796-800.

194. Jones RB, Ndhlovu LC, Barbour JD, Sheth PM, Jha AR, Long BR, et al. Tim-3 expression defines a novel population of dysfunctional T cells with highly elevated frequencies in progressive HIV-1 infection. The Journal of experimental medicine. 2008;205(12):2763-79.

195. Kan-Mitchell J, Bisikirska B, Wong-Staal F, Schaubert KL, Bajcz M, Bereta M. The HIV-1 HLA-A2-SLYNTVATL is a help-independent CTL epitope. Journal of immunology (Baltimore, Md : 1950). 2004;172(9):5249-61.

196. Daftarian P, Ali S, Sharan R, Lacey SF, La Rosa C, Longmate J, et al. Immunization with Th-CTL fusion peptide and cytosine-phosphate-guanine DNA in transgenic HLA-A2 mice induces recognition of HIV-infected T cells and clears vaccinia virus challenge. Journal of immunology (Baltimore, Md : 1950). 2003;171(8):4028-39.

197. Huang XL, Fan Z, Borowski L, Rinaldo CR. Multiple T-cell responses to human immunodeficiency virus type 1 are enhanced by dendritic cells. Clin Vaccine Immunol. 2009;16(10):1504-16.

198. Murakoshi H, Koyanagi M, Chikata T, Rahman MA, Kuse N, Sakai K, et al. Accumulation of Pol Mutations Selected by HLA-B*52:01-C*12:02 Protective Haplotype-Restricted Cytotoxic T Lymphocytes Causes Low Plasma Viral Load Due to Low Viral Fitness of Mutant Viruses. J Virol. 2017;91(4).

199. Harrer T, Harrer E, Kalams SA, Barbosa P, Trocha A, Johnson RP, et al. Cytotoxic T lymphocytes in asymptomatic long-term nonprogressing HIV-1 infection. Breadth and specificity of the response and relation to in vivo viral quasispecies in a person with prolonged infection and low viral load. Journal of immunology (Baltimore, Md : 1950). 1996;156(7):2616-23.

200. Menéndez-Arias L, Mas A, Domingo E. Cytotoxic T-lymphocyte responses to HIV-1 reverse transcriptase (review). Viral Immunol. 1998;11(4):167-81.

201. Kløverpris HN, Stryhn A, Harndahl M, Carlson JM, Leslie AJ, Chen F, et al. HLA-A*68:02-restricted Gag-specific cytotoxic T lymphocyte responses can drive selection pressure on HIV but are subdominant and ineffective. AIDS (London, England). 2013;27(11):1717-23.

202. Koizumi H, Hashimoto M, Fujiwara M, Murakoshi H, Chikata T, Borghan MA, et al. Different in vivo effects of HIV-1 immunodominant epitope-specific cytotoxic T lymphocytes on selection of escape mutant viruses. Journal of virology. 2010;84(11):5508-19.

203. Murakoshi H, Kuse N, Akahoshi T, Zhang Y, Chikata T, Borghan MA, et al. Broad Recognition of Circulating HIV-1 by HIV-1-Specific Cytotoxic T-Lymphocytes with Strong Ability to Suppress HIV-1 Replication. Journal of virology. 2019;93(1).

204. Watanabe T, Murakoshi H, Gatanaga H, Koyanagi M, Oka S, Takiguchi M. Effective recognition of HIV-1-infected cells by HIV-1 integrase-specific HLA-B∗4002-restricted T cells. Microbes and infection. 2011;13(2):160-6.

205. Arnoczy GS, Ferrari G, Goonetilleke N, Corrah T, Li H, Kuruc J, et al. Massive CD8 T cell response to primary HIV infection in the setting of severe clinical presentation. AIDS research and human retroviruses. 2012;28(8):789-92.

206. Silver ZA, Watkins DI. The role of MHC class I gene products in SIV infection of macaques. Immunogenetics. 2017;69(8-9):511-9.

207. Streeck H, Jolin JS, Qi Y, Yassine-Diab B, Johnson RC, Kwon DS, et al. Human immunodeficiency virus type 1-specific CD8+ T-cell responses during primary infection are major determinants of the viral set point and loss of CD4+ T cells. Journal of virology. 2009;83(15):7641-8.

208. Kamori D, Hasan Z, Ohashi J, Kawana-Tachikawa A, Gatanaga H, Oka S, et al. Identification of two unique naturally occurring Vpr sequence polymorphisms associated with clinical parameters in HIV-1 chronic infection. J Med Virol. 2017;89(1):123-9.

209. Fischer W, Ganusov VV, Giorgi EE, Hraber PT, Keele BF, Leitner T, et al. Transmission of single HIV-1 genomes and dynamics of early immune escape revealed by ultra-deep sequencing. PloS one. 2010;5(8):e12303.

210. Liu MK, Hawkins N, Ritchie AJ, Ganusov VV, Whale V, Brackenridge S, et al. Vertical T cell immunodominance and epitope entropy determine HIV-1 escape. The Journal of clinical investigation. 2013;123(1):380-93.

211. Oxenius A, Price DA, Trkola A, Edwards C, Gostick E, Zhang HT, et al. Loss of viral control in early HIV-1 infection is temporally associated with sequential escape from CD8+ T cell responses and decrease in HIV-1-specific CD4+ and CD8+ T cell frequencies. The Journal of infectious diseases. 2004;190(4):713-21.

212. Gostick E, Cole DK, Hutchinson SL, Wooldridge L, Tafuro S, Laugel B, et al. Functional and biophysical characterization of an HLA-A*6801-restricted HIV-specific T cell receptor. Eur J Immunol. 2007;37(2):479-86.

213. Laugel B, Price DA, Milicic A, Sewell AK. CD8 exerts differential effects on the deployment of cytotoxic T lymphocyte effector functions. Eur J Immunol. 2007;37(4):905-13.

214. Allard SD, de Goede AL, De Keersmaecker B, Heirman C, Lacor P, Osterhaus AD, et al. Sequence evolution and escape from specific immune pressure of an HIV-1 Rev epitope with extensive sequence similarity to human nucleolar protein 6. Tissue Antigens. 2012;79(3):174-85.

215. Ferris RL, Hall C, Sipsas NV, Safrit JT, Trocha A, Koup RA, et al. Processing of HIV-1 envelope glycoprotein for class I-restricted recognition: dependence on TAP1/2 and mechanisms for cytosolic localization. J Immunol. 1999;162(3):1324-32.

216. Hammond SA, Johnson RP, Kalams SA, Walker BD, Takiguchi M, Safrit JT, et al. An epitope-selective, transporter associated with antigen presentation (TAP)-1/2-independent pathway and a more general TAP-1/2-dependent antigen-processing pathway allow recognition of the HIV-1 envelope glycoprotein by CD8+ CTL. J Immunol. 1995;154(11):6140-56.

217. Wood N, Bhattacharya T, Keele BF, Giorgi E, Liu M, Gaschen B, et al. HIV evolution in early infection: selection pressures, patterns of insertion and deletion, and the impact of APOBEC. PLoS Pathog. 2009;5(5):e1000414.

218. Gaiha GD, Rossin EJ, Urbach J, Landeros C, Collins DR, Nwonu C, et al. Structural topology defines protective CD8(+) T cell epitopes in the HIV proteome. Science. 2019;364(6439):480-4.

219. Streeck H, Li B, Poon AF, Schneidewind A, Gladden AD, Power KA, et al. Immune-driven recombination and loss of control after HIV superinfection. The Journal of experimental medicine. 2008;205(8):1789-96.

220. Herbeck JT, Rolland M, Liu Y, McLaughlin S, McNevin J, Zhao H, et al. Demographic processes affect HIV-1 evolution in primary infection before the onset of selective processes. J Virol. 2011;85(15):7523-34.

221. Makadzange AT, Gillespie G, Dong T, Kiama P, Bwayo J, Kimani J, et al. Characterization of an HLA-C-restricted CTL response in chronic HIV infection. Eur J Immunol. 2010;40(4):1036-41.

222. Chikata T, Paes W, Akahoshi T, Partridge T, Murakoshi H, Gatanaga H, et al. Identification of Immunodominant HIV-1 Epitopes Presented by HLA-C*12:02, a Protective Allele, Using an Immunopeptidomics Approach. Journal of virology. 2019;93(17).

223. Ngumbela KC, Day CL, Mncube Z, Nair K, Ramduth D, Thobakgale C, et al. Targeting of a CD8 T cell env epitope presented by HLA-B*5802 is associated with markers of HIV disease progression and lack of selection pressure. AIDS research and human retroviruses. 2008;24(1):72-82.

224. Shankar P, Fabry JA, Fong DM, Lieberman J. Three regions of HIV-1 gp160 contain clusters of immunodominant CTL epitopes. Immunology letters. 1996;52(1):23-30.

225. Ferris RL, Buck C, Hammond SA, Woods AS, Cotter RJ, Takiguchi M, et al. Class I-restricted presentation of an HIV-1 gp41 epitope containing an N-linked glycosylation site. Implications for the mechanism of processing of viral envelope proteins. Journal of immunology (Baltimore, Md : 1950). 1996;156(2):834-40.

226. Wherry EJ, Day CL, Draenert R, Miller JD, Kiepiela P, Woodberry T, et al. HIV-specific CD8 T cells express low levels of IL-7Ralpha: implications for HIV-specific T cell memory. Virology. 2006;353(2):366-73.

227. Dupuis M, Kundu SK, Merigan TC. Characterization of HLA-A 0201-restricted cytotoxic T cell epitopes in conserved regions of the HIV type 1 gp160 protein. Journal of immunology (Baltimore, Md : 1950). 1995;155(4):2232-9.

228. Malhotra U, Li F, Nolin J, Allison M, Zhao H, Mullins JI, et al. Enhanced detection of human immunodeficiency virus type 1 (HIV-1) Nef-specific T cells recognizing multiple variants in early HIV-1 infection. Journal of virology. 2007;81(10):5225-37.

229. Goulder PJ, Reid SW, Price DA, O'Callaghan CA, McMichael AJ, Phillips RE, et al. Combined structural and immunological refinement of HIV-1 HLA-B8-restricted cytotoxic T lymphocyte epitopes. Eur J Immunol. 1997;27(6):1515-21.

230. Leitman EM, Thobakgale CF, Adland E, Ansari MA, Raghwani J, Prendergast AJ, et al. Role of HIV-specific CD8(+) T cells in pediatripc HIV cure strategies after widespread early viral escape. The Journal of experimental medicine. 2017;214(11):3239-61.

231. Motozono C, Yokoyama M, Sato H, Ueno T. Cross-reactivity analysis of T cell receptors specific for overlapping HIV-1 Nef epitopes of different lengths. Microbes and infection. 2014;16(4):320-7.

232. Barton JP, Rajkoomar E, Mann JK, Murakowski DK, Toyoda M, Mahiti M, et al. Modelling and in vitro testing of the HIV-1 Nef fitness landscape. Virus Evol. 2019;5(2):vez029.

233. Mwimanzi P, Hasan Z, Tokunaga M, Gatanaga H, Oka S, Ueno T. Naturally arising HIV-1 Nef variants conferring escape from cytotoxic T lymphocytes influence viral entry co-receptor expression and susceptibility to superinfection. Biochemical and biophysical research communications. 2010;403(3-4):422-7.

234. Yaciuk JC, Skaley M, Bardet W, Schafer F, Mojsilovic D, Cate S, et al. Direct interrogation of viral peptides presented by the class I HLA of HIV-infected T cells. Journal of virology. 2014;88(22):12992-3004.

235. Navis M, Schellens IM, van Swieten P, Borghans JA, Miedema F, Kootstra NA, et al. A nonprogressive clinical course in HIV-infected individuals expressing human leukocyte antigen B57/5801 is associated with preserved CD8+ T lymphocyte responsiveness to the HW9 epitope in Nef. The Journal of infectious diseases. 2008;197(6):871-9.

236. Leslie A, Kavanagh D, Honeyborne I, Pfafferott K, Edwards C, Pillay T, et al. Transmission and accumulation of CTL escape variants drive negative associations between HIV polymorphisms and HLA. The Journal of experimental medicine. 2005;201(6):891-902.

237. Claiborne DT, Dudek TE, Maldini CR, Power KA, Ghebremichael M, Seung E, et al. Immunization of BLT Humanized Mice Redirects T Cell Responses to Gag and Reduces Acute HIV-1 Viremia. Journal of virology. 2019;93(20).

238. Dong T, Stewart-Jones G, Chen N, Easterbrook P, Xu X, Papagno L, et al. HIV-specific cytotoxic T cells from long-term survivors select a unique T cell receptor. The Journal of experimental medicine. 2004;200(12):1547-57.

239. Culmann-Penciolelli B, Lamhamedi-Cherradi S, Couillin I, Guegan N, Levy JP, Guillet JG, et al. Identification of multirestricted immunodominant regions recognized by cytolytic T lymphocytes in the human immunodeficiency virus type 1 Nef protein. Journal of virology. 1994;68(11):7336-43.

240. Price DA, Goulder PJ, Klenerman P, Sewell AK, Easterbrook PJ, Troop M, et al. Positive selection of HIV-1 cytotoxic T lymphocyte escape variants during primary infection. Proceedings of the National Academy of Sciences of the United States of America. 1997;94(5):1890-5.

241. Spiegel HM, DeFalcon E, Ogg GS, Larsson M, Beadle TJ, Tao P, et al. Changes in frequency of HIV-1-specific cytotoxic T cell precursors and circulating effectors after combination antiretroviral therapy in children. The Journal of infectious diseases. 1999;180(2):359-68.

242. Adland E, Carlson JM, Paioni P, Kløverpris H, Shapiro R, Ogwu A, et al. Nef-specific CD8+ T cell responses contribute to HIV-1 immune control. PloS one. 2013;8(9):e73117.

243. Frahm N, Yusim K, Suscovich TJ, Adams S, Sidney J, Hraber P, et al. Extensive HLA class I allele promiscuity among viral CTL epitopes. Eur J Immunol. 2007;37(9):2419-33.

244. Karlsson I, Kløverpris H, Jensen KJ, Stryhn A, Buus S, Karlsson A, et al. Identification of conserved subdominant HIV Type 1 CD8(+) T Cell epitopes restricted within common HLA Supertypes for therapeutic HIV Type 1 vaccines. AIDS research and human retroviruses. 2012;28(11):1434-43.

245. Inwoley A, Recordon-Pinson P, Dupuis M, Gaston J, Genête M, Minga A, et al. Cross-clade conservation of HIV type 1 Nef immunodominant regions recognized by CD8+ T cells of HIV type 1 CRF02_AG-infected Ivorian (West Africa). AIDS research and human retroviruses. 2005;21(7):620-8.

246. Han C, Kawana-Tachikawa A, Shimizu A, Zhu D, Nakamura H, Adachi E, et al. Switching and emergence of CTL epitopes in HIV-1 infection. Retrovirology. 2014;11:38.

247. Pillay T, Zhang HT, Drijfhout JW, Robinson N, Brown H, Khan M, et al. Unique acquisition of cytotoxic T-lymphocyte escape mutants in infant human immunodeficiency virus type 1 infection. Journal of virology. 2005;79(18):12100-5.

248. Malhotra U, Nolin J, Horton H, Li F, Corey L, Mullins JI, et al. Functional properties and epitope characteristics of T-cells recognizing natural HIV-1 variants. Vaccine. 2009;27(48):6678-87.

249. Sun X, Shi Y, Akahoshi T, Fujiwara M, Gatanaga H, Schönbach C, et al. Effects of a Single Escape Mutation on T Cell and HIV-1 Co-adaptation. Cell reports. 2016;15(10):2279-91.

250. Sun X, Fujiwara M, Shi Y, Kuse N, Gatanaga H, Appay V, et al. Superimposed epitopes restricted by the same HLA molecule drive distinct HIV-specific CD8+ T cell repertoires. Journal of immunology (Baltimore, Md : 1950). 2014;193(1):77-84.

251. Katoh J, Kawana-Tachikawa A, Shimizu A, Zhu D, Han C, Nakamura H, et al. Rapid HIV-1 Disease Progression in Individuals Infected with a Virus Adapted to Its Host Population. PloS one. 2016;11(3):e0150397.

252. Furutsuki T, Hosoya N, Kawana-Tachikawa A, Tomizawa M, Odawara T, Goto M, et al. Frequent transmission of cytotoxic-T-lymphocyte escape mutants of human immunodeficiency virus type 1 in the highly HLA-A24-positive Japanese population. Journal of virology. 2004;78(16):8437-45.

253. Miyazaki E, Kawana-Tachikawa A, Tomizawa M, Nunoya J, Odawara T, Fujii T, et al. Highly restricted T-cell receptor repertoire in the CD8+ T-cell response against an HIV-1 epitope with a stereotypic amino acid substitution. AIDS (London, England). 2009;23(6):651-60.

254. Figueiredo S, Charmeteau B, Surenaud M, Salmon D, Launay O, Guillet JG, et al. Memory CD8(+) T cells elicited by HIV-1 lipopeptide vaccines display similar phenotypic profiles but differences in term of magnitude and multifunctionality compared with FLU- or EBV-specific memory T cells in humans. Vaccine. 2014;32(4):492-501.

255. Culmann B, Gomard E, Kiény MP, Guy B, Dreyfus F, Saimot AG, et al. Six epitopes reacting with human cytotoxic CD8+ T cells in the central region of the HIV-1 NEF protein. Journal of immunology (Baltimore, Md : 1950). 1991;146(5):1560-5.

256. Chandwani R, Jordan KA, Shacklett BL, Papasavvas E, Montaner LJ, Rosenberg MG, et al. Limited magnitude and breadth in the HLA-A2-restricted CD8 T-Cell response to Nef in children with vertically acquired HIV-1 infection. Scand J Immunol. 2004;59(1):109-14.

257. Corbet S, Nielsen HV, Vinner L, Lauemoller S, Therrien D, Tang S, et al. Optimization and immune recognition of multiple novel conserved HLA-A2, human immunodeficiency virus type 1-specific CTL epitopes. J Gen Virol. 2003;84(Pt 9):2409-21.

258. Frahm N, Adams S, Kiepiela P, Linde CH, Hewitt HS, Lichterfeld M, et al. HLA-B63 presents HLA-B57/B58-restricted cytotoxic T-lymphocyte epitopes and is associated with low human immunodeficiency virus load. Journal of virology. 2005;79(16):10218-25.

259. Gahéry-Ségard H, Pialoux G, Figueiredo S, Igéa C, Surenaud M, Gaston J, et al. Long-term specific immune responses induced in humans by a human immunodeficiency virus type 1 lipopeptide vaccine: characterization of CD8+-T-cell epitopes recognized. Journal of virology. 2003;77(20):11220-31.

260. Karlsson AC, Iversen AK, Chapman JM, de Oliviera T, Spotts G, McMichael AJ, et al. Sequential broadening of CTL responses in early HIV-1 infection is associated with viral escape. PloS one. 2007;2(2):e225.

261. Wilson CC, McKinney D, Anders M, MaWhinney S, Forster J, Crimi C, et al. Development of a DNA vaccine designed to induce cytotoxic T lymphocyte responses to multiple conserved epitopes in HIV-1. Journal of immunology (Baltimore, Md : 1950). 2003;171(10):5611-23.

262. Altfeld M, Kalife ET, Qi Y, Streeck H, Lichterfeld M, Johnston MN, et al. HLA Alleles Associated with Delayed Progression to AIDS Contribute Strongly to the Initial CD8(+) T Cell Response against HIV-1. PLoS Med. 2006;3(10):e403.

263. Lazaro E, Theodorou I, Legrand E, Recordon-Pinson P, Boucher S, Capoulade C, et al. Sequences of clustered epitopes in Gag and Nef potentially presented by predominant class I human leukocyte antigen (HLA) alleles A and B expressed by human immunodeficiency virus type 1 (HIV-1)-infected patients in Vietnam. AIDS research and human retroviruses. 2005;21(6):586-91.

264. Maurer K, Harrer EG, Goldwich A, Eismann K, Bergmann S, Schmitt-Haendle M, et al. Role of cytotoxic T-lymphocyte-mediated immune selection in a dominant human leukocyte antigen-B8-restricted cytotoxic T-lymphocyte epitope in Nef. Journal of acquired immune deficiency syndromes (1999). 2008;48(2):133-41.

265. Plana M, Garcia F, Oxenius A, Ortiz GM, Lopez A, Cruceta A, et al. Relevance of HIV-1-specific CD4+ helper T-cell responses during structured treatment interruptions in patients with CD4+ T-cell nadir above 400/mm3. Journal of acquired immune deficiency syndromes (1999). 2004;36(3):791-9.

266. Ranasinghe S, Cutler S, Davis I, Lu R, Soghoian DZ, Qi Y, et al. Association of HLA-DRB1-restricted CD4⁺ T cell responses with HIV immune control. Nature medicine. 2013;19(7):930-3.

267. Kaufmann DE, Bailey PM, Sidney J, Wagner B, Norris PJ, Johnston MN, et al. Comprehensive analysis of human immunodeficiency virus type 1-specific CD4 responses reveals marked immunodominance of gag and nef and the presence of broadly recognized peptides. J Virol. 2004;78(9):4463-77.

268. Lubong Sabado R, Kavanagh DG, Kaufmann DE, Fru K, Babcock E, Rosenberg E, et al. In vitro priming recapitulates in vivo HIV-1 specific T cell responses, revealing rapid loss of virus reactive CD4 T cells in acute HIV-1 infection. PloS one. 2009;4(1):e4256.

269. Chevalier MF, Jülg B, Pyo A, Flanders M, Ranasinghe S, Soghoian DZ, et al. HIV-1-specific interleukin-21+ CD4+ T cell responses contribute to durable viral control through the modulation of HIV-specific CD8+ T cell function. Journal of virology. 2011;85(2):733-41.

270. Ranasinghe S, Flanders M, Cutler S, Soghoian DZ, Ghebremichael M, Davis I, et al. HIV-specific CD4 T cell responses to different viral proteins have discordant associations with viral load and clinical outcome. Journal of virology. 2012;86(1):277-83.

271. Castelli FA, Szely N, Olivain A, Casartelli N, Grygar C, Schneider A, et al. Hierarchy of CD4 T cell epitopes of the ANRS Lipo5 synthetic vaccine relies on the frequencies of pre-existing peptide-specific T cells in healthy donors. Journal of immunology (Baltimore, Md : 1950). 2013;190(11):5757-63.

272. Lotti B, Wendland T, Furrer H, Yawalkar N, von Greyerz S, Schnyder K, et al. Cytotoxic HIV-1 p55gag-specific CD4+ T cells produce HIV-inhibitory cytokines and chemokines. J Clin Immunol. 2002;22(5):253-62.

273. Harcourt GC, Garrard S, Davenport MP, Edwards A, Phillips RE. HIV-1 variation diminishes CD4 T lymphocyte recognition. The Journal of experimental medicine. 1998;188(10):1785-93.

274. Bazhan SI, Karpenko LI, Lebedev LR, Uzhachenko RV, Belavin PA, Eroshkin AM, et al. A synergistic effect of a combined bivalent DNA-protein anti-HIV-1 vaccine containing multiple T- and B-cell epitopes of HIV-1 proteins. Mol Immunol. 2008;45(3):661-9.

275. Simon GG, Hu Y, Khan AM, Zhou J, Salmon J, Chikhlikar PR, et al. Dendritic cell mediated delivery of plasmid DNA encoding LAMP/HIV-1 Gag fusion immunogen enhances T cell epitope responses in HLA DR4 transgenic mice. PloS one. 2010;5(1):e8574.

276. Schieffer M, Jessen HK, Oster AF, Pissani F, Soghoian DZ, Lu R, et al. Induction of Gag-specific CD4 T cell responses during acute HIV infection is associated with improved viral control. Journal of virology. 2014;88(13):7357-66.

277. Koeppe JR, Campbell TB, Rapaport EL, Wilson CC. HIV-1-specific CD4+ T-cell responses are not associated with significant viral epitope variation in persons with persistent plasma viremia. Journal of acquired immune deficiency syndromes (1999). 2006;41(2):140-8.

278. Jones RB, Yue FY, Gu XX, Hunter DV, Mujib S, Gyenes G, et al. Human immunodeficiency virus type 1 escapes from interleukin-2-producing CD4+ T-cell responses without high-frequency fixation of mutations. Journal of virology. 2009;83(17):8722-32.

279. Fonseca SG, Coutinho-Silva A, Fonseca LA, Segurado AC, Moraes SL, Rodrigues H, et al. Identification of novel consensus CD4 T-cell epitopes from clade B HIV-1 whole genome that are frequently recognized by HIV-1 infected patients. AIDS (London, England). 2006;20(18):2263-73.

280. Geels MJ, Jansen CA, Baan E, De Cuyper IM, van Schijndel GJ, Schuitemaker H, et al. CTL escape and increased viremia irrespective of HIV-specific CD4+ T-helper responses in two HIV-infected individuals. Virology. 2006;345(1):209-19.

281. Boritz E, Palmer BE, Livingston B, Sette A, Wilson CC. Diverse repertoire of HIV-1 p24-specific, IFN-gamma-producing CD4+ T cell clones following immune reconstitution on highly active antiretroviral therapy. Journal of immunology (Baltimore, Md : 1950). 2003;170(2):1106-16.

282. Ramduth D, Day CL, Thobakgale CF, Mkhwanazi NP, de Pierres C, Reddy S, et al. Immunodominant HIV-1 Cd4+ T cell epitopes in chronic untreated clade C HIV-1 infection. PloS one. 2009;4(4):e5013.

283. Younes SA, Yassine-Diab B, Dumont AR, Boulassel MR, Grossman Z, Routy JP, et al. HIV-1 viremia prevents the establishment of interleukin 2-producing HIV-specific memory CD4+ T cells endowed with proliferative capacity. The Journal of experimental medicine. 2003;198(12):1909-22.

284. Zaunders J, Dyer WB, Churchill M, Munier CML, Cunningham PH, Suzuki K, et al. Possible clearance of transfusion-acquired nef/LTR-deleted attenuated HIV-1 infection by an elite controller with CCR5 Δ32 heterozygous and HLA-B57 genotype. J Virus Erad. 2019;5(2):73-83.

285. Malhotra U, Holte S, Dutta S, Berrey MM, Delpit E, Koelle DM, et al. Role for HLA class II molecules in HIV-1 suppression and cellular immunity following antiretroviral treatment. The Journal of clinical investigation. 2001;107(4):505-17.

286. Shu J, Shen W, Liu H, Zhou Y, Li J, Zhuang Y, et al. The immunologic dominance of an epitope within a rationally designed poly-epitope vaccine is influenced by multiple factors. Vaccine. 2020;38(14):2913-24.

287. Angin M, Streeck H, Wen F, King M, Pereyra F, Altfeld M, et al. Regulatory T cell frequencies do not correlate with breadth or magnitude of HIV-1-specific T cell responses. AIDS research and human retroviruses. 2012;28(8):749-51.

288. Statement of organization, functions, and delegations of authority--HCFA. Fed Regist. 1991;56(181):47215-6.

289. Ratto-Kim S, de Souza MS, Currier JR, Karasavvas N, Sidney J, Rolland M, et al. Identification of immunodominant CD4-restricted epitopes co-located with antibody binding sites in individuals vaccinated with ALVAC-HIV and AIDSVAX B/E. PloS one. 2015;10(2):e0115582.

290. Gartland AJ, Li S, McNevin J, Tomaras GD, Gottardo R, Janes H, et al. Analysis of HLA A*02 association with vaccine efficacy in the RV144 HIV-1 vaccine trial. Journal of virology. 2014;88(15):8242-55.

291. Rychert J, Saindon S, Placek S, Daskalakis D, Rosenberg E. Sequence variation occurs in CD4 epitopes during early HIV infection. Journal of acquired immune deficiency syndromes (1999). 2007;46(3):261-7.
